# First Look at the Venom of *Naja ashei*

**DOI:** 10.3390/molecules23030609

**Published:** 2018-03-08

**Authors:** Konrad Kamil Hus, Justyna Buczkowicz, Vladimír Petrilla, Monika Petrillová, Andrzej Łyskowski, Jaroslav Legáth, Aleksandra Bocian

**Affiliations:** 1 Department of Biotechnology and Bioinformatics, Faculty of Chemistry, Rzeszow University of Technology, Powstańców Warszawy 6, 35-959 Rzeszow, Poland; knr.hus@gmail.com (K.K.H.); czaporj@prz.edu.pl (J.B.); alyskowski@prz.edu.pl (A.Ł.); Jaroslav.Legath@uvlf.sk (J.L.); 2 Department of Physiology, University of Veterinary Medicine and Pharmacy, Komenského 73, 041 81 Kosice, Slovakia; petrillav@gmail.com; 3 Zoological Department, Zoological Garden Košice, Široká 31, 040 06 Košice-Kavečany, Slovakia; 4 Department of General Education Subjects, University of Veterinary Medicine and Pharmacy, Komenského 73, 041 81 Kosice, Slovakia; monika.petrillova@uvlf.sk; 5 Department of Pharmacology and Toxicology, University of Veterinary Medicine and Pharmacy, Komenského 73, 041 81 Kosice, Slovakia

**Keywords:** *Naja ashei*, venom composition, 2-D electrophoresis, proteomics

## Abstract

*Naja ashei* is an African spitting cobra species closely related to *N. mossambica* and *N. nigricollis*. It is known that the venom of *N. ashei*, like that of other African spitting cobras, mainly has cytotoxic effects, however data about its specific protein composition are not yet available. Thus, an attempt was made to determine the venom proteome of *N. ashei* with the use of 2-D electrophoresis and MALDI ToF/ToF (Matrix-Assisted Laser Desorption/Ionization Time of Flight) mass spectrometry techniques. Our investigation revealed that the main components of analysed venom are 3FTxs (Three-Finger Toxins) and PLA_2_s (Phospholipases A_2_). Additionally the presence of cysteine-rich venom proteins, 5′-nucleotidase and metalloproteinases has also been confirmed. The most interesting fact derived from this study is that the venom of *N. ashei* includes proteins not described previously in other African spitting cobras—cobra venom factor and venom nerve growth factor. To our knowledge, there are currently no other reports concerning this venom composition and we believe that our results will significantly increase interest in research of this species.

## 1. Introduction

Electrophoretic techniques have been extensively used during past years to analyze complex mixtures of peptides and proteins like snake venoms. Rapid development of chromatographic techniques coupled with mass spectrometry is considered as standard in modern proteomics, however two-dimensional electrophoresis still remains an important method in analysis of venom variation, post-translational modifications or whole proteome mapping. 

The African spitting cobras are widely distributed throughout the dry, open areas of sub-Saharan region. They are present from Senegal in the west to Somalia in the east, and from southern Egypt in the north to South Africa. This group comprises several snake species, including *Naja nigricollis*, *N. katiensis* and *N. pallida*. In 2007, *N. ashei* became another representative of African spitting cobras as Wüster and Broadley have classified it as a separate species [1]. In general, *N. ashei* venom has similar properties to the venoms of other African spitting cobras. It can cause local tissue damage, i.e., oedema, blistering and necrosis of the skin and subcutaneous connective tissue [2,3,4,5]. In addition, the venom is often spat into the eyes causing ophthalmic lesions [6]. After snake attack, a rapid development of tissue necrosis is observed, and in cases when antivenom treatment is administered too late, local lesions often lead to chronic ulceration, osteomyelitis, arthrodesis, hypertrophic scars, keloid formation and, in some chronic cases, malignant transformation [5].

Proteomic analysis of the spitting cobra venom composition revealed that in majority it consists of three-finger toxin (3FTx) and cytotoxic phospholipase A_2_ (PLA_2_) molecules accounting, respectively, for 67–73% and 22–30% of the total venom proteins. The third largest group of proteins are the snake venom metalloproteinases (SVMPs) from PIII subfamily. There are also some other proteins that are less universal for all African spitting cobras, for instance: nucleotidases, cysteine-rich secretory proteins (CRISPs) or nawaprin [7]. 

So far, to our knowledge, no one has undertaken an analysis of the protein or peptide composition of *Naja ashei* venom. Therefore, in our study we present for the first time our initial venom composition results determined with the use of 2-D electrophoresis coupled with MALDI ToF/ToF mass spectrometry analysis.

## 2. Results

**Figure 1:**
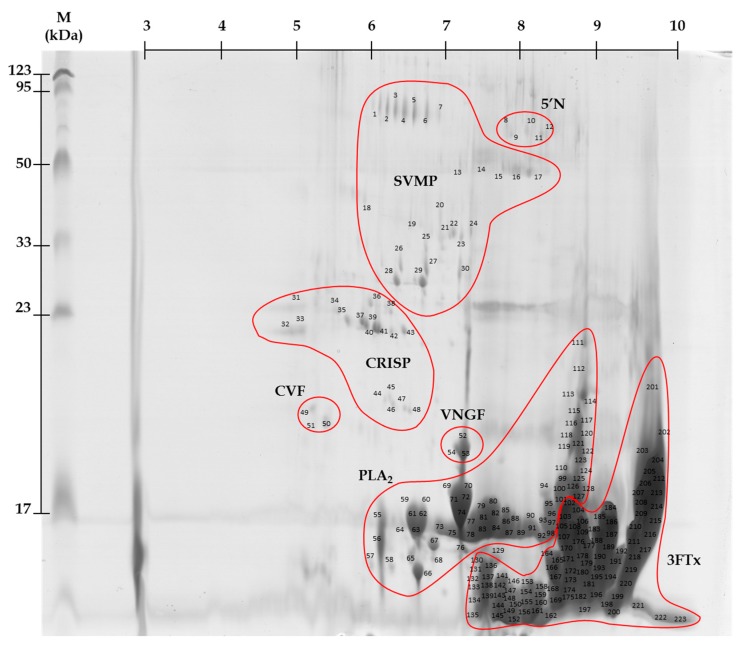
Representative 2-D protein map obtained from *Naja ashei* venom. **5′N**—Snake venom 5′-nucleotidase; **SVMP**s—Snake venom metalloproteinases; **CRISP**s—Cysteine-rich venom proteins; **CVF**—Cobra venom factor; **VNGF**—Venom nerve growth factor, **PLA_2_**s—Phospholipases A_2_; **3FTx**—Snake three-finger toxin family.

Distribution of spots on the obtained gels clearly indicated that the vast majority of proteins in the *Naja ashei* venom have a low molecular weight and clearly basic character (Figure 1). On the gel there were about 80 spots in the pH range of 3–10. The exact number was impossible to determine because of the smears, spot trains and inaccurate separation of the most abundant spots.

**Figure 2:**
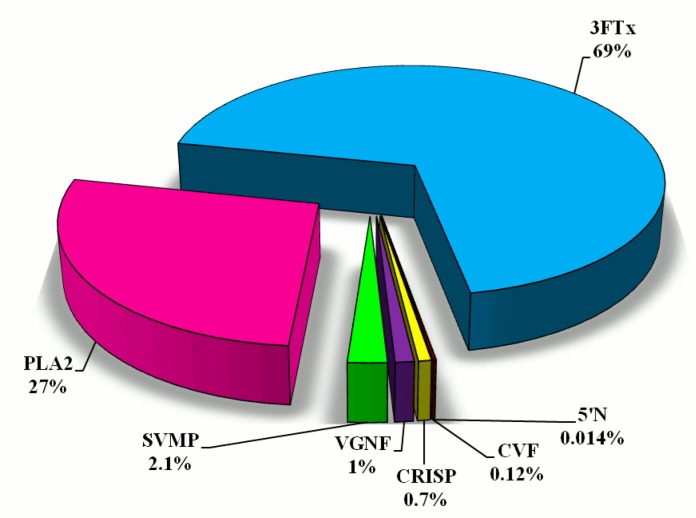
The percentage distribution of different protein groups in *Naja ashei* proteome calculated on the basis of %Vol of particular spots on gels. Abbreviations are the same as in Figure 1.

The results for protein identification using MALDI ToF/ToF mass spectrometry are summarized in Table 1. Identified proteins were grouped into seven major groups (Figure 2).

Using the %Vol of each spot on the gel, relative amounts of individual protein fractions in the venom of *N. ashei* were determined. Percentage distribution of protein groups is presented in Figure 2. According to this analysis, the most abundant proteins are cytotoxins belonging to snake three-finger toxins (almost 70%). The second highly abundant group are phospholipases A_2_ (27%). The share of other groups of proteins: metalloproteinases, venom nerve growth factor, cysteine-rich venom proteins, cobra venom factor, snake venom 5′-nucleotidase does not exceed 5% of the total protein content (Figure 2).

## 3. Discussion

Proteomic analysis of the venoms of African species of spitting cobras has revealed similar properties and protein profiles [7]. We discovered seven groups of proteins, among which 3FTxs and phospholipases A_2_ were the most abundant. The remaining five groups of proteins (SVMPs, CRISPs, venom nerve growth factor, cobra venom factor and 5′-nucleotidases) together constitute less than 5% of the total proteins of *Naja ashei* venom. For this group of Elapidae the predominant share of cytotoxic 3FTx and PLA_2_ molecules is distinctive. However, the minor contribution of SVMPs, CRISPs, and endonucleases was also described [7]. On the basis of this composition, it is likely that the major cytotoxins and PLA_2_s are responsible for the predominant myo- and cytotoxic effects induced by these venoms (i.e., dermonecrosis) [8].

A large number of three-finger toxins interfere with cholinergic transmission in the peripheral and central nervous system, thus, they are classified to the neurotoxin group [9]. However, a large number of the 3FTxs also exhibit general cytolytic properties (i.e., disruption of the membrane bilayer by forming pores in the cellular surface or penetrating into the biological membranes and triggering different biological phenomena and, therefore, they are also referred to as cytolysins or cytotoxins) [10,11,12,13]. The most interesting from a pharmacological point of view is the fact that cytotoxins possess significant and selective anticancer activity by inducing apoptosis or necrosis of tumor cells [14,15,16,17,18,19,20]. It makes this group a very interesting object of investigation, especially since the intact proteins from *Naja ashei* have never been examined. 

The second most abundant protein group in *N. ashei* venom are phospholipases A_2_ (Figure 2). In general, PLA_2_s exhibit a wide variety of physiological and pathological effects. They undeniably play a role in the digestion of prey, but also exhibit a wide spectrum of pharmacological effects, such as neurotoxicity, cardiotoxicity, myotoxicity, and anticoagulant effect [21,22,23,24,25,26,27]. Interestingly, this group of proteins has also anticancer [28,29,30,31,32] and antimicrobial properties [33,34,35,36]. In *N. ashei* venom PLA_2_s constitute 27% of all identified proteins, and this value is typical for all African spitting cobras [7].

The third group of proteins, distinctive for all African spitting cobras, are metalloproteinases. Their quantity in this group of snake venom ranges from 1.6 to 3.3% [7], and in *N. ashei* metalloproteinases share 2.1% of total venom proteins. All identified metalloproteinases belong to PIII family, and are zinc-dependent enzymes degrading plasma proteins and the extracellular matrix surrounding blood vessels, leading to local and systemic haemorrhage and coagulopathy [37,38,39]. PIII-SVMPs are present in venoms of all venomous snakes; however, their proportion in Elapid venom is much lower than in Viperid [40,41]. This fact determined that elapid SVMPs are much less understood, although it is believed that local tissue damage, haemorrhage, and complement depletion, reported after *N. nigricollis* bites, are caused by SVMP activity [2,4]. Low content of metalloproteinases in venom could indicate their minor role in the pathophysiology of envenoming. However, some studies reported that their high enzymatic activity can vastly contribute to the detrimental effects of venom [38,39,42].

CRISPs were also detected in *N. ashei* venom (Figure 1), however their content is definitely small (Figure 2). They are widely distributed among different snake venoms, and in our earlier studies, we have detected them in Viperidae venoms [41,43]. Intriguingly, this group of non-enzymatic proteins is not typical for all African spitting cobra species. Earlier works indicated their presence only in *N. nigricollis* and *N. katiensis* venoms [7]. 5′-Nucleotidase seems to be more universal for this group of snakes, because it was detected in all species except *N. nubiae* [7]. Enzymes from this group were detected in venoms of several species, always in small quantities [41,44,45]. 

It is very interesting that we were able to identify two proteins not detected before in African spitting cobras venom. They are: cobra venom factor, with 0.12% share, and venom nerve growth factor, with 1% share of total venom proteins. A negligible amount of these proteins in the venom indicates that their impact on the pathology of envenoming is low, but these proteins are extremely interesting from a pharmacological point of view. Cobra venom factor depletes complement C3 protein, and thus inhibits inflammatory and immune responses. This protein could be potentially used in several human diseases treatment, for instance: myocardial ischemia reperfusion injury, age-related macular degeneration, arthritis, paroxysmal nocturnal haemoglobinuria or lymphoma [46], and carcinoma [47]. In turn, VNGF is important for the growth, development, differentiation, and survival of neurons both in the peripheral and the central nervous systems [48], and additionally it inhibits metalloproteinase-disintegrin proteins [49]. It is known that nerve growth factors interact with some cancer cells [50,51], however the greatest hopes for their use lay in the treatment of neurodegenerative diseases [52,53,54,55].

This study shows that two-dimensional electrophoresis still can be used as an effective method for protein separation in analysis of snake venom proteome. Moreover, presented results clearly indicate that venom of *Naja ashei* is very similar to the closely related African spitting cobras. Nevertheless, the most interesting fact derived from this study is that the venom of *N. ashei* includes proteins not described so far in African spitting cobras. There are no other reports concerning this venom composition and we believe that our results will significantly increase interest in research of this species.

## 4. Materials and Methods 

Pooled *Naja ashei* venom sample was obtained from two adult snakes (male and female), which were captured and officially imported from Kenya. Venom was extracted in the Pata breeding garden near Hlohovec (Slovakia), which had been designed for conservation of the reptiles’ gene pool under the veterinary certificate No. CHEZ-TT-01. The breeding garden also serves as a quarantine station for imported animals and is an official importer of exotic animals from around the world, having the permission of the State Nature Protection of the Slovak Republic under the No. 03418/06, the trade with endangered species of wild fauna and flora and on amendments to certain laws under Law no. 237/2002 Z.z. After extraction, the venom was stored at −20 °C (transport temperature) and then moved to −80 °C for deep freezing.

The detailed procedure for proteomic analysis was described in our previous papers [41,43]. Protein concentration in crude venom was measured with 2-D Quant Kit (GE Healthcare, Little Chalfont, UK), using bovine serum albumin as a standard. The samples for isoelectrofocusing (IEF) were prepared by mixing 405 μg of proteins with standard thiourea rehydration solutions containing IPG buffers 3–10 pH range (GE Healthcare). Separation was conducted on 17 cm ReadyStrip IPG Strips with 3–10 pH gradient (Bio-Rad, Hercules, CA, USA). After IEF, the strips were incubated in equilibration buffers; one containing 1% DTT (for reduction); second containing 2.5% IAA (for alkylation). Prior to SDS-PAGE (Sodium dodecyl sulfate-polyacrylamide gel electrophoresis), gel strips were placed onto the top of 13% polyacrylamide gels (1.5 × 255 × 196 mm). Roti^®^-Mark PRESTAINED molecular weight marker (Roth, Karlsruhe, Germany) was used as a mass reference. After electrophoresis, the gels were incubated overnight in staining solution with colloidal Coomassie Brilliant Blue G-250. Quantitative analysis of individual groups of proteins was carried out in Image Master 2D Platinum software (GE Healthcare) using %Vol parameter (a ratio of the volume of a particular spot to the total volume of all spots present in the gel). The final result is an average of the spots %Vol obtained from three independent gels (technical repeats). In overall, about 200 samples were collected from 80 visible spots. Small spots were excised once, and thus each one contained a single sample. In turn, larger spots constituted for several samples due to multiple excision in different regions of the spot.

All samples were digested using Sequencing Grade Modified Trypsin (Promega, Madison, WI, USA). After digestion stage every sample was mixed in 1:1 ratio with the matrix. The matrix consisted of α-cyano-4-hydroxycinnamic acid diluted in 50% acetonitrile with 0.1% trifluoroacetic acid. The obtained peptide mixtures were analyzed on MALDI-ToF/ToF MS (Autoflex Speed, Bruker Daltonics, Billerica, MA, USA). The spectrometer was working in positive ions mode with the reflectron. The analysed ion masses ranged between 700 and 3500 Da. Calibration of the spectrometer was carried out every four samples, using standards in the range of analyzed peptides (Peptide Calibration Standards II, Bruker Daltonics). The obtained mass spectra were compared to those present in SwissProt database (The UniProt Consortium, www.uniprot.org) with the use of Mascot software. The search parameters included: mass tolerance: 0.25 Da, one incomplete cleavage allowed, alkylation of cysteine by carbamidomethylation (fixed modification), and oxidation of methionine (variable modification). Moreover, some peptides were selected for analysis in MS/MS mode. The peptides were sequenced by laser-induced dissociation (LID) using LIFT ion source. The search parameters for MS/MS data included: mass tolerance for MS mode: 0.25 Da, mass tolerance for MS/MS mode: 0.5 Da, one incomplete cleavage allowed, alkylation of cysteine by carbamidomethylation (fixed modification), and oxidation of methionine (variable modification).

## Figures and Tables

**Table 1:** Proteins identified in *Naja ashei* venom.

Gel Area ^1^	Protein Name ^2^	Protein Accession Code, Source Organism as Determined by Mascot and Spot Numbers ^2^	Mass [kDa] ^3^	Score ^4^	*m*/*z* ^5^	MS/MS-Derived Sequence/Sequence Coverage ^6^
SVMP	Zinc metalloproteinase-disintegrin-like cobrin	Q9PVK7 (*Naja kaouthia*) 20–24, 27	69	60	PMF	SC 9.5%
			69	81	1280.722	DPSYGMVEPGTK
	Zinc metalloproteinase-disintegrin-like atrase A	D5LMJ3 (*Naja atra*) 1–6	70	62	1087.732	EHQEYLLR
			70	30	1073.517	KGDDVSHCR
			70	44	1497.840	ERPQCILNKPSR
	Zinc metalloproteinase-disintegrin-like atragin	D3TTC2 (*Naja atra*) 14–17, 25, 26, 28–30	71	24	1140.664	DSCFTLNQR
			71	35	1155.607	CGDGMVCSNR
			71	46	1476.894	CPIMTNQCIALR
5′N	Snake venom 5′-nucleotidase	F8S0Z7 (*Crotalus adamanteus*) 8–12	57	48	1523.801	HGQGTGELLQVSGIK
			63	62	1389.797	LTILHTNDVHAR
			65	32	1110.568	QAFEHSVHR
CRISP	Cysteine-rich venom protein annuliferin a (fragment)	P0DL14 (*Naja annulifera*) 40, 44–48	3.6	68	1168.696	NVDFNSESTR
			3.6	96	1195.609	EIVDLHNSLR
	Cysteine-rich venom protein natrin 1	Q7T1K6 (*Naja atra*) 32, 33, 37–39, 41–43	27	80	1553.910	MEWYPEAASNAER
			27	45	1569.594	MEWYPEAASNAER
CVF	Cobra venom factor	Q91132 (*Naja kaouthia*) 49–51	185	37	1306.709	GICVAEPYEIR
			185	58	1337.885	VNDDYLIWGSR
PLA_2_	Acidic phospholipase A2 CM-I	P00602 (*Naja mossambica*) 55, 56, 59–63, 70, 71	14	60	PMF	32.2%
			14	110	1769.783	CCQVHDNCYGEAEK
	Basic phospholipase A2 1	P00603 (*Naja mossambica*) 57, 58, 65–68, 72–78	14	60	PMF	32.2%
			14	46	987.512	GTPVDDLDR
			14	72	1413.809	LGCWPYLTLYK
	Basic phospholipase A2 CM-III	P00604 (*Naja mossambica*) 79–89, 91–93, 95, 101, 113–121, 123–127	14	90	PMF	55.9%
			14	99	1374.965	YIDANYNINFK
			14	79	1512.841	CCQVHDNCYEK
			14	193	2157.377	CGAAVCNCDLVAANCFAGAR
			14	28	1282.633	CTVPSRSWWHFANYGCYCGR
VNGF	Venom nerve growth factor	P61898 (*Naja atra*) 53, 54	13	60	1127.664	NPNPEPSGCR
			13	49	1648.000	GNTVTVMENVNLDNK
			13	41	1415.821	CKNPNPEPSGCR
		Q90W38 (*Bothrops jararacussu*) 52	27	65	962.627	QYFFETK
			27	71	1363.885	ALTMEGNQASWR
			27	45	1379.914	ALTMEGNQASWR
3FTx	Cytotoxin 1	P01467 (*Naja mossambica*) [C] 103–105	7	56	PMF	45%
			7	68	1302.807	CNQLIPPFWK
		P01468 (*Naja pallida*) [C] 139, 144–147, 166–192, 200, 204–220	7	78	PMF	58.3%
			7	50	1091.463	YMCCNTDK
	Cytotoxin 2	P01469 (*Naja mossambica*) [C] 193–196, 221–223	7	59	PMF	45%
			7	50	948.463	GCIDVCPK
	Cytotoxin 4	P01452 (*Naja mossambica*) [C] 106–109, 162, 180, 206	7	40	1060.609	YVCCSTDR
	Cytotoxin 5	P25517 (*Naja mossambica*) [C] 109, 142, 148–157, 159–163, 180, 206	7	39	1118.459	YECCDTDR
	Cytotoxin 11	P62390 (*Naja annulifera*) [C] 130, 136, 138–143, 154, 159, 164	7	52	1020.337	RGCAATCPK
	Muscarinic toxin-like protein 2	P82463 (*Naja kaouthia*) [M] 131	7	69	1319.692	GCAATCPIAENR

^1^ Spot gel area name is the same as in Figure 1 and Figure 2; ^2^ Protein name and database accession number of homologous proteins and organism from which protein identification originates. In the case of 3FTx cytotoxins: [C] cytotoxin activity, [M] muscarinic toxin-like activity (according to the UniProt database, www.uniprot.org). Spot numbers are related to Figure 1; ^3^ The mass of molecule as reported by Mascot (Boston, MA, USA); ^4^ Protein identification was performed using the Mascot search with probability based Mowse score. Ions score was −10 × log(*P*), where *P* was the probability that the observed match was a random event; ^5^ Mass of precursor ion or information about PMF (Peptide Mass Fingerprinting) identification mode use; ^6^ Peptide sequence derived from LIFT analysis (Autoflex Speed, Bruker Daltonics, Billerica, MA, USA). Identification of proteins by MS/MS method was conducted by comparing obtained sequences with sequences from database. In the case of PMF identification: SC—amino acid sequence coverage for the identified proteins. Collection of annotated mass spectra is available as a Supplementary Material.

## Supplementary Materials

The following are available online, Annotated MS/MS spectra of the identified proteins.
