# Supplementary material for: First Look at the Venom of Naja ashei
Source: Molecules. 2018 Mar 8;23(3):609. doi: 10.3390/molecules23030609 (PMC6017371; doi:10.3390/molecules23030609)
Supplement: Supplementary file 1 [file molecules-23-00609-s001.zip › molecules-273268-supplementary.pdf]

SVMP parentmass 1280.722

Spectrum Analysis Report

Sequence Name:		Parentmass:	1280.722	Mass Error:	0.138	MH+ (mono):	1280.583
MH+ (avg):	1281.415	Threshold (a.i.):	0.000	Tolerance (Da):	0.700	Number of Peaks:	42
Above Threshold:	42	Assigned Peaks:	26	Not assigned Peaks:	16		

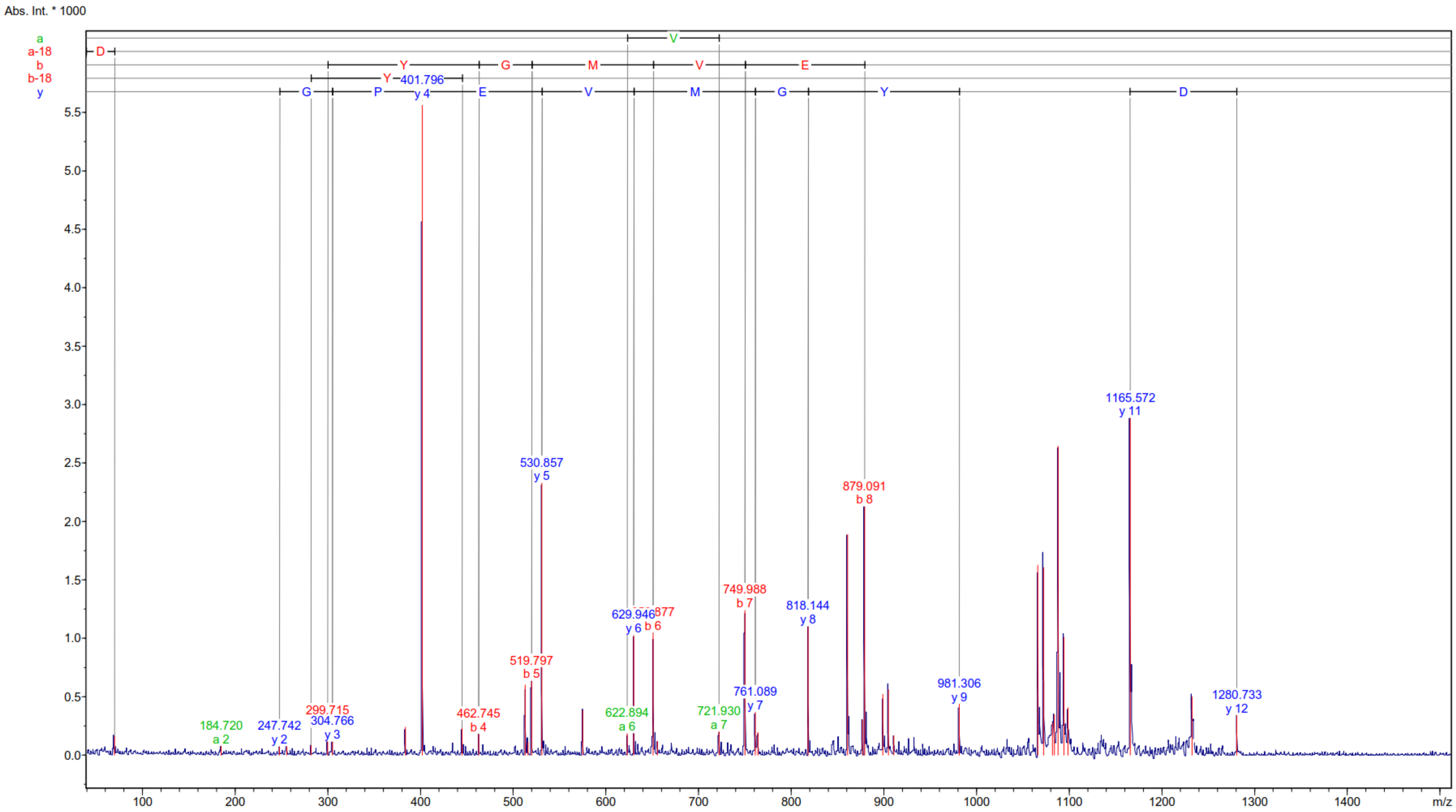

Copyright © 1999-2008 Bruker Daltonik GmbH

Spectrum Analysis Report

Display Parameter:

Parentmass:	1280.722	Mass Error:	0.138	MH+ (mono):	1280.583	MH+ (avg):	1281.415
Threshold (a.i.):	0.000	Tolerance (Da):	0.700	Number of Peaks:	42	Above Threshold:	42
Assigned Peaks:	26	Not assigned Peaks:	16				

Peaklist:

Peak	Mass	Intensity	Peak	Mass	Intensity	Peak	Mass	Intensity	Peak	Mass	Intensity	Peak	Mass	Intensity
1	69.774	160.779	2	184.720	79.616	3	247.742	75.660	4	255.685	131.149	5	281.708	83.975
6	299.715	211.987	7	304.766	116.691	8	383.783	239.234	9	401.796	5600.690	10	444.762	232.287
11	462.745	181.740	12	512.854	600.917	13	515.911	109.819	14	519.797	635.928	15	530.857	2329.960
16	574.848	384.478	17	622.894	186.002	18	629.946	1026.185	19	650.877	1047.568	20	655.617	119.095
21	721.930	202.421	22	749.988	1240.720	23	761.089	366.759	24	764.007	192.346	25	818.144	1101.213
26	860.825	1888.616	27	876.834	302.378	28	879.091	2127.536	29	898.809	520.061	30	904.801	561.660
31	910.682	167.668	32	981.306	439.932	33	1065.926	1624.417	34	1072.116	1606.274	35	1081.835	273.143
36	1083.819	346.691	37	1087.884	2644.018	38	1094.131	1009.568	39	1098.532	406.657	40	1165.572	2885.102
41	1232.364	497.836	42	1280.733	342.255									

Calculated Masses:

DPSYGMVEPGTK

N-Term.	Ion	a	a-17	a-18	b	b-17	b-18	b+18	c	i	x	y	z	C-Term.	Ion
1	D	88.039	71.013	**70.029**	116.034	99.008	98.024	134.045	133.061	88.039	173.092	147.113	130.086	12	K
2	P	**185.092**	168.066	167.082	213.087	196.060	195.076	231.098	230.114	**70.065**	274.140	**248.160**	231.134	11	T
3	S	272.124	**255.098**	254.114	**300.119**	283.092	**282.108**	318.130	317.146	60.044	331.161	**305.182**	288.155	10	G
4	Y	435.187	418.161	417.177	**463.182**	446.156	**445.172**	481.193	480.209	136.076	428.214	**402.235**	385.208	9	P
5	G	492.209	475.182	474.198	**520.204**	503.177	502.193	538.214	537.230	30.034	557.257	**531.277**	514.251	8	E
6	M	**623.249**	606.223	605.239	**651.244**	634.218	633.234	669.255	668.271	104.053	656.325	**630.346**	613.319	7	V
7	V	**722.318**	705.291	704.307	**750.313**	733.286	732.302	768.323	767.339	72.081	787.365	**761.386**	744.360	6	M
8	E	851.360	834.334	833.350	**879.355**	862.329	**861.345**	897.366	896.382	102.055	844.387	**818.408**	801.381	5	G
9	P	948.413	931.387	930.403	976.408	959.382	958.397	994.419	993.435	**70.065**	1007.450	**981.471**	964.444	4	Y
10	G	1005.435	988.408	987.424	1033.430	1016.403	1015.419	1051.440	1050.456	30.034	**1094.482**	1068.503	1051.476	3	S
11	T	1106.482	1089.456	**1088.472**	1134.477	1117.451	1116.467	1152.488	1151.504	74.060	1191.535	**1165.556**	1148.529	2	P
12	K	1234.577	1217.551	1216.567	1262.572	1245.546	1244.562	**1280.583**	1279.599	101.107	1306.562	**1280.583**	1263.556	1	D

Copyright © 1999-2008 Bruker Daltonik GmbH

SVMP parentmass 1087.732

Spectrum Analysis Report

Sequence Name:		Parentmass:	1087.732	Mass Error:	0.178	MH+ (mono):	1087.554
MH+ (avg):	1088.196	Threshold (a.i.):	0.000	Tolerance (Da):	0.700	Number of Peaks:	58
Above Threshold:	58	Assigned Peaks:	34	Not assigned Peaks:	24		

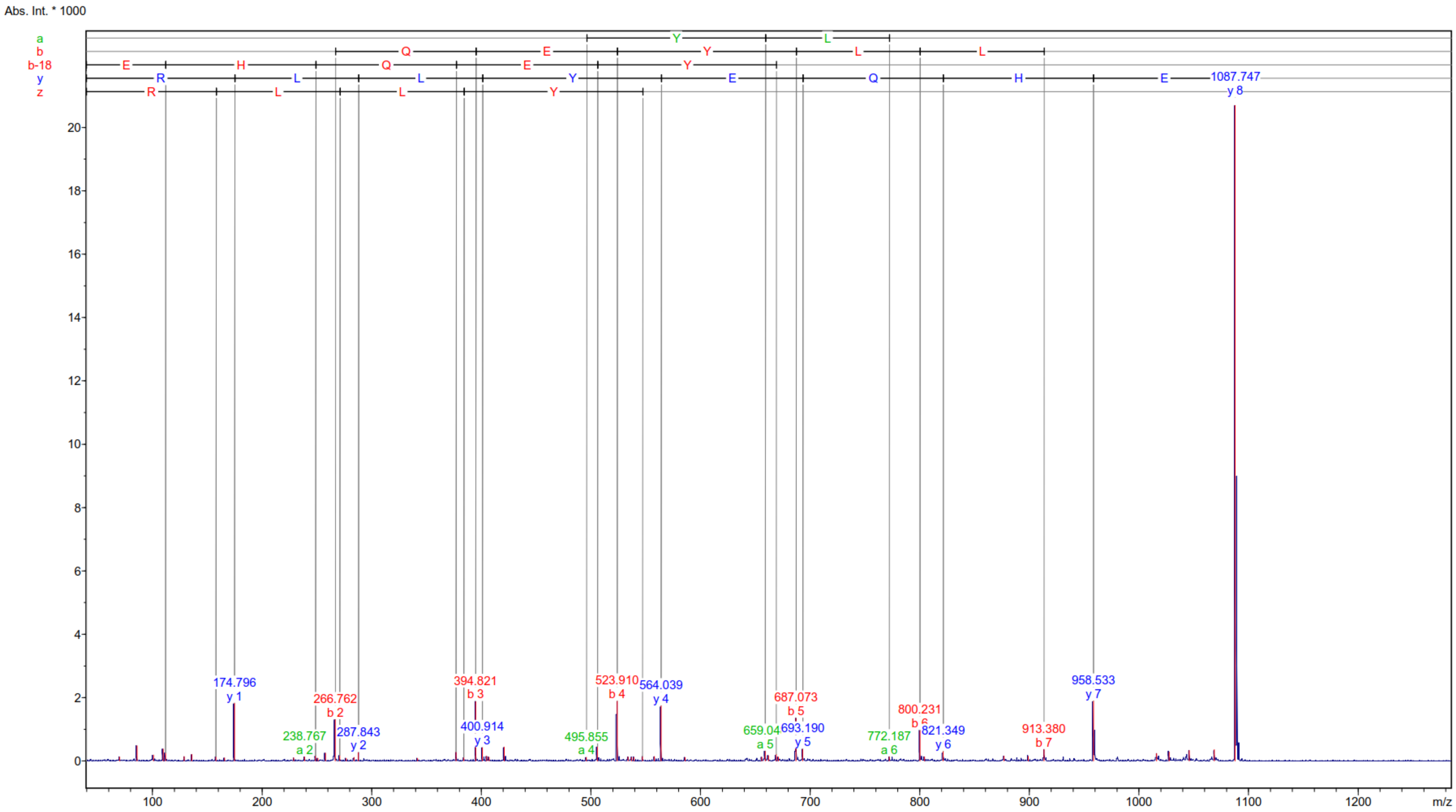

Copyright © 1999-2008 Bruker Daltonik GmbH

Spectrum Analysis Report

Display Parameter:

Parentmass:	1087.732	Mass Error:	0.178	MH+ (mono):	1087.554	MH+ (avg):	1088.196
Threshold (a.i.):	0.000	Tolerance (Da):	0.700	Number of Peaks:	58	Above Threshold:	58
Assigned Peaks:	34	Not assigned Peaks:	24				

Peaklist:

Peak	Mass	Intensity	Peak	Mass	Intensity	Peak	Mass	Intensity	Peak	Mass	Intensity	Peak	Mass	Intensity
1	69.851	123.125	2	85.836	478.636	3	100.805	186.281	4	109.792	378.274	5	111.797	252.692
6	128.803	137.018	7	135.775	205.202	8	157.775	133.173	9	165.740	96.123	10	174.796	1831.344
11	228.767	97.685	12	238.767	134.192	13	248.756	163.866	14	250.879	84.177	15	257.759	251.881
16	266.762	1315.614	17	270.835	167.385	18	276.789	87.310	19	283.781	110.285	20	287.843	277.961
21	341.862	87.804	22	376.830	284.164	23	383.901	113.757	24	394.821	1887.169	25	400.914	433.002
26	404.841	156.134	27	407.033	118.662	28	420.847	438.589	29	495.855	122.901	30	505.895	456.975
31	523.910	1899.072	32	533.888	126.998	33	536.955	112.716	34	539.093	114.709	35	546.999	137.240
36	557.940	142.190	37	564.039	1752.860	38	585.926	112.389	39	655.855	122.201	40	659.048	317.060
41	662.001	164.049	42	669.048	204.796	43	671.007	125.547	44	687.073	1363.997	45	693.190	388.876
46	772.187	143.873	47	800.231	974.532	48	804.283	132.797	49	821.349	314.828	50	876.977	156.875
51	899.022	149.722	52	913.380	370.794	53	958.533	1900.914	54	1016.145	236.890	55	1027.591	280.923
56	1045.768	328.029	57	1068.675	359.235	58	1087.747	20958.009						

Calculated Masses:

EHQEYLLR

N-Term.	Ion	a	a-17	a-18	b	b-17	b-18	b+18	c	i	x	y	z	C-Term.	Ion
1	E	102.055	85.028	84.044	130.050	113.023	**112.039**	148.060	147.076	102.055	201.098	**175.119**	**158.092**	8	R
2	H	**239.114**	222.087	221.103	**267.109**	250.082	**249.098**	285.119	**284.135**	**110.071**	314.182	**288.203**	**271.176**	7	L
3	Q	367.172	350.146	349.162	**395.167**	378.141	**377.157**	413.178	412.194	**101.071**	427.266	**401.287**	**384.261**	6	L
4	E	**496.215**	479.188	478.204	**524.210**	507.183	**506.199**	542.221	541.236	102.055	590.330	**564.350**	**547.324**	5	Y
5	Y	**659.278**	642.252	641.268	**687.273**	670.247	**669.263**	705.284	704.300	**136.076**	719.372	**693.393**	676.366	4	E
6	L	**772.362**	755.336	754.352	**800.357**	783.331	782.347	818.368	817.384	**86.096**	847.431	**821.452**	**804.425**	3	Q
7	L	885.446	868.420	867.436	**913.441**	896.415	895.431	931.452	930.468	**86.096**	984.490	**958.510**	941.484	2	H
8	R	1041.548	1024.521	1023.537	1069.543	1052.516	1051.532	**1087.553**	1086.569	**129.113**	1113.532	**1087.553**	1070.527	1	E

Copyright © 1999-2008 Bruker Daltonik GmbH

SVMP parentmass 1073.517

Spectrum Analysis Report

Sequence Name:		Parentmass:	1073.517	Mass Error:	0.038	MH+ (mono):	1073.480
MH+ (avg):	1074.153	Threshold (a.i.):	0.000	Tolerance (Da):	0.700	Number of Peaks:	50
Above Threshold:	50	Assigned Peaks:	25	Not assigned Peaks:	25		

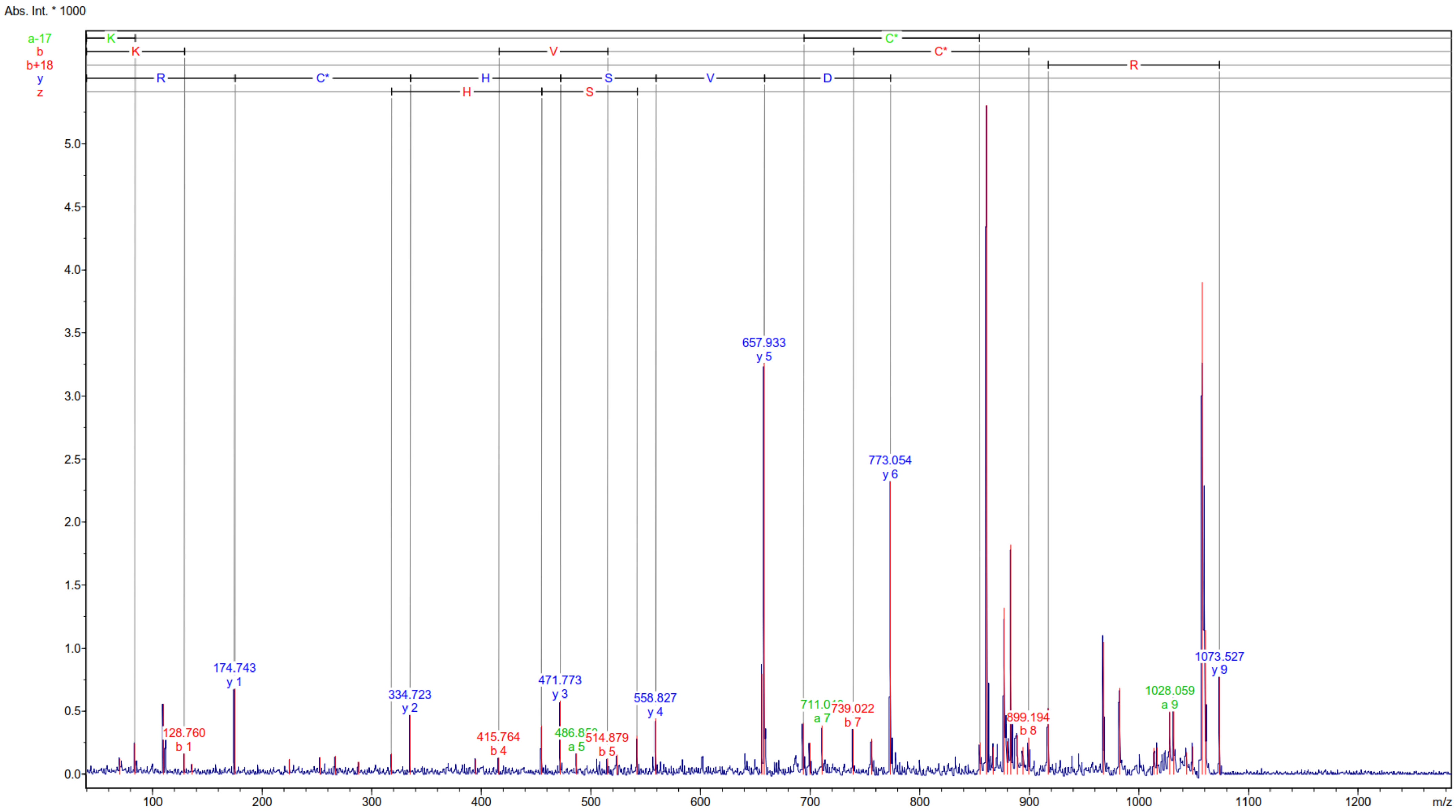

Copyright © 1999-2008 Bruker Daltonik GmbH

Spectrum Analysis Report

Display Parameter:

Parentmass:	1073.517	Mass Error:	0.038	MH+ (mono):	1073.480	MH+ (avg):	1074.153
Threshold (a.i.):	0.000	Tolerance (Da):	0.700	Number of Peaks:	50	Above Threshold:	50
Assigned Peaks:	25	Not assigned Peaks:	25				

Peaklist:

Peak	Mass	Intensity	Peak	Mass	Intensity	Peak	Mass	Intensity	Peak	Mass	Intensity	Peak	Mass	Intensity
1	69.814	121.045	2	83.799	242.850	3	109.759	555.881	4	128.760	164.610	5	135.715	78.579
6	174.743	678.294	7	224.696	119.623	8	252.763	133.607	9	266.682	143.448	10	287.708	96.718
11	317.712	163.711	12	334.723	469.356	13	394.729	115.362	14	415.764	131.749	15	454.757	387.573
16	471.773	583.592	17	486.853	164.490	18	514.879	123.964	19	523.801	153.087	20	541.815	302.307
21	558.827	439.681	22	655.691	794.818	23	657.933	3259.352	24	693.668	405.690	25	699.635	246.556
26	711.013	387.023	27	739.022	357.914	28	756.051	277.985	29	773.054	2324.330	30	854.827	249.877
31	860.858	5436.275	32	867.019	214.183	33	876.791	1317.913	34	879.842	258.312	35	882.853	1817.114
36	889.120	307.633	37	894.224	211.157	38	899.194	289.455	39	917.238	521.792	40	967.426	1044.056
41	982.415	681.641	42	1013.462	205.044	43	1016.504	208.298	44	1028.059	496.102	45	1031.516	521.645
46	1043.408	172.286	47	1049.139	216.767	48	1057.539	3901.046	49	1060.566	1140.514	50	1073.527	773.333

Calculated Masses:

KGDDVSHCR 8: Carbamidomethyl (C)

N-Term.	Ion	a	a-17	a-18	b	b-17	b-18	b+18	c	i	x	y	z	C-Term.	Ion
1	K	101.107	**84.081**	83.097	**129.102**	112.076	111.092	147.113	146.129	101.107	201.098	**175.119**	158.092	9	R
2	G	158.129	141.102	140.118	186.124	169.097	168.113	204.134	203.150	30.034	361.129	**335.150**	**318.123**	8	C*
3	D	273.156	256.129	255.145	301.151	284.124	283.140	319.161	**318.177**	88.039	498.188	**472.209**	**455.182**	7	H
4	D	388.183	371.156	370.172	**416.178**	399.151	398.167	434.188	433.204	88.039	585.220	**559.241**	**542.214**	6	S
5	V	**487.251**	470.225	469.241	**515.246**	498.219	497.235	533.257	532.273	72.081	684.288	**658.309**	641.282	5	V
6	S	574.283	557.257	556.273	602.278	585.251	584.267	620.289	619.305	60.044	799.315	**773.336**	**756.309**	4	D
7	H	**711.342**	**694.315**	**693.331**	**739.337**	722.310	721.326	757.348	**756.363**	**110.071**	914.342	888.363	871.336	3	D
8	C*	871.373	**854.346**	853.362	**899.368**	**882.341**	881.357	**917.378**	916.394	133.043	971.364	945.384	928.358	2	G
9	R	**1027.474**	1010.447	1009.463	1055.469	1038.442	1037.458	**1073.479**	1072.495	**129.113**	1099.459	**1073.479**	1056.453	1	K

Copyright © 1999-2008 Bruker Daltonik GmbH

SVMP parentmass 1497.840

Spectrum Analysis Report

Sequence Name:		Parentmass:	1497.840	Mass Error:	0.044	MH+ (mono):	1497.796
MH+ (avg):	1498.734	Threshold (a.i.):	0.000	Tolerance (Da):	0.800	Number of Peaks:	45
Above Threshold:	45	Assigned Peaks:	28	Not assigned Peaks:	17		

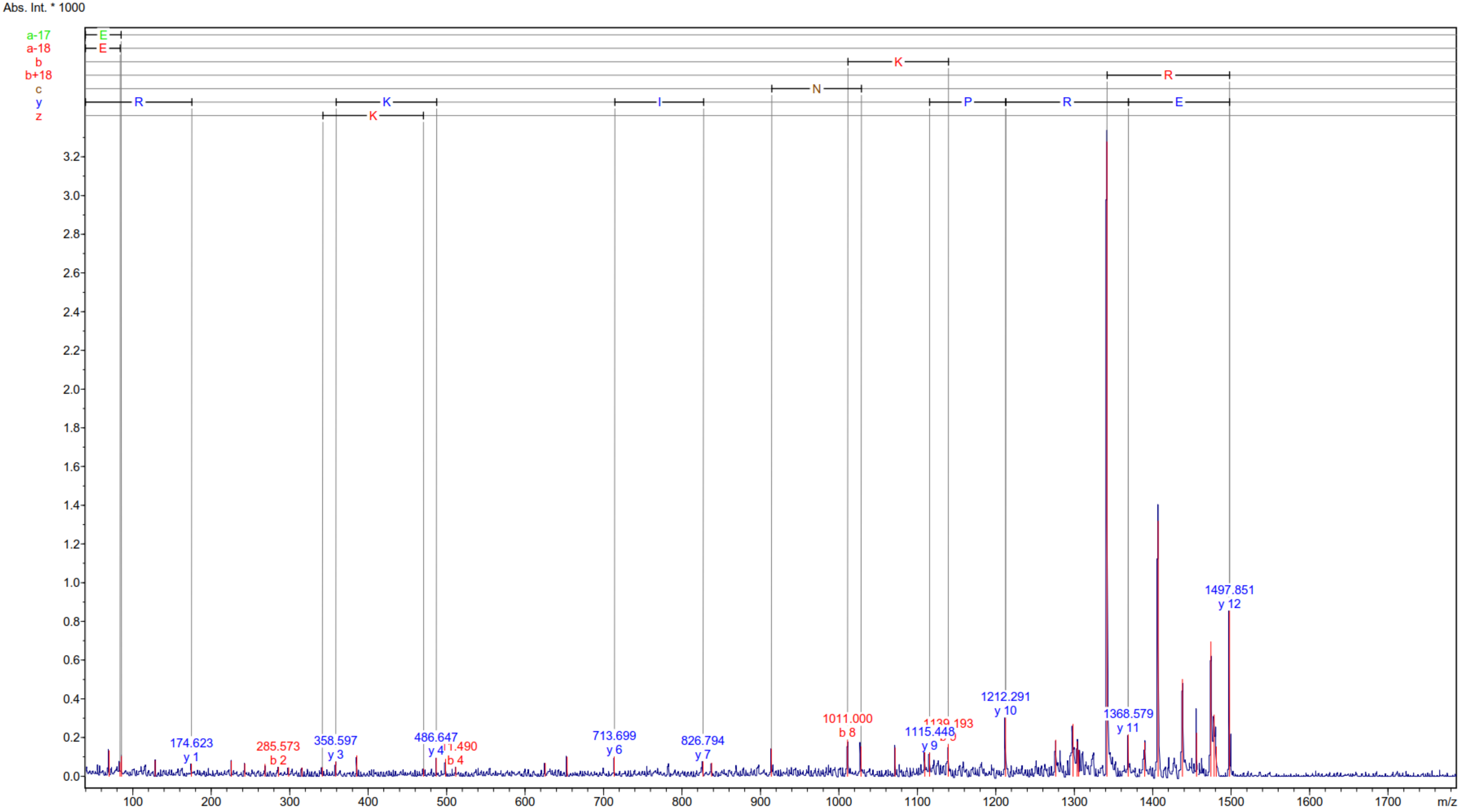

Copyright © 1999-2008 Bruker Daltonik GmbH

Spectrum Analysis Report

Display Parameter:

Parentmass:	1497.840	Mass Error:	0.044	MH+ (mono):	1497.796	MH+ (avg):	1498.734
Threshold (a.i.):	0.000	Tolerance (Da):	0.800	Number of Peaks:	45	Above Threshold:	45
Assigned Peaks:	28	Not assigned Peaks:	17				

Peaklist:

Peak	Mass	Intensity	Peak	Mass	Intensity	Peak	Mass	Intensity	Peak	Mass	Intensity	Peak	Mass	Intensity
1	69.759	132.699	2	83.705	71.879	3	85.726	104.658	4	128.615	81.912	5	174.623	64.524
6	225.583	79.064	7	242.612	66.117	8	268.572	63.207	9	285.573	47.808	10	298.647	45.005
11	315.576	44.327	12	341.569	46.524	13	358.597	76.553	14	385.510	106.737	15	470.618	40.755
16	486.647	94.975	17	498.588	88.745	18	511.490	48.118	19	625.565	67.516	20	653.601	100.745
21	713.699	101.883	22	826.794	77.339	23	837.823	68.661	24	913.920	143.086	25	1011.000	189.215
26	1028.036	155.697	27	1071.469	147.537	28	1109.505	134.892	29	1115.448	123.204	30	1139.193	166.731
31	1212.291	302.836	32	1276.268	187.955	33	1298.277	269.596	34	1303.532	179.651	35	1305.605	135.952
36	1341.482	3279.643	37	1368.579	215.985	38	1389.705	175.936	39	1406.666	1319.213	40	1437.818	501.273
41	1455.828	223.843	42	1473.963	695.550	43	1478.048	317.654	44	1480.936	152.911	45	1497.851	855.800

Calculated Masses:

ERPQCILNKPSR 5: Carbamidomethyl (C)

N-Term.	Ion	a	a-17	a-18	b	b-17	b-18	b+18	c	i	x	y	z	C-Term.	Ion
1	E	102.055	**85.028**	**84.044**	130.050	113.023	112.039	148.060	147.076	102.055	201.098	**175.119**	158.092	12	R
2	R	258.156	241.130	240.145	**286.151**	**269.124**	**268.140**	304.162	303.178	**129.113**	288.130	262.151	245.124	11	S
3	P	355.209	338.182	337.198	383.204	366.177	365.193	401.214	400.230	**70.065**	**385.183**	**359.204**	**342.177**	10	P
4	Q	483.267	466.241	465.257	**511.262**	494.236	493.252	529.273	528.289	101.071	513.278	**487.299**	**470.272**	9	K
5	C*	643.298	**626.272**	**625.287**	671.293	**654.266**	**653.282**	689.304	688.320	133.043	627.321	601.342	584.315	8	N
6	I	756.382	739.356	738.372	784.377	767.350	766.366	802.388	801.404	**86.096**	740.405	**714.426**	697.399	7	L
7	L	869.466	852.440	851.456	897.461	880.435	879.451	915.472	**914.488**	**86.096**	853.489	**827.510**	810.483	6	I
8	N	983.509	966.483	965.499	**1011.504**	994.477	993.493	1029.515	**1028.531**	87.055	1013.520	987.540	970.514	5	C*
9	K	1111.604	1094.578	1093.594	**1139.599**	1122.572	1121.588	1157.610	1156.626	101.107	1141.578	**1115.599**	1098.572	4	Q
10	P	1208.657	1191.630	1190.646	1236.652	1219.625	1218.641	1254.662	1253.678	**70.065**	1238.631	**1212.652**	1195.625	3	P
11	S	1295.689	1278.662	1277.678	1323.684	1306.657	**1305.673**	**1341.694**	**1340.710**	60.044	1394.732	**1368.753**	1351.726	2	R
12	R	1451.790	1434.763	1433.779	1479.785	1462.758	1461.774	**1497.795**	1496.811	**129.113**	1523.775	**1497.795**	**1480.769**	1	E

Copyright © 1999-2008 Bruker Daltonik GmbH

SVMP parentmass 1140.664

Spectrum Analysis Report

Sequence Name:		Parentmass:	1140.664	Mass Error:	0.153	MH+ (mono):	1140.511
MH+ (avg):	1141.239	Threshold (a.i.):	0.000	Tolerance (Da):	0.700	Number of Peaks:	29
Above Threshold:	29	Assigned Peaks:	12	Not assigned Peaks:	17		

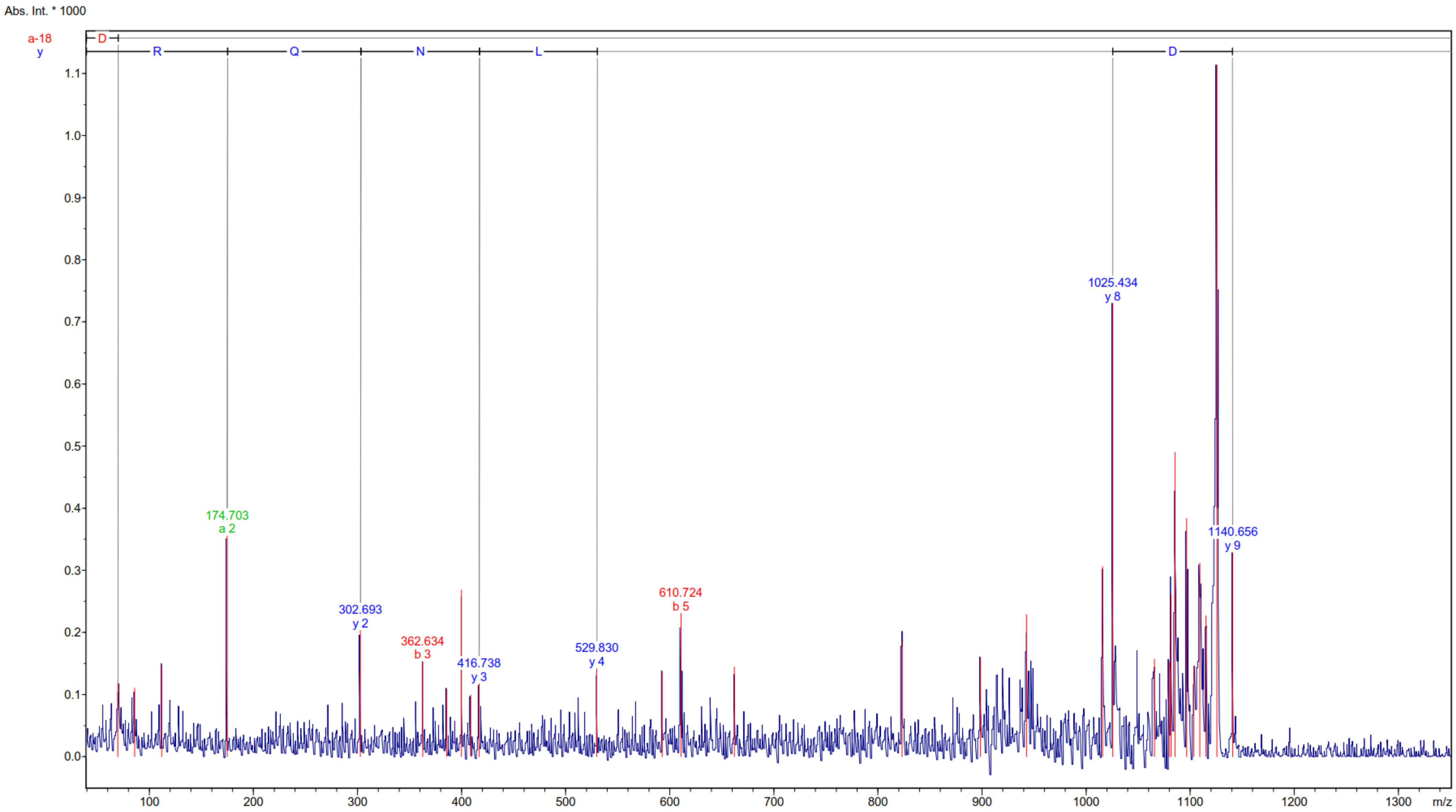

Copyright © 1999-2008 Bruker Daltonik GmbH

Spectrum Analysis Report

Display Parameter:

Parentmass:	1140.664	Mass Error:	0.153	MH+ (mono):	1140.511	MH+ (avg):	1141.239
Threshold (a.i.):	0.000	Tolerance (Da):	0.700	Number of Peaks:	29	Above Threshold:	29
Assigned Peaks:	12	Not assigned Peaks:	17				

Peaklist:

Peak	Mass	Intensity	Peak	Mass	Intensity	Peak	Mass	Intensity	Peak	Mass	Intensity	Peak	Mass	Intensity
1	69.758	117.978	2	85.781	110.530	3	111.750	147.034	4	174.703	355.887	5	302.693	203.423
6	362.634	153.346	7	385.639	108.247	8	399.698	268.322	9	408.629	99.313	10	416.738	117.905
11	529.830	141.868	12	592.737	137.614	13	610.724	231.035	14	662.016	144.661	15	823.040	185.780
16	898.456	159.304	17	942.754	228.908	18	1015.682	305.940	19	1025.434	730.288	20	1065.512	157.151
21	1079.659	152.066	22	1081.547	260.478	23	1085.386	489.842	24	1096.572	383.720	25	1103.399	145.616
26	1109.245	312.077	27	1115.082	226.823	28	1125.598	1154.170	29	1140.656	328.887			

Calculated Masses:
DSCFTLNQR 3: Carbamidomethyl (C)

N-Term.	Ion	a	a-17	a-18	b	b-17	b-18	b+18	c	i	x	y	z	C-Term.	Ion
1	D	88.039	71.013	**70.029**	116.034	99.008	98.024	134.045	133.061	88.039	201.098	**175.119**	158.092	9	R
2	S	**175.071**	158.045	157.061	203.066	186.040	185.056	221.077	220.093	60.044	329.157	**303.178**	286.151	8	Q
3	C*	335.102	318.075	317.091	**363.097**	346.070	345.086	381.107	380.123	133.043	443.200	**417.220**	**400.194**	7	N
4	F	482.170	465.144	464.160	510.165	493.139	492.155	528.176	527.192	120.081	556.284	**530.305**	513.278	6	L
5	T	583.218	566.192	565.208	**611.213**	594.186	**593.202**	629.224	628.240	74.060	657.331	631.352	614.326	5	T
6	L	696.302	679.276	678.292	724.297	707.271	706.286	742.308	741.324	**86.096**	804.400	778.421	761.394	4	F
7	N	810.345	793.319	792.334	838.340	821.313	820.329	856.351	855.367	87.055	964.431	938.451	921.425	3	C*
8	Q	938.404	921.377	920.393	966.399	949.372	948.388	984.409	983.425	101.071	1051.463	**1025.483**	1008.457	2	S
9	R	1094.505	1077.478	1076.494	1122.500	1105.473	1104.489	**1140.510**	1139.526	129.113	1166.489	**1140.510**	1123.484	1	D

Copyright © 1999-2008 Bruker Daltonik GmbH

SVMP parentmass 1155.607

Spectrum Analysis Report

Sequence Name:		Parentmass:	1155.607	Mass Error:	0.172	MH+ (mono):	1155.435
MH+ (avg):	1156.300	Threshold (a.i.):	0.000	Tolerance (Da):	0.700	Number of Peaks:	41
Above Threshold:	41	Assigned Peaks:	23	Not assigned Peaks:	18		

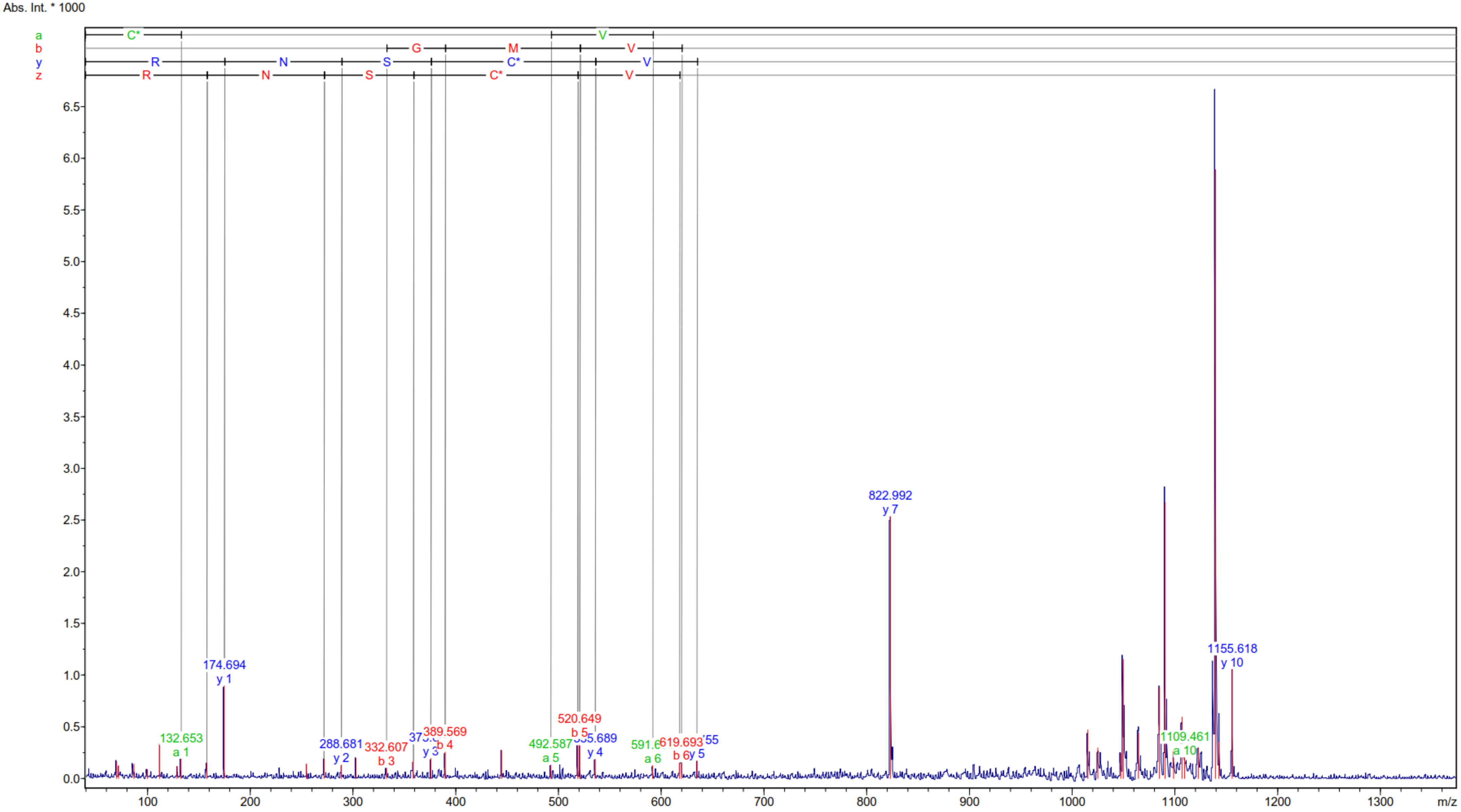

Copyright © 1999-2008 Bruker Daltonik GmbH

Spectrum Analysis Report

Display Parameter:

Parentmass:	1155.607	Mass Error:	0.172	MH+ (mono):	1155.435	MH+ (avg):	1156.300
Threshold (a.i.):	0.000	Tolerance (Da):	0.700	Number of Peaks:	41	Above Threshold:	41
Assigned Peaks:	23	Not assigned Peaks:	18				

Peaklist:

Peak	Mass	Intensity	Peak	Mass	Intensity	Peak	Mass	Intensity	Peak	Mass	Intensity	Peak	Mass	Intensity
1	69.747	162.366	2	71.778	124.576	3	86.749	139.472	4	99.752	87.669	5	111.725	336.584
6	128.728	118.309	7	132.653	189.507	8	157.669	152.840	9	174.694	897.290	10	254.658	141.123
11	271.667	194.336	12	288.681	131.913	13	302.728	197.452	14	332.607	101.339	15	358.672	167.820
16	375.663	195.817	17	389.569	257.255	18	444.636	272.503	19	492.587	133.767	20	518.678	398.839
21	520.649	381.381	22	535.689	188.228	23	591.685	126.075	24	617.763	149.797	25	619.693	153.785
26	634.755	169.965	27	822.992	2534.989	28	1015.041	470.810	29	1024.874	297.382	30	1047.160	246.293
31	1049.478	1153.824	32	1064.325	440.792	33	1084.810	884.209	34	1090.339	2671.935	35	1098.558	271.506
36	1106.893	594.635	37	1109.461	202.238	38	1122.594	300.081	39	1139.343	5891.920	40	1142.727	350.622
41	1155.618	1057.358												

Calculated Masses:
CGDGMVCSNR 1: Carbamidomethyl (C) 7: Carbamidomethyl (C)

N-Term.	Ion	a	a-17	a-18	b	b-17	b-18	b+18	c	i	x	y	z	C-Term.	Ion
1	C*	**133.043**	116.016	115.032	161.038	144.011	143.027	179.048	178.064	**133.043**	201.098	**175.119**	**158.092**	10	R
2	G	190.064	173.038	172.054	218.059	201.033	200.049	236.070	235.086	30.034	315.141	**289.162**	**272.135**	9	N
3	D	305.091	**288.065**	287.081	**333.086**	316.060	315.076	351.097	350.113	88.039	402.173	**376.194**	**359.167**	8	S
4	G	362.113	345.086	344.102	**390.108**	373.081	372.097	408.118	407.134	30.034	562.204	**536.225**	**519.198**	7	C*
5	M	**493.153**	476.127	475.143	**521.148**	504.122	503.138	539.159	538.175	104.053	661.272	**635.293**	**618.266**	6	V
6	V	**592.222**	575.195	574.211	**620.217**	603.190	602.206	638.227	637.243	**72.081**	792.313	766.333	749.307	5	M
7	C*	752.252	735.226	734.242	780.247	763.221	762.237	798.258	797.274	**133.043**	849.334	**823.355**	806.328	4	G
8	S	839.284	822.258	821.274	867.279	850.253	849.269	885.290	884.306	60.044	964.361	938.382	921.355	3	D
9	N	953.327	936.301	935.317	981.322	964.296	963.312	999.333	998.349	**87.055**	1021.383	995.403	978.377	2	G
10	R	**1109.428**	1092.402	1091.418	1137.423	1120.397	1119.413	**1155.434**	1154.450	**129.113**	1181.413	**1155.434**	1138.407	1	C*

Copyright © 1999-2008 Bruker Daltonik GmbH

SVMP parentmass 1476.894

Spectrum Analysis Report

Sequence Name:		Parentmass:	1476.894	Mass Error:	0.181	MH+ (mono):	1476.713
MH+ (avg):	1477.802	Threshold (a.i.):	0.000	Tolerance (Da):	0.700	Number of Peaks:	44
Above Threshold:	44	Assigned Peaks:	26	Not assigned Peaks:	18		

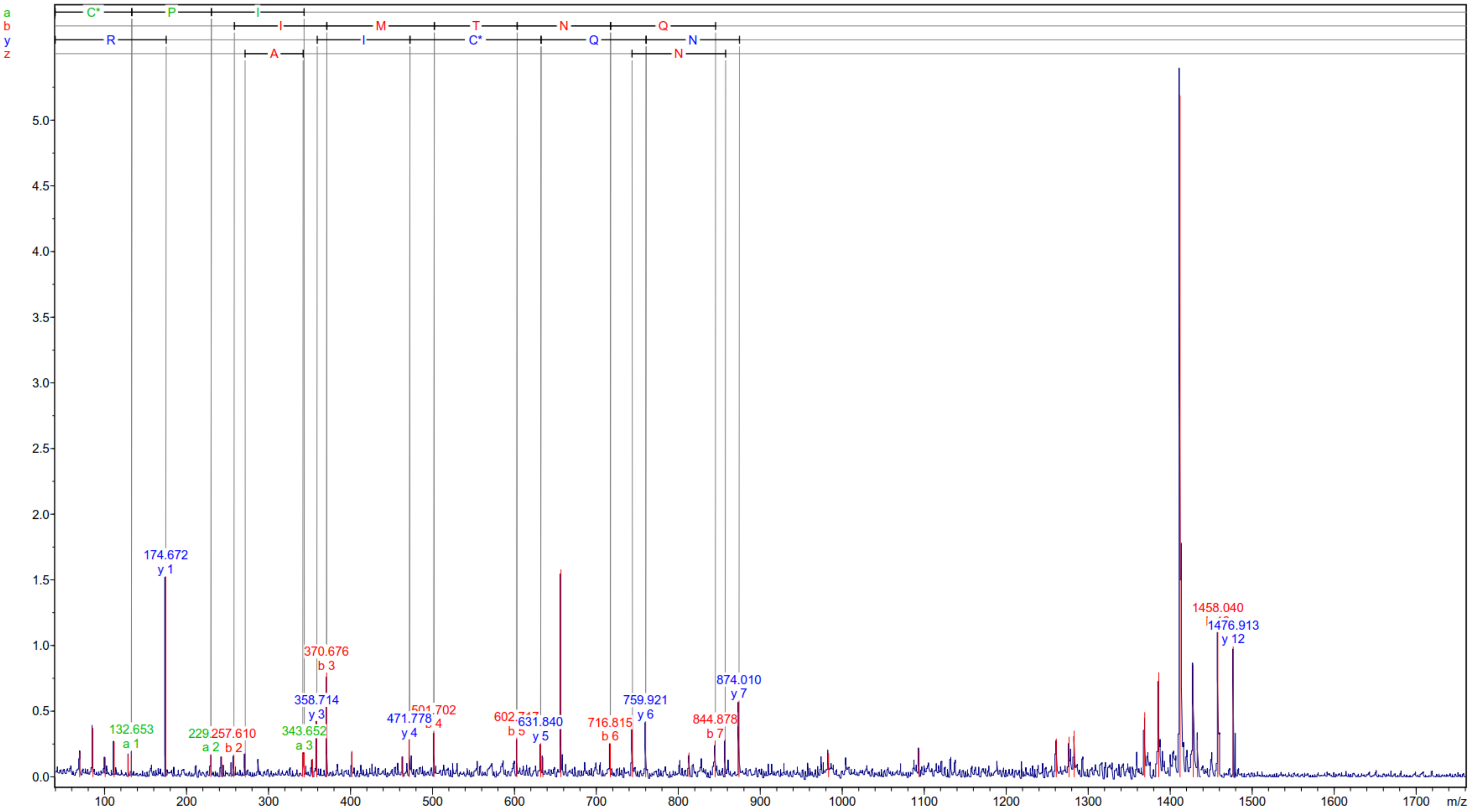

Copyright © 1999-2008 Bruker Daltonik GmbH

Spectrum Analysis Report

Display Parameter:

Parentmass:	1476.894	Mass Error:	0.181	MH+ (mono):	1476.713	MH+ (avg):	1477.802
Threshold (a.i.):	0.000	Tolerance (Da):	0.700	Number of Peaks:	44	Above Threshold:	44
Assigned Peaks:	26	Not assigned Peaks:	18				

Peaklist:

Peak	Mass	Intensity	Peak	Mass	Intensity	Peak	Mass	Intensity	Peak	Mass	Intensity	Peak	Mass	Intensity
1	69.733	184.079	2	85.773	369.792	3	100.721	147.088	4	111.704	264.537	5	128.685	164.514
6	132.653	199.134	7	174.672	1525.968	8	229.624	171.141	9	242.638	149.876	10	254.673	108.788
11	257.610	168.820	12	271.653	179.373	13	341.685	188.746	14	343.652	186.244	15	353.666	135.809
16	358.714	423.474	17	370.676	793.438	18	401.699	196.218	19	463.697	152.573	20	471.778	284.377
21	501.702	348.660	22	602.717	295.970	23	631.840	255.165	24	634.692	147.808	25	656.768	1577.591
26	716.815	253.921	27	743.788	391.808	28	759.921	422.155	29	812.884	183.420	30	844.878	275.455
31	856.948	292.320	32	874.010	578.606	33	983.090	179.219	34	1093.654	203.557	35	1260.889	290.586
36	1276.579	303.794	37	1282.812	348.946	38	1368.754	490.808	39	1385.916	793.902	40	1411.857	5187.663
41	1427.925	848.327	42	1433.086	280.959	43	1458.040	1127.796	44	1476.913	994.028			

Calculated Masses:

CPIMTNQCIALR 1: Carbamidomethyl (C) 8: Carbamidomethyl (C)

N-Term.	Ion	a	a-17	a-18	b	b-17	b-18	b+18	c	i	x	y	z	C-Term.	Ion
1	C*	**133.043**	116.016	115.032	161.038	144.011	143.027	179.048	178.064	**133.043**	201.098	**175.119**	158.092	12	R
2	P	**230.096**	213.069	212.085	**258.091**	241.064	240.080	276.101	275.117	**70.065**	314.182	288.203	**271.176**	11	L
3	I	**343.180**	326.153	325.169	**371.175**	**354.148**	**353.164**	389.185	388.201	**86.096**	385.219	**359.240**	**342.214**	10	A
4	M	474.220	457.194	456.210	**502.215**	485.189	484.205	520.226	519.242	104.053	498.303	**472.324**	455.298	9	I
5	T	575.268	558.241	557.257	**603.263**	586.236	585.252	621.273	620.289	74.060	658.334	**632.355**	615.328	8	C*
6	N	689.311	672.284	671.300	**717.306**	700.279	699.295	735.316	734.332	87.055	786.393	**760.413**	**743.387**	7	Q
7	Q	817.370	800.343	799.359	**845.364**	828.338	827.354	863.375	862.391	**101.071**	900.436	**874.456**	**857.430**	6	N
8	C*	977.400	960.374	959.390	1005.395	988.369	987.385	1023.406	1022.422	**133.043**	1001.483	975.504	958.477	5	T
9	I	1090.484	1073.458	1072.474	1118.479	1101.453	1100.469	1136.490	1135.506	**86.096**	1132.524	1106.545	1089.518	4	M
10	A	1161.521	1144.495	1143.511	1189.516	1172.490	1171.506	1207.527	1206.543	44.049	1245.608	1219.629	1202.602	3	I
11	L	1274.605	1257.579	1256.595	1302.600	1285.574	1284.590	1320.611	1319.627	**86.096**	1342.661	1316.681	1299.655	2	P
12	R	1430.707	1413.680	1412.696	**1458.701**	1441.675	1440.691	**1476.712**	1475.728	**129.113**	1502.691	**1476.712**	1459.685	1	C*

Copyright © 1999-2008 Bruker Daltonik GmbH

5`N parentmass 1523.801

Spectrum Analysis Report

Sequence Name:		Parentmass:	1523.801	Mass Error:	-0.017	MH+ (mono):	1523.818
MH+ (avg):	1524.701	Threshold (a.i.):	0.000	Tolerance (Da):	0.700	Number of Peaks:	36
Above Threshold:	36	Assigned Peaks:	17	Not assigned Peaks:	19		

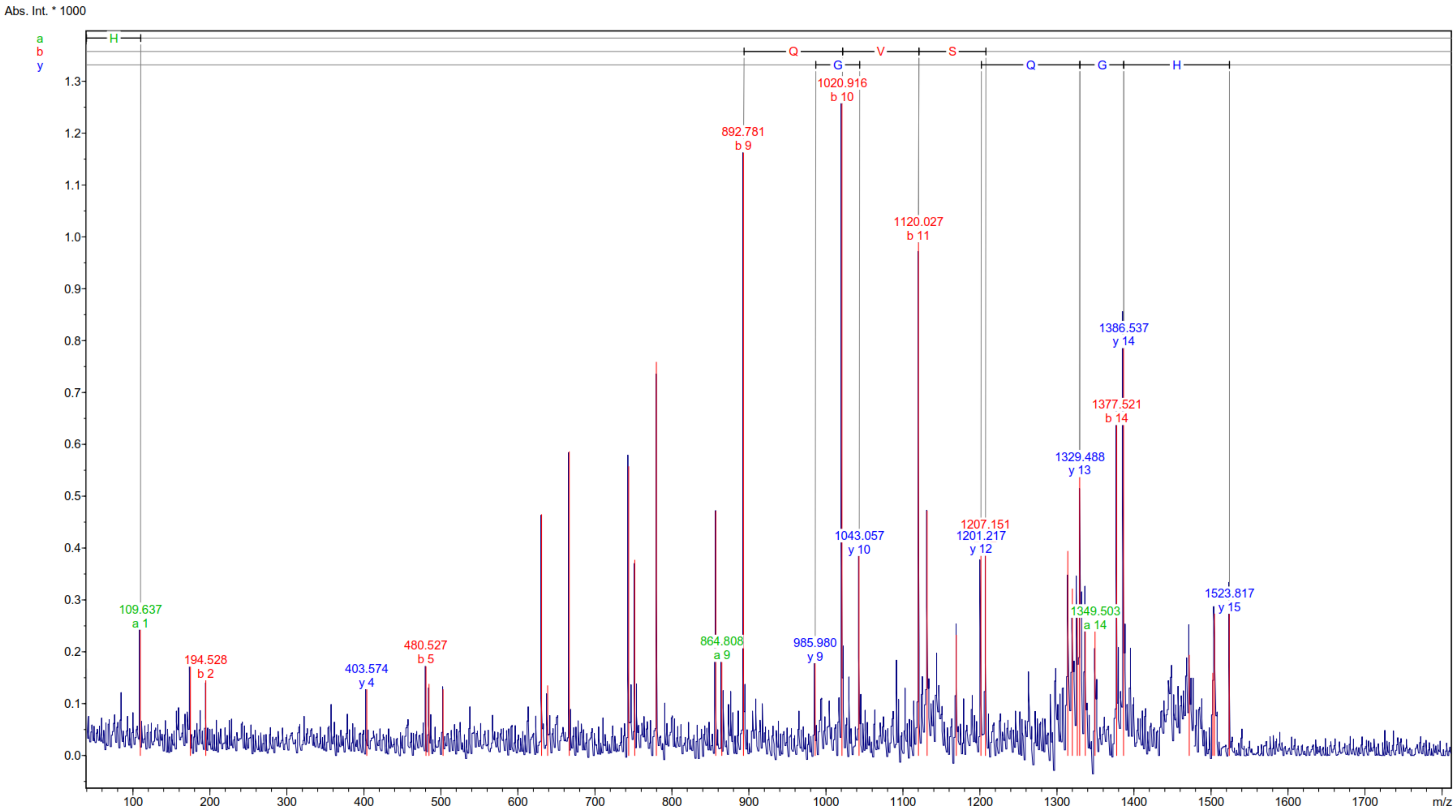

Copyright © 1999-2008 Bruker Daltonik GmbH

Spectrum Analysis Report

Display Parameter:

Parentmass:	1523.801	Mass Error:	-0.017	MH+ (mono):	1523.818	MH+ (avg):	1524.701
Threshold (a.i.):	0.000	Tolerance (Da):	0.700	Number of Peaks:	36	Above Threshold:	36
Assigned Peaks:	17	Not assigned Peaks:	19				

Peaklist:

Peak	Mass	Intensity	Peak	Mass	Intensity	Peak	Mass	Intensity	Peak	Mass	Intensity	Peak	Mass	Intensity
1	109.637	241.928	2	174.573	189.359	3	194.528	144.754	4	403.574	127.319	5	480.527	172.090
6	484.582	137.566	7	502.619	127.213	8	630.656	464.985	9	638.566	134.853	10	666.551	585.963
11	743.742	557.366	12	751.664	376.896	13	779.656	758.621	14	856.845	471.622	15	864.808	180.033
16	892.781	1162.605	17	985.980	177.564	18	1020.916	1257.279	19	1043.057	384.251	20	1120.027	989.351
21	1131.269	472.088	22	1169.208	232.371	23	1201.217	384.599	24	1207.151	405.909	25	1314.168	394.158
26	1320.035	321.423	27	1326.359	289.490	28	1329.488	536.513	29	1336.904	282.804	30	1349.503	238.875
31	1377.521	636.726	32	1386.537	785.079	33	1471.887	193.530	34	1502.471	158.946	35	1504.751	272.981
36	1523.817	273.137												

Calculated Masses:
HGQGTGELLQVSGIK

N-Term.	Ion	a	a-17	a-18	b	b-17	b-18	b+18	c	i	x	y	z	C-Term.	Ion
1	H	**110.071**	93.045	92.061	138.066	121.040	120.056	156.077	155.093	**110.071**	173.092	147.113	130.086	15	K
2	G	167.093	150.066	149.082	**195.088**	178.061	177.077	213.098	212.114	30.034	286.176	260.197	243.170	14	I
3	Q	295.151	278.125	277.141	323.146	306.120	305.136	341.157	340.173	101.071	343.198	317.218	300.192	13	G
4	G	352.173	335.146	334.162	380.168	363.141	362.157	398.178	397.194	30.034	430.230	**404.250**	387.224	12	S
5	T	453.220	436.194	435.210	**481.215**	464.189	463.205	499.226	498.242	74.060	529.298	503.319	486.292	11	V
6	G	510.242	493.215	492.231	538.237	521.210	520.226	556.247	555.263	30.034	657.357	631.377	614.351	10	Q
7	E	639.285	622.258	621.274	667.279	650.253	649.269	685.290	684.306	102.055	770.441	744.461	727.435	9	L
8	L	752.369	735.342	734.358	780.363	763.337	762.353	798.374	797.390	86.096	883.525	857.545	840.519	8	L
9	L	**865.453**	848.426	847.442	**893.448**	876.421	875.437	911.458	910.474	86.096	1012.567	**986.588**	969.562	7	E
10	Q	993.511	976.485	975.501	**1021.506**	1004.480	1003.496	1039.517	1038.533	101.071	1069.589	**1043.610**	1026.583	6	G
11	V	1092.580	1075.553	1074.569	**1120.575**	1103.548	1102.564	1138.585	1137.601	72.081	1170.636	1144.657	1127.631	5	T
12	S	1179.612	1162.585	1161.601	**1207.607**	1190.580	1189.596	1225.617	1224.633	60.044	1227.658	**1201.679**	1184.652	4	G
13	G	1236.633	1219.607	1218.623	1264.628	1247.601	1246.617	1282.639	1281.655	30.034	1355.717	**1329.737**	1312.711	3	Q
14	I	**1349.717**	1332.691	1331.707	**1377.712**	1360.686	1359.702	1395.723	1394.739	86.096	1412.738	**1386.759**	1369.732	2	G
15	K	1477.812	1460.786	1459.802	1505.807	1488.781	1487.797	**1523.818**	1522.834	101.107	1549.797	**1523.818**	1506.791	1	H

Copyright © 1999-2008 Bruker Daltonik GmbH

5`N parentmass 1389.797

Spectrum Analysis Report

Sequence Name:		Parentmass:	1389.797	Mass Error:	0.037	MH+ (mono):	1389.760
MH+ (avg):	1390.570	Threshold (a.i.):	0.000	Tolerance (Da):	0.700	Number of Peaks:	24
Above Threshold:	24	Assigned Peaks:	19	Not assigned Peaks:	5		

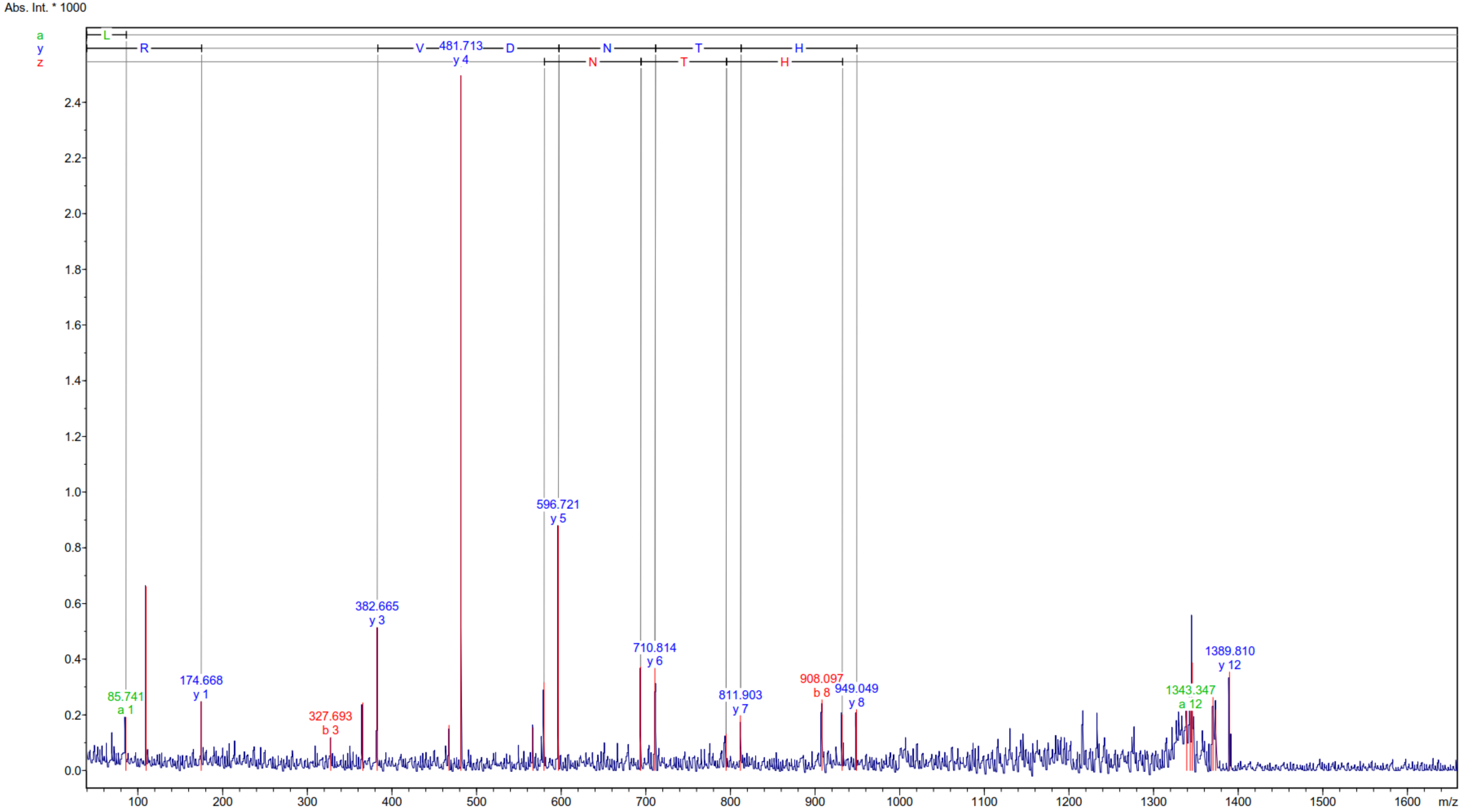

Copyright © 1999-2008 Bruker Daltonik GmbH

Spectrum Analysis Report

Display Parameter:

Parentmass:	1389.797	Mass Error:	0.037	MH+ (mono):	1389.760	MH+ (avg):	1390.570
Threshold (a.i.):	0.000	Tolerance (Da):	0.700	Number of Peaks:	24	Above Threshold:	24
Assigned Peaks:	19	Not assigned Peaks:	5				

Peaklist:

Peak	Mass	Intensity	Peak	Mass	Intensity	Peak	Mass	Intensity	Peak	Mass	Intensity	Peak	Mass	Intensity
1	85.741	192.805	2	109.684	660.205	3	174.668	248.347	4	327.693	119.700	5	365.659	243.837
6	382.665	514.984	7	467.619	162.425	8	481.713	2527.944	9	566.669	153.713	10	579.744	316.232
11	596.721	880.908	12	693.796	374.058	13	710.814	367.729	14	794.870	133.438	15	811.903	197.830
16	908.097	255.090	17	932.078	201.200	18	949.049	219.975	19	1339.128	224.935	20	1343.347	214.523
21	1345.896	386.670	22	1370.259	266.797	23	1373.258	217.702	24	1389.810	354.707			

Calculated Masses:

LTILHTNDVHAR

N-Term.	Ion	a	a-17	a-18	b	b-17	b-18	b+18	c	i	x	y	z	C-Term.	Ion
1	L	**86.096**	69.070	68.086	114.091	97.065	96.081	132.102	131.118	**86.096**	201.098	**175.119**	158.092	12	R
2	T	187.144	170.118	169.134	215.139	198.112	197.128	233.150	232.166	74.060	272.135	246.156	229.130	11	A
3	I	300.228	283.202	282.218	**328.223**	311.197	310.213	346.234	345.250	**86.096**	409.194	**383.215**	**366.188**	10	H
4	L	413.312	396.286	395.302	441.307	424.281	423.297	459.318	458.334	**86.096**	508.263	**482.283**	465.257	9	V
5	H	550.371	533.345	532.361	578.366	561.340	560.355	**596.377**	595.393	**110.071**	623.290	**597.310**	**580.284**	8	D
6	T	651.419	634.392	633.408	679.414	662.387	661.403	697.424	696.440	74.060	737.333	**711.353**	**694.327**	7	N
7	N	765.462	748.435	747.451	793.457	776.430	775.446	**811.467**	810.483	87.055	838.380	**812.401**	**795.374**	6	T
8	D	880.489	863.462	862.478	**908.484**	891.457	890.473	926.494	925.510	88.039	975.439	**949.460**	**932.433**	5	H
9	V	979.557	962.531	961.547	1007.552	990.525	989.541	1025.563	1024.579	72.081	1088.523	1062.544	1045.517	4	L
10	H	1116.616	1099.589	1098.605	1144.611	1127.584	1126.600	1162.621	1161.637	**110.071**	1201.607	1175.628	1158.601	3	I
11	A	1187.653	1170.627	1169.643	1215.648	1198.621	1197.637	1233.659	1232.675	44.049	1302.655	1276.676	1259.649	2	T
12	R	**1343.754**	1326.728	1325.744	1371.749	1354.723	1353.739	**1389.760**	1388.776	129.113	1415.739	**1389.760**	**1372.733**	1	L

Copyright © 1999-2008 Bruker Daltonik GmbH

5`N parentmass 1110.568

Spectrum Analysis Report

Sequence Name:		Parentmass:	1110.568	Mass Error:	0.024	MH+ (mono):	1110.544
MH+ (avg):	1111.193	Threshold (a.i.):	0.000	Tolerance (Da):	0.700	Number of Peaks:	24
Above Threshold:	24	Assigned Peaks:	13	Not assigned Peaks:	11		

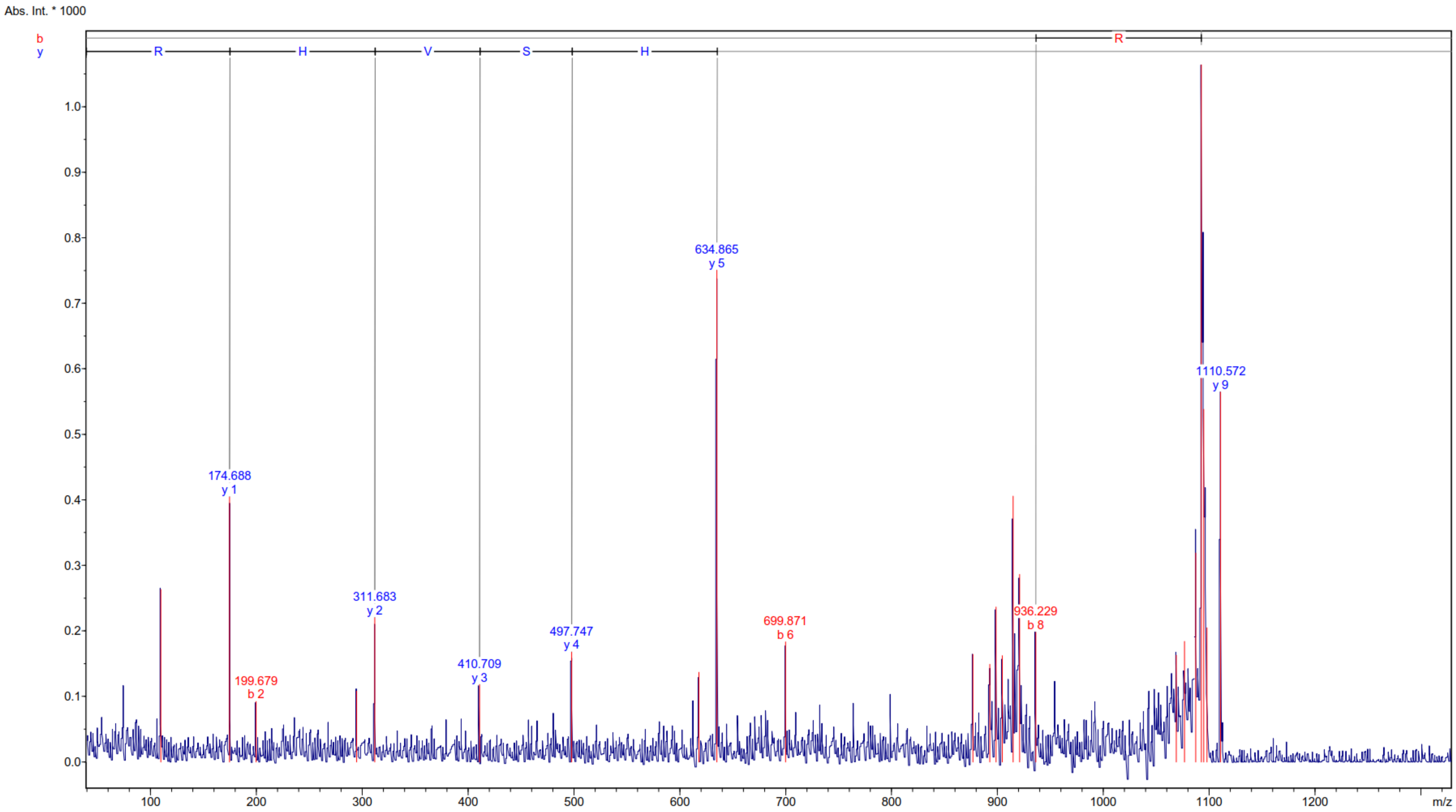

Copyright © 1999-2008 Bruker Daltonik GmbH

Display Parameter:

Parentmass:	1110.568	Mass Error:	0.024	MH+ (mono):	1110.544	MH+ (avg):	1111.193
Threshold (a.i.):	0.000	Tolerance (Da):	0.700	Number of Peaks:	24	Above Threshold:	24
Assigned Peaks:	13	Not assigned Peaks:	11				

Peaklist:

Peak	Mass	Intensity	Peak	Mass	Intensity	Peak	Mass	Intensity	Peak	Mass	Intensity	Peak	Mass	Intensity
1	109.733	263.440	2	174.688	404.901	3	199.679	92.783	4	294.683	108.316	5	311.683	220.689
6	410.709	118.327	7	497.747	168.366	8	617.888	137.200	9	634.865	751.043	10	699.871	183.594
11	876.657	164.425	12	892.665	149.207	13	898.643	236.513	14	904.576	162.422	15	914.721	405.917
16	920.853	286.376	17	936.229	198.534	18	1068.711	163.095	19	1076.547	184.110	20	1087.133	319.020
21	1092.520	1182.906	22	1094.732	538.609	23	1097.700	205.026	24	1110.572	564.973			

Calculated Masses:

QAFEHSVHR

N-Term.	Ion	a	a-17	a-18	b	b-17	b-18	b+18	c	i	x	y	z	C-Term.	Ion
1	Q	101.071	84.044	83.060	129.066	112.039	111.055	147.076	146.092	101.071	201.098	**175.119**	158.092	9	R
2	A	172.108	155.082	154.097	**200.103**	183.076	182.092	218.114	217.130	44.049	338.157	**312.178**	**295.151**	8	H
3	F	319.176	302.150	301.166	347.171	330.145	329.161	365.182	364.198	120.081	437.226	**411.246**	394.220	7	V
4	E	448.219	431.193	430.208	476.214	459.187	458.203	494.225	493.241	102.055	524.258	**498.278**	481.252	6	S
5	H	585.278	568.251	567.267	613.273	596.246	595.262	631.283	630.299	**110.071**	661.316	**635.337**	**618.311**	5	H
6	S	672.310	655.283	654.299	**700.305**	683.278	682.294	718.315	717.331	60.044	790.359	764.380	747.353	4	E
7	V	771.378	754.352	753.368	799.373	782.347	781.363	817.384	816.400	72.081	937.427	911.448	894.422	3	F
8	H	908.437	891.411	890.427	**936.432**	919.406	918.422	954.443	953.459	**110.071**	1008.465	982.485	965.459	2	A
9	R	1064.538	1047.512	1046.528	**1092.533**	1075.507	1074.523	**1110.544**	1109.560	129.113	1136.523	**1110.544**	1093.517	1	Q

Copyright © 1999-2008 Bruker Daltonik GmbH

CRISP parentmass 1168.696

Spectrum Analysis Report

Sequence Name:		Parentmass:	1168.696	Mass Error:	0.172	MH+ (mono):	1168.523
MH+ (avg):	1169.181	Threshold (a.i.):	0.000	Tolerance (Da):	0.700	Number of Peaks:	40
Above Threshold:	40	Assigned Peaks:	27	Not assigned Peaks:	13		

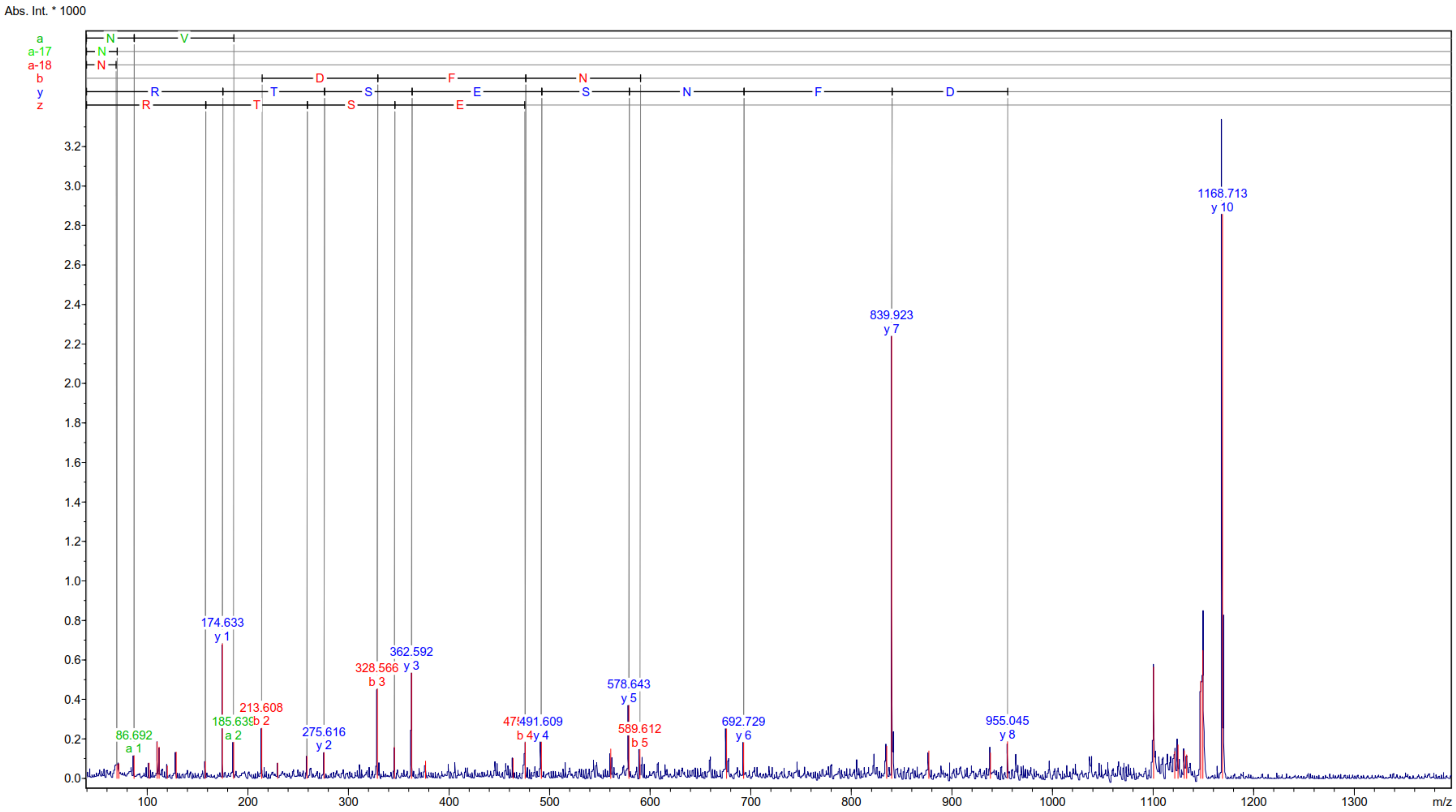

Copyright © 1999-2008 Bruker Daltonik GmbH

Spectrum Analysis Report

Display Parameter:

Parentmass:	1168.696	Mass Error:	0.172	MH+ (mono):	1168.523	MH+ (avg):	1169.181
Threshold (a.i.):	0.000	Tolerance (Da):	0.700	Number of Peaks:	40	Above Threshold:	40
Assigned Peaks:	27	Not assigned Peaks:	13				

Peaklist:

Peak	Mass	Intensity	Peak	Mass	Intensity	Peak	Mass	Intensity	Peak	Mass	Intensity	Peak	Mass	Intensity
1	69.659	70.117	2	71.750	74.967	3	86.692	116.206	4	101.662	78.675	5	109.654	185.420
6	111.701	159.314	7	119.671	64.549	8	128.668	134.153	9	157.627	87.235	10	174.633	683.940
11	185.639	184.329	12	213.608	255.410	13	229.587	77.968	14	258.610	114.569	15	275.616	132.404
16	328.566	454.828	17	345.552	159.834	18	362.592	535.385	19	376.534	88.508	20	463.571	102.459
21	475.614	185.102	22	491.609	185.387	23	560.590	148.923	24	578.643	371.533	25	589.612	148.238
26	675.692	298.153	27	692.729	183.452	28	834.887	163.368	29	839.923	2241.203	30	876.877	139.375
31	937.977	129.813	32	955.045	186.902	33	1100.254	564.225	34	1121.279	136.176	35	1124.453	170.610
36	1130.675	140.668	37	1133.176	120.222	38	1147.104	493.545	39	1149.226	648.364	40	1168.713	2858.462

Calculated Masses:
NVDFNSESTR

N-Term.	Ion	a	a-17	a-18	b	b-17	b-18	b+18	c	i	x	y	z	C-Term.	Ion
1	N	**87.055**	**70.029**	**69.045**	115.050	98.024	97.040	133.061	132.077	**87.055**	201.098	**175.119**	**158.092**	10	R
2	V	**186.124**	169.097	168.113	**214.119**	197.092	196.108	232.129	231.145	**72.081**	302.146	**276.167**	**259.140**	9	T
3	D	301.151	284.124	283.140	**329.146**	312.119	311.135	347.156	**346.172**	88.039	389.178	**363.199**	**346.172**	8	S
4	F	448.219	431.193	430.208	**476.214**	459.187	458.203	494.225	493.241	**120.081**	518.221	**492.241**	**475.215**	7	E
5	N	562.262	545.235	544.251	**590.257**	573.230	572.246	608.267	607.283	**87.055**	605.253	**579.273**	562.247	6	S
6	S	649.294	632.267	631.283	677.289	660.262	659.278	695.299	694.315	60.044	719.295	**693.316**	**676.290**	5	N
7	E	778.337	761.310	760.326	806.332	789.305	788.321	824.342	823.358	**102.055**	866.364	**840.385**	823.358	4	F
8	S	865.369	848.342	847.358	893.364	**876.337**	875.353	911.374	910.390	60.044	981.391	**955.412**	**938.385**	3	D
9	T	966.416	949.390	948.406	994.411	977.385	976.401	1012.422	1011.438	74.060	1080.459	1054.480	1037.453	2	V
10	R	1122.517	1105.491	1104.507	1150.512	**1133.486**	**1132.502**	**1168.523**	1167.539	**129.113**	1194.502	**1168.523**	1151.496	1	N

Copyright © 1999-2008 Bruker Daltonik GmbH

CRISP parentmass 1195.609

Spectrum Analysis Report

Sequence Name:		Parentmass:	1195.609	Mass Error:	-0.035	MH+ (mono):	1195.644
MH+ (avg):	1196.336	Threshold (a.i.):	0.000	Tolerance (Da):	0.700	Number of Peaks:	39
Above Threshold:	39	Assigned Peaks:	31	Not assigned Peaks:	8		

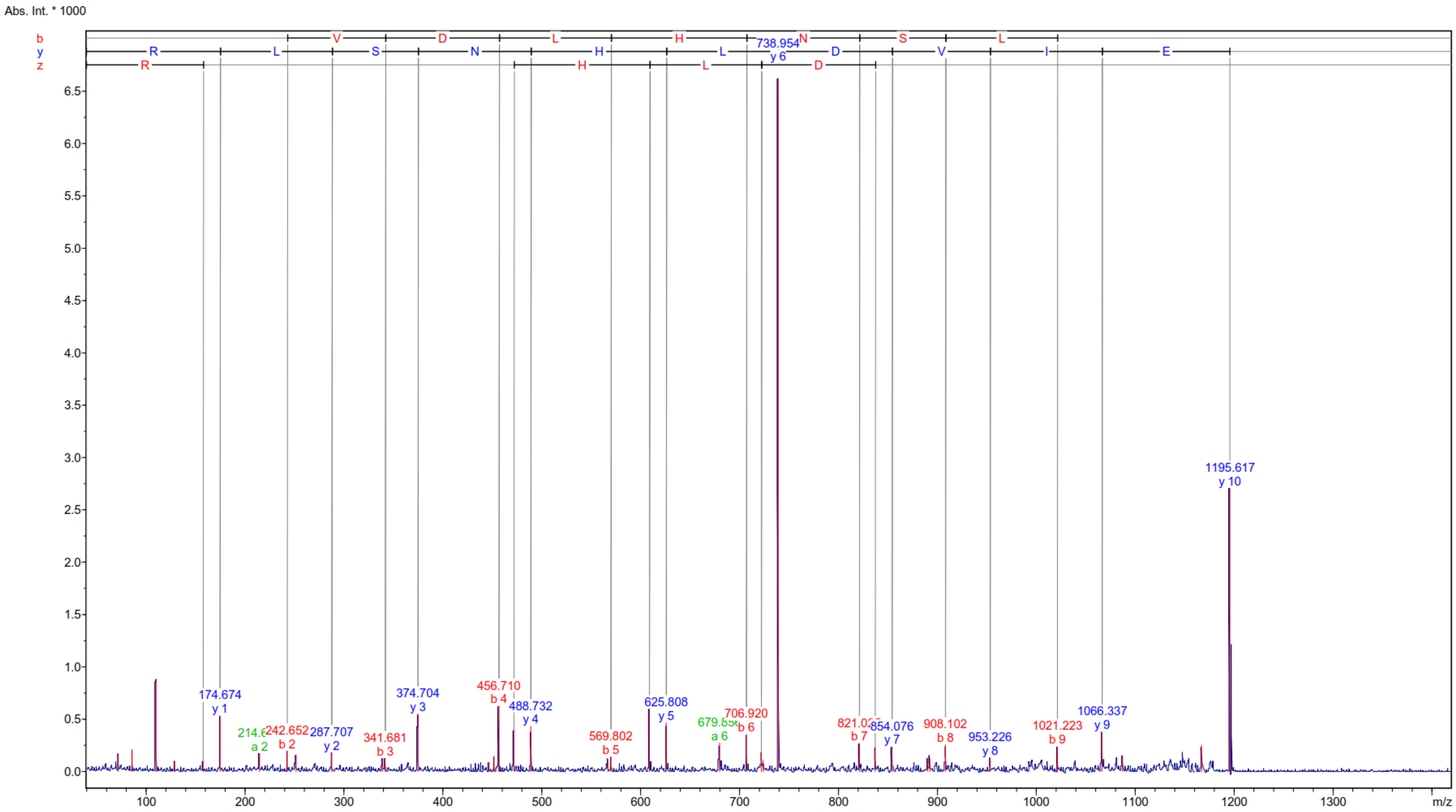

Copyright © 1999-2008 Bruker Daltonik GmbH

Spectrum Analysis Report

Display Parameter:

Parentmass:	1195.609	Mass Error:	-0.035	MH+ (mono):	1195.644	MH+ (avg):	1196.336
Threshold (a.i.):	0.000	Tolerance (Da):	0.700	Number of Peaks:	39	Above Threshold:	39
Assigned Peaks:	31	Not assigned Peaks:	8				

Peaklist:

Peak	Mass	Intensity	Peak	Mass	Intensity	Peak	Mass	Intensity	Peak	Mass	Intensity	Peak	Mass	Intensity
1	71.774	163.124	2	85.767	209.551	3	109.710	882.222	4	128.719	99.408	5	157.665	96.480
6	174.674	532.781	7	214.649	174.915	8	242.652	197.926	9	251.635	158.944	10	287.707	183.626
11	338.637	135.096	12	341.681	127.046	13	374.704	551.107	14	446.685	89.170	15	451.708	146.577
16	456.710	625.885	17	471.704	408.133	18	488.732	433.293	19	566.762	115.497	20	569.802	141.802
21	608.807	679.907	22	625.808	467.170	23	679.856	276.576	24	706.920	355.290	25	721.964	182.172
26	723.717	109.390	27	738.954	6764.837	28	821.025	269.550	29	837.026	229.429	30	854.076	237.628
31	890.082	125.453	32	892.254	127.576	33	908.102	257.531	34	953.226	132.610	35	1021.223	239.794
36	1066.337	377.855	37	1087.082	155.203	38	1166.924	250.794	39	1195.617	2707.766			

Calculated Masses:
EIVDLHNSLR

N-Term.	Ion	a	a-17	a-18	b	b-17	b-18	b+18	c	i	x	y	z	C-Term.	Ion
1	E	102.055	85.028	84.044	130.050	113.023	112.039	148.060	147.076	102.055	201.098	**175.119**	**158.092**	10	R
2	I	**215.139**	198.112	197.128	**243.134**	226.107	225.123	261.144	260.160	**86.096**	314.182	**288.203**	271.176	9	L
3	V	314.207	297.181	296.197	**342.202**	325.176	324.192	360.213	359.229	**72.081**	401.214	**375.235**	358.208	8	S
4	D	429.234	412.208	411.224	**457.229**	440.203	439.219	475.240	474.256	88.039	515.257	**489.278**	**472.251**	7	N
5	L	542.318	525.292	524.308	**570.313**	553.287	552.303	588.324	587.340	**86.096**	652.316	**626.337**	**609.310**	6	H
6	H	**679.377**	662.351	661.367	**707.372**	690.346	689.362	725.383	**724.399**	**110.071**	765.400	**739.421**	**722.394**	5	L
7	N	793.420	776.394	775.410	**821.415**	804.389	803.405	839.426	838.442	87.055	880.427	**854.448**	**837.421**	4	D
8	S	880.452	863.426	862.442	**908.447**	891.421	**890.437**	926.458	925.474	60.044	979.496	**953.516**	936.490	3	V
9	L	993.536	976.510	975.526	**1021.531**	1004.505	1003.521	1039.542	1038.558	**86.096**	1092.580	**1066.600**	1049.574	2	I
10	R	1149.637	1132.611	1131.627	1177.632	1160.606	1159.622	**1195.643**	1194.659	**129.113**	1221.622	**1195.643**	1178.616	1	E

Copyright © 1999-2008 Bruker Daltonik GmbH

CRISP parentmass 1553.910

Spectrum Analysis Report

Sequence Name:		Parentmass:	1553.910	Mass Error:	0.241	MH+ (mono):	1553.669
MH+ (avg):	1554.663	Threshold (a.i.):	0.000	Tolerance (Da):	0.700	Number of Peaks:	67
Above Threshold:	67	Assigned Peaks:	34	Not assigned Peaks:	33		

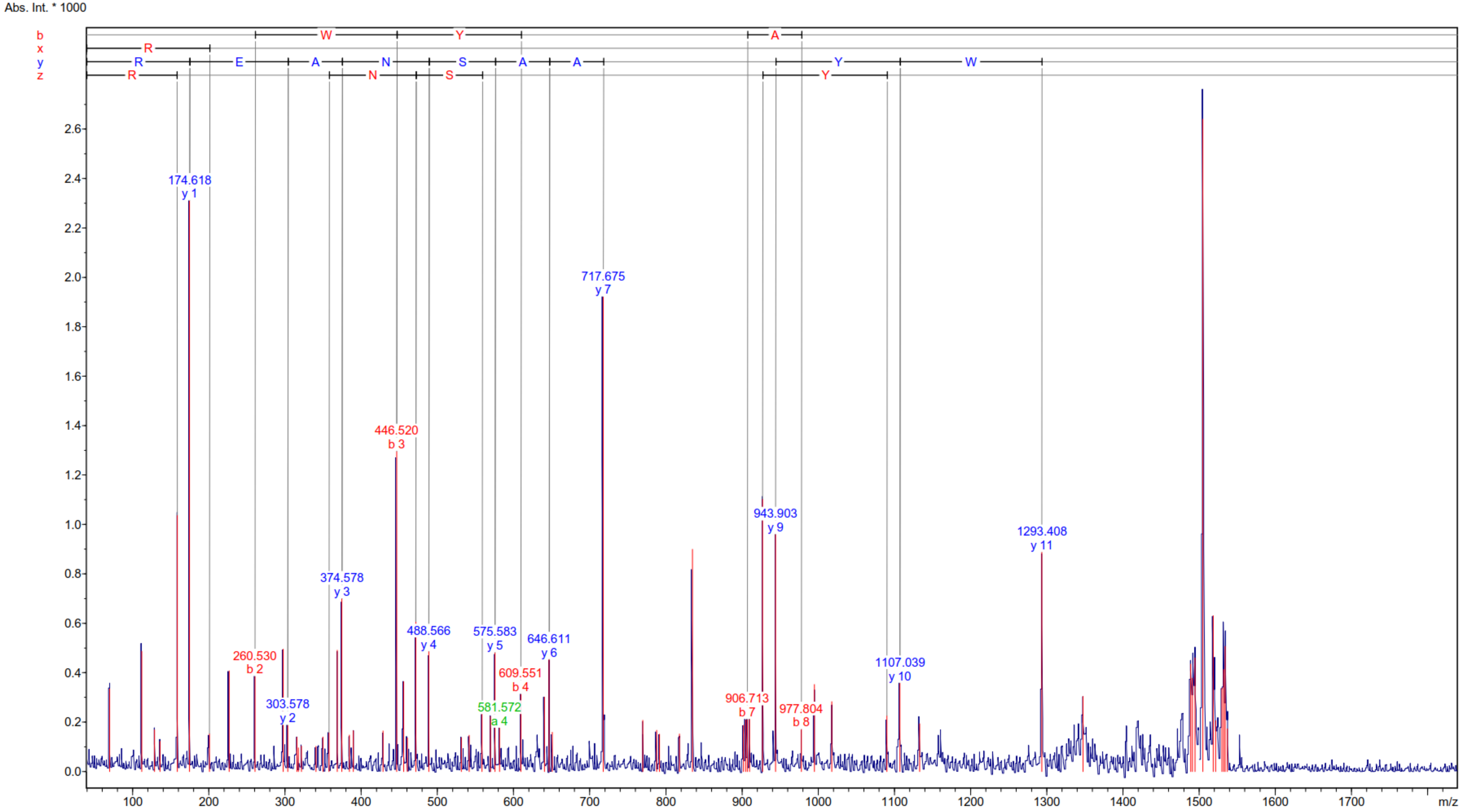

Copyright © 1999-2008 Bruker Daltonik GmbH

Spectrum Analysis Report

Display Parameter:

Parentmass:	1553.910	Mass Error:	0.241	MH+ (mono):	1553.669	MH+ (avg):	1554.663
Threshold (a.i.):	0.000	Tolerance (Da):	0.700	Number of Peaks:	67	Above Threshold:	67
Assigned Peaks:	34	Not assigned Peaks:	33				

Peaklist:

Peak	Mass	Intensity	Peak	Mass	Intensity	Peak	Mass	Intensity	Peak	Mass	Intensity	Peak	Mass	Intensity
1	69.702	335.297	2	111.668	486.549	3	128.649	168.849	4	135.639	122.837	5	158.611	1037.570
6	174.618	2311.164	7	200.576	153.486	8	226.552	407.711	9	260.530	385.479	10	297.548	495.571
11	303.578	187.875	12	315.540	139.046	13	317.491	96.029	14	321.572	103.094	15	340.554	102.111
16	349.525	139.981	17	357.545	157.411	18	368.566	491.579	19	374.578	700.442	20	384.527	147.642
21	389.559	165.525	22	428.547	164.283	23	446.520	1296.940	24	455.550	363.777	25	460.512	136.654
26	471.556	607.480	27	488.566	486.829	28	531.545	137.817	29	541.550	146.947	30	558.600	242.603
31	569.583	223.660	32	575.583	482.349	33	581.572	176.798	34	609.551	313.638	35	640.627	301.970
36	646.611	454.667	37	650.638	159.795	38	717.675	1922.302	39	769.688	207.413	40	787.712	167.169
41	791.598	151.734	42	817.737	152.606	43	834.735	900.071	44	901.791	187.610	45	904.656	209.423
46	906.713	211.662	47	909.793	214.469	48	926.860	1102.507	49	943.903	960.531	50	977.804	171.536
51	994.820	352.601	52	1017.770	282.936	53	1090.066	226.329	54	1107.039	358.748	55	1133.028	193.496
56	1293.408	888.326	57	1347.429	304.728	58	1488.683	435.126	59	1491.130	378.001	60	1494.435	460.441
61	1504.881	2640.884	62	1518.366	630.611	63	1521.608	207.048	64	1529.847	338.073	65	1531.990	413.404
66	1534.226	506.873	67	1537.055	173.315									

Calculated Masses:
MEWYPEAASNAER

N-Term.	Ion	a	a-17	a-18	b	b-17	b-18	b+18	c	i	x	y	z	C-Term.	Ion
1	M	104.053	87.026	86.042	132.048	115.021	114.037	150.058	149.074	104.053	**201.098**	**175.119**	**158.092**	13	R
2	E	233.095	216.069	215.085	**261.090**	244.064	243.080	279.101	278.117	102.055	330.141	**304.162**	287.135	12	E
3	W	419.175	402.148	401.164	**447.170**	430.143	**429.159**	465.180	464.196	**159.092**	401.178	**375.199**	**358.172**	11	A
4	Y	**582.238**	565.212	564.228	**610.233**	593.206	592.222	628.244	627.260	**136.076**	515.221	**489.242**	**472.215**	10	N
5	P	679.291	662.264	661.280	707.286	690.259	689.275	725.296	724.312	**70.065**	602.253	**576.274**	**559.247**	9	S
6	E	808.333	**791.307**	790.323	836.328	819.302	**818.318**	854.339	853.355	102.055	673.290	**647.311**	630.284	8	A
7	A	879.371	862.344	861.360	**907.365**	890.339	889.355	925.376	924.392	44.049	744.327	**718.348**	701.321	7	A
8	A	950.408	933.381	932.397	**978.403**	961.376	960.392	996.413	**995.429**	44.049	873.370	847.390	830.364	6	E
9	S	1037.440	1020.413	1019.429	1065.435	1048.408	1047.424	1083.445	1082.461	60.044	970.422	**944.443**	**927.417**	5	P
10	N	1151.483	1134.456	**1133.472**	1179.478	1162.451	1161.467	1197.488	1196.504	87.055	**1133.486**	**1107.507**	**1090.480**	4	Y
11	A	1222.520	1205.493	1204.509	1250.515	1233.488	1232.504	1268.525	1267.541	44.049	1319.565	**1293.586**	1276.559	3	W
12	E	1351.562	1334.536	1333.552	1379.557	1362.531	1361.547	1397.568	1396.584	102.055	1448.608	1422.628	1405.602	2	E
13	R	1507.663	**1490.637**	1489.653	1535.658	**1518.632**	1517.648	1553.669	1552.685	**129.113**	1579.648	1553.669	**1536.642**	1	M

Copyright © 1999-2008 Bruker Daltonik GmbH

CVF parentmass 1306.709

Spectrum Analysis Report

Sequence Name:		Parentmass:	1306.709	Mass Error:	0.062	MH+ (mono):	1306.647
MH+ (avg):	1307.499	Threshold (a.i.):	0.000	Tolerance (Da):	0.700	Number of Peaks:	54
Above Threshold:	54	Assigned Peaks:	28	Not assigned Peaks:	26		

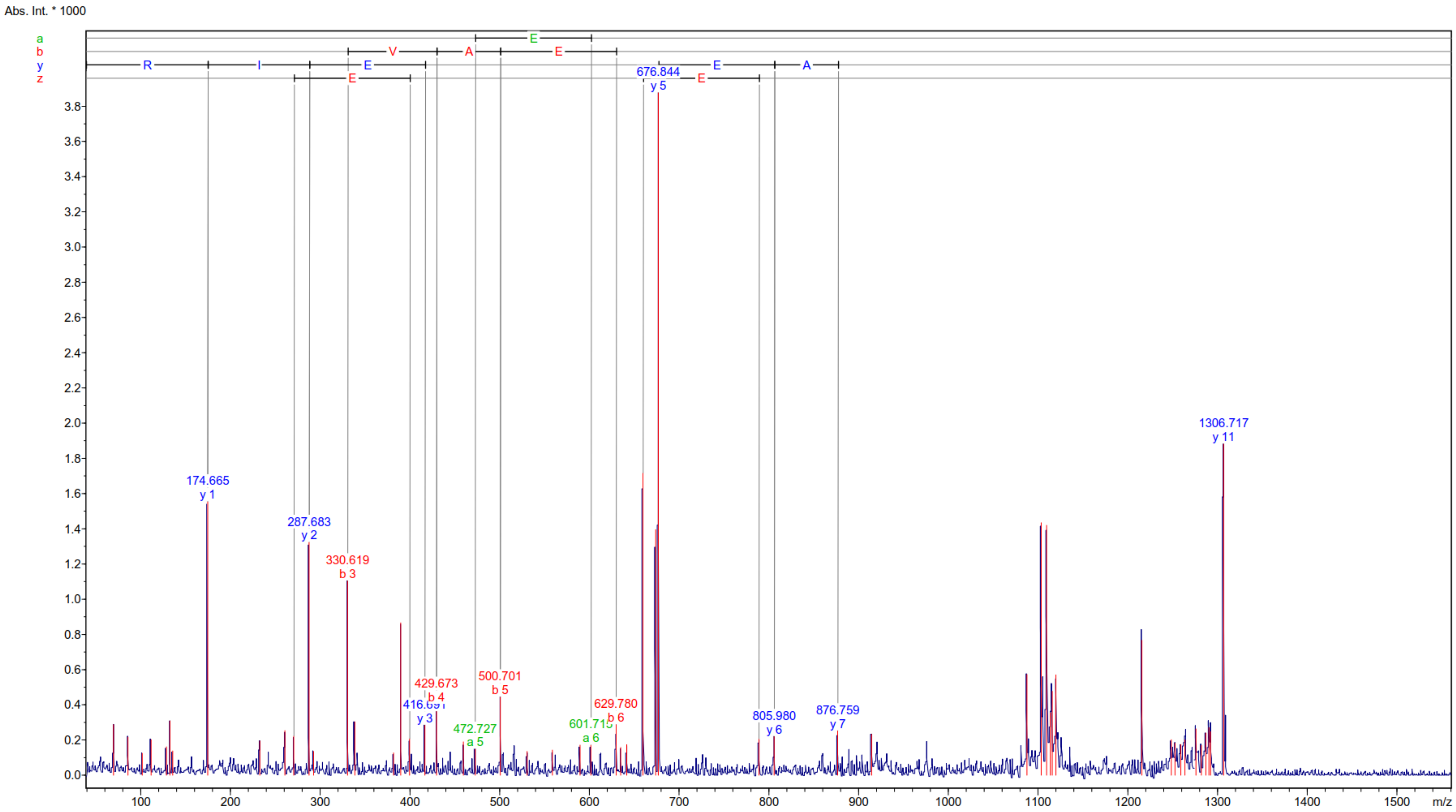

Copyright © 1999-2008 Bruker Daltonik GmbH

Spectrum Analysis Report

Display Parameter:

Parentmass:	1306.709	Mass Error:	0.062	MH+ (mono):	1306.647	MH+ (avg):	1307.499
Threshold (a.i.):	0.000	Tolerance (Da):	0.700	Number of Peaks:	54	Above Threshold:	54
Assigned Peaks:	28	Not assigned Peaks:	26				

Peaklist:

Peak	Mass	Intensity	Peak	Mass	Intensity	Peak	Mass	Intensity	Peak	Mass	Intensity	Peak	Mass	Intensity
1	69.757	287.777	2	85.737	216.113	3	101.658	125.057	4	111.711	202.205	5	128.720	160.935
6	132.642	308.162	7	135.696	138.337	8	174.665	1555.374	9	232.634	194.388	10	260.628	253.219
11	270.656	221.666	12	287.683	1323.974	13	292.604	136.239	14	330.619	1105.275	15	338.685	303.891
16	381.699	126.875	17	389.644	865.618	18	399.671	206.850	19	416.691	285.839	20	429.673	405.409
21	459.645	189.185	22	472.727	149.918	23	500.701	446.332	24	530.728	135.349	25	558.834	143.466
26	589.748	171.260	27	601.715	172.331	28	629.780	290.093	29	634.877	157.642	30	641.768	173.500
31	659.812	1717.158	32	673.882	1395.395	33	676.844	3878.330	34	788.937	207.291	35	805.980	221.756
36	876.759	253.958	37	914.462	233.214	38	1087.582	572.567	39	1103.530	1434.413	40	1109.639	1419.645
41	1113.751	354.398	42	1115.814	477.460	43	1120.018	570.742	44	1215.631	766.735	45	1248.473	202.000
46	1252.513	181.848	47	1259.156	175.384	48	1263.668	200.316	49	1275.971	263.884	50	1281.928	169.473
51	1287.112	239.427	52	1290.319	270.243	53	1292.209	247.257	54	1306.717	1882.377			

Calculated Masses:

GICVAEPYEIR 3: Carbamidomethyl (C)

N-Term.	Ion	a	a-17	a-18	b	b-17	b-18	b+18	c	i	x	y	z	C-Term.	Ion
1	G	30.034	13.007	12.023	58.029	41.002	40.018	76.039	75.055	30.034	201.098	**175.119**	158.092	11	R
2	I	143.118	126.091	125.107	171.113	154.086	153.102	189.123	188.139	**86.096**	314.182	**288.203**	**271.176**	10	I
3	C*	303.149	286.122	285.138	**331.143**	314.117	313.133	349.154	348.170	**133.043**	443.225	**417.246**	**400.219**	9	E
4	V	402.217	385.190	384.206	**430.212**	413.185	412.201	448.222	447.238	72.081	606.288	580.309	563.282	8	Y
5	A	**473.254**	456.228	455.243	**501.249**	484.222	483.238	519.260	518.276	44.049	703.341	**677.362**	**660.335**	7	P
6	E	**602.297**	585.270	584.286	**630.292**	613.265	612.281	648.302	647.318	**102.055**	832.384	**806.404**	**789.378**	6	E
7	P	699.349	682.323	681.339	727.344	710.318	709.334	745.355	744.371	**70.065**	903.421	**877.441**	860.415	5	A
8	Y	862.413	845.386	844.402	890.408	873.381	872.397	908.418	907.434	**136.076**	1002.489	976.510	959.483	4	V
9	E	991.455	974.429	973.445	1019.450	1002.424	1001.440	1037.461	1036.477	**102.055**	1162.520	1136.540	**1119.514**	3	C*
10	I	1104.539	**1087.513**	1086.529	1132.534	**1115.508**	1114.524	1150.545	1149.561	**86.096**	**1275.604**	1249.625	1232.598	2	I
11	R	1260.641	1243.614	1242.630	1288.635	1271.609	1270.625	**1306.646**	1305.662	**129.113**	1332.625	**1306.646**	**1289.619**	1	G

Copyright © 1999-2008 Bruker Daltonik GmbH

CVF parentmass 1337.885

Spectrum Analysis Report

Sequence Name:		Parentmass:	1337.885	Mass Error:	0.236	MH+ (mono):	1337.649
MH+ (avg):	1338.447	Threshold (a.i.):	0.000	Tolerance (Da):	0.700	Number of Peaks:	73
Above Threshold:	73	Assigned Peaks:	38	Not assigned Peaks:	35		

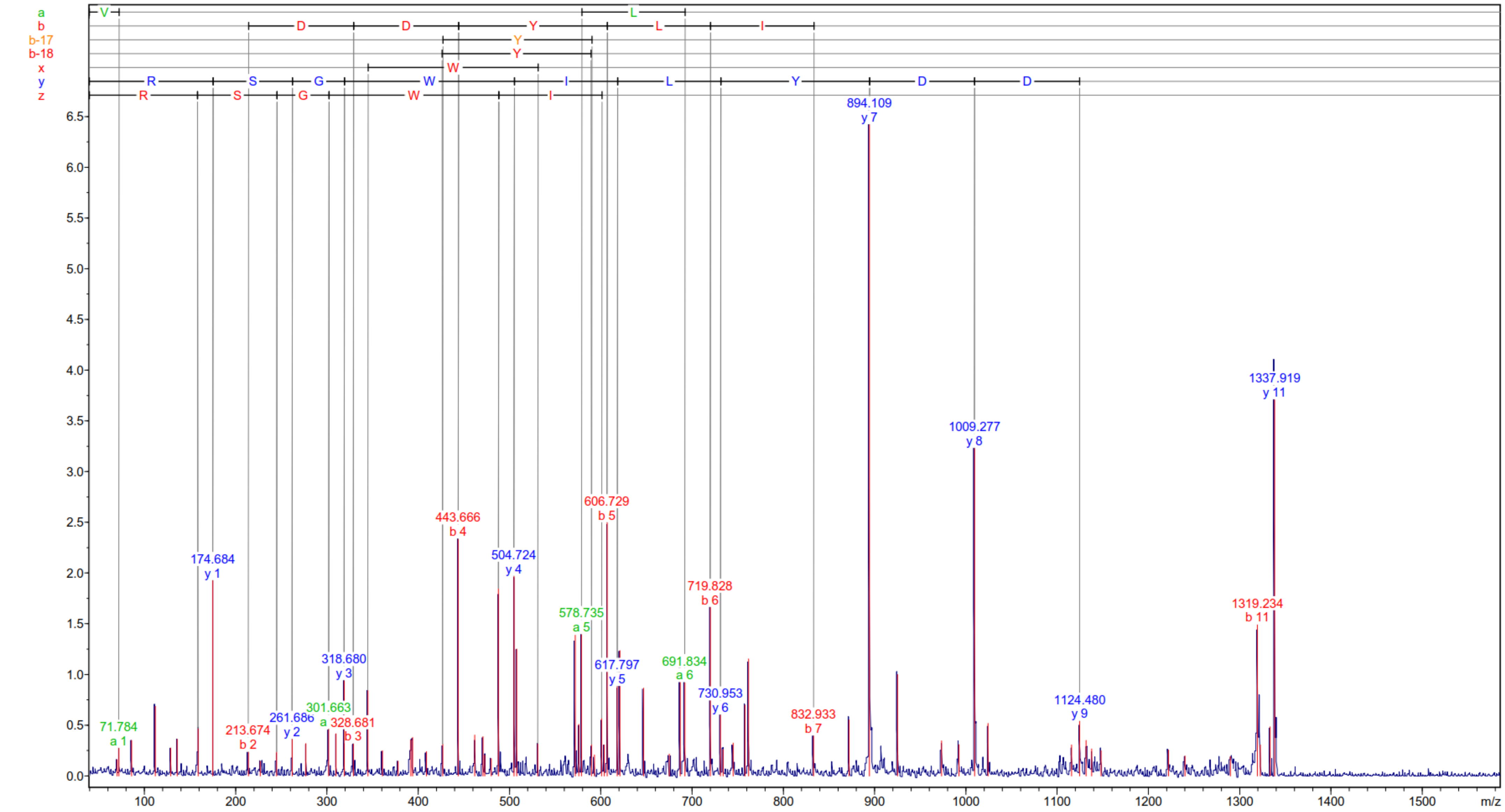

Copyright © 1999-2008 Bruker Daltonik GmbH

Spectrum Analysis Report

Display Parameter:

Parentmass:	1337.885	Mass Error:	0.236	MH+ (mono):	1337.649	MH+ (avg):	1338.447
Threshold (a.i.):	0.000	Tolerance (Da):	0.700	Number of Peaks:	73	Above Threshold:	73
Assigned Peaks:	38	Not assigned Peaks:	35				

Peaklist:

Peak	Mass	Intensity	Peak	Mass	Intensity	Peak	Mass	Intensity	Peak	Mass	Intensity	Peak	Mass	Intensity
1	69.764	160.824	2	71.784	276.462	3	85.766	351.548	4	111.719	688.551	5	128.713	275.981
6	135.681	361.804	7	158.674	469.052	8	174.684	1928.457	9	213.674	237.133	10	226.715	146.800
11	244.687	238.448	12	261.686	364.212	13	276.688	320.419	14	301.663	462.933	15	309.693	416.788
16	318.680	942.011	17	328.681	318.597	18	344.627	844.128	19	360.627	250.975	20	377.651	146.047
21	391.678	354.628	22	393.650	380.336	23	408.705	241.460	24	426.646	312.853	25	443.666	2339.231
26	461.653	403.668	27	470.708	388.833	28	472.720	206.251	29	479.698	173.258	30	487.701	1846.968
31	504.724	1971.230	32	507.661	1250.713	33	530.675	317.182	34	571.769	1388.735	35	575.733	509.761
36	578.735	1397.262	37	589.708	312.910	38	592.737	208.423	39	600.761	568.927	40	603.710	307.778
41	606.729	2499.765	42	617.797	884.954	43	620.732	1237.165	44	646.698	865.970	45	674.806	217.057
46	686.796	1101.252	47	691.834	922.095	48	719.828	1663.989	49	730.953	604.470	50	733.799	261.022
51	744.779	325.205	52	757.865	701.665	53	761.768	1155.767	54	832.933	397.320	55	871.941	553.332
56	894.109	6423.685	57	924.904	1000.550	58	973.033	345.723	59	992.222	308.447	60	1009.277	3233.435
61	1023.991	518.685	62	1115.472	306.620	63	1124.480	542.089	64	1131.593	351.961	65	1137.606	267.320
66	1147.951	249.415	67	1221.699	256.272	68	1239.782	196.824	69	1289.530	193.108	70	1319.234	1492.658
71	1322.359	306.328	72	1333.248	484.871	73	1337.919	3711.113						

Calculated Masses:
VNDDYLIWGSR

N-Term.	Ion	a	a-17	a-18	b	b-17	b-18	b+18	c	i	x	y	z	C-Term.	Ion
1	V	**72.081**	55.054	54.070	100.076	83.049	82.065	118.086	117.102	**72.081**	201.098	**175.119**	**158.092**	11	R
2	N	186.124	169.097	168.113	**214.119**	197.092	196.108	232.129	231.145	87.055	288.130	**262.151**	**245.124**	10	S
3	D	**301.151**	284.124	283.140	**329.146**	312.119	311.135	347.156	346.172	88.039	**345.152**	**319.172**	**302.146**	9	G
4	D	416.178	399.151	398.167	**444.173**	**427.146**	**426.162**	**462.183**	**461.199**	88.039	**531.231**	**505.252**	**488.225**	8	W
5	Y	**579.241**	562.214	561.230	**607.236**	**590.209**	**589.225**	625.246	624.262	**136.076**	644.315	**618.336**	**601.309**	7	I
6	L	**692.325**	**675.298**	**674.314**	**720.320**	703.293	702.309	738.330	737.346	**86.096**	**757.399**	**731.420**	714.393	6	L
7	I	805.409	788.382	787.398	**833.404**	816.377	815.393	851.415	850.431	**86.096**	920.462	**894.483**	877.457	5	Y
8	W	991.488	974.462	**973.478**	1019.483	1002.457	1001.473	1037.494	1036.510	**159.092**	1035.489	**1009.510**	**992.484**	4	D
9	G	1048.510	1031.483	1030.499	1076.505	1059.478	1058.494	1094.515	1093.531	30.034	1150.516	**1124.537**	1107.511	3	D
10	S	1135.542	1118.515	1117.531	1163.537	1146.510	1145.526	1181.547	1180.563	60.044	1264.559	1238.580	**1221.553**	2	N
11	R	1291.643	1274.616	1273.632	**1319.638**	1302.611	1301.627	**1337.648**	1336.664	**129.113**	1363.628	**1337.648**	1320.622	1	V

Copyright © 1999-2008 Bruker Daltonik GmbH

PLA$_2$ parentmass 1769.783

Spectrum Analysis Report

Sequence Name:		Parentmass:	1769.783	Mass Error:	0.115	MH+ (mono):	1769.668
MH+ (avg):	1770.905	Threshold (a.i.):	0.000	Tolerance (Da):	0.700	Number of Peaks:	83
Above Threshold:	83	Assigned Peaks:	52	Not assigned Peaks:	31		

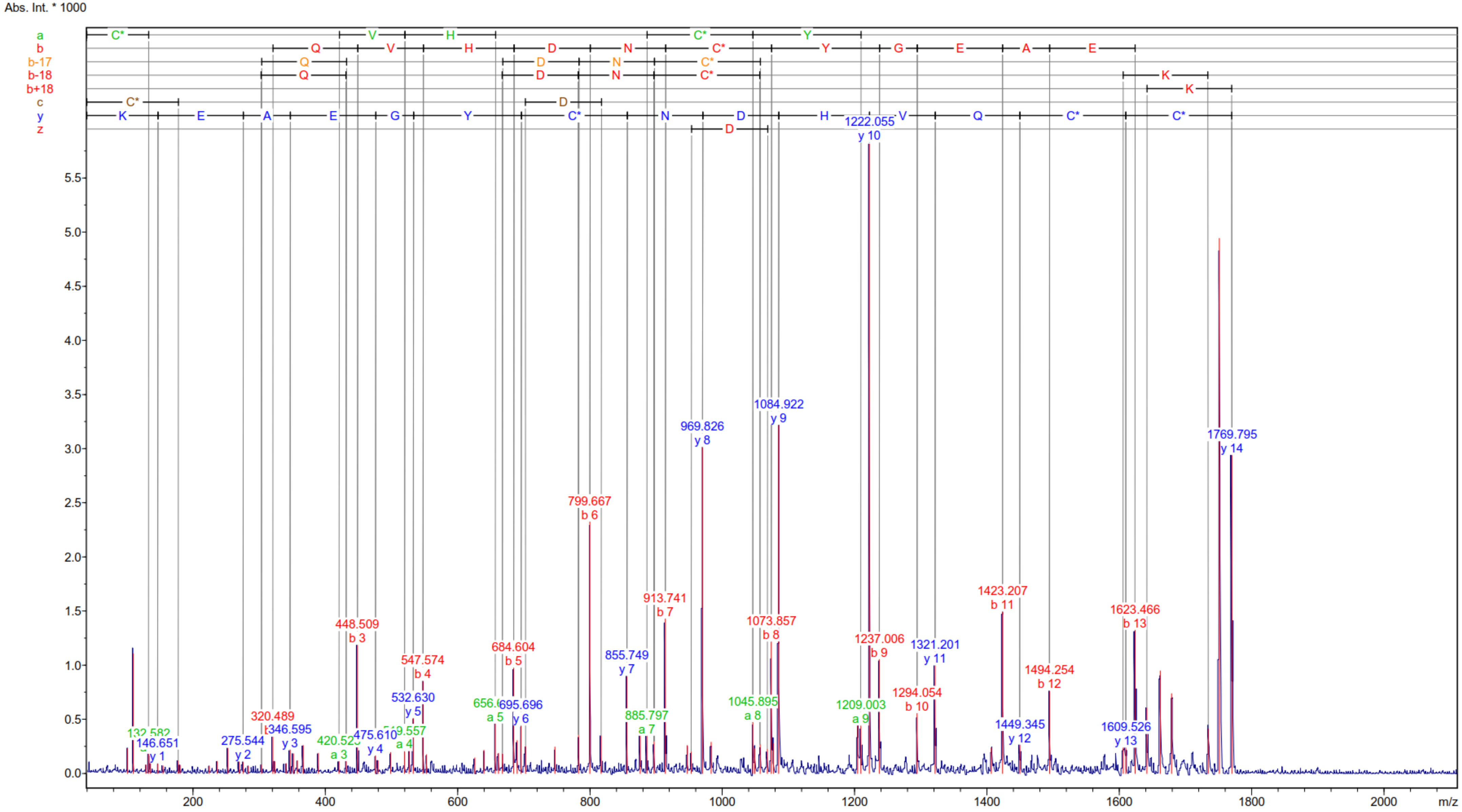

Copyright © 1999-2008 Bruker Daltonik GmbH

Spectrum Analysis Report

Display Parameter:

Parentmass:	1769.783	Mass Error:	0.115	MH+ (mono):	1769.668	MH+ (avg):	1770.905
Threshold (a.i.):	0.000	Tolerance (Da):	0.700	Number of Peaks:	83	Above Threshold:	83
Assigned Peaks:	52	Not assigned Peaks:	31				

Peaklist:

Peak	Mass	Intensity	Peak	Mass	Intensity	Peak	Mass	Intensity	Peak	Mass	Intensity	Peak	Mass	Intensity
1	100.681	232.468	2	109.656	1106.932	3	128.642	81.998	4	132.582	180.801	5	135.631	160.462
6	146.651	92.332	7	154.558	70.854	8	177.564	118.912	9	180.592	77.091	10	224.513	69.341
11	236.581	113.322	12	252.530	243.712	13	269.547	136.192	14	275.544	108.761	15	283.565	68.378
16	303.461	83.065	17	320.489	338.996	18	323.524	111.259	19	340.496	93.539	20	346.595	219.136
21	351.588	184.065	22	357.534	118.762	23	366.533	260.188	24	389.499	187.833	25	420.523	112.422
26	431.486	212.358	27	448.509	1187.173	28	475.610	164.562	29	479.567	120.232	30	498.565	195.233
31	519.557	204.342	32	526.540	456.405	33	532.630	511.762	34	547.574	853.874	35	552.570	175.247
36	625.635	146.286	37	639.563	216.157	38	656.621	459.550	39	661.663	187.176	40	667.595	183.836
41	684.604	976.520	42	689.611	305.177	43	695.696	441.188	44	702.650	251.921	45	746.638	244.704
46	782.683	358.484	47	799.667	2325.365	48	816.671	346.808	49	855.749	899.765	50	875.728	407.836
51	885.797	347.419	52	896.752	297.926	53	913.741	1429.767	54	946.793	260.340	55	952.805	201.734
56	969.826	3015.791	57	982.814	289.596	58	1045.895	473.959	59	1048.774	230.208	60	1056.818	269.611
61	1067.851	223.229	62	1073.857	1217.148	63	1076.852	278.326	64	1084.922	3219.069	65	1204.040	458.336
66	1209.003	441.341	67	1222.055	5827.663	68	1237.006	1055.519	69	1294.054	555.563	70	1321.201	996.642
71	1406.080	238.533	72	1423.207	1495.012	73	1449.345	265.680	74	1494.254	764.784	75	1605.431	252.666
76	1609.526	237.753	77	1623.466	1324.711	78	1641.434	606.253	79	1661.453	948.492	80	1678.618	737.853
81	1734.114	414.767	82	1750.930	4943.190	83	1769.795	2941.363						

Calculated Masses:

CCQVHDNCYGEAEK 1: Carbamidomethyl (C) 2: Carbamidomethyl (C) 8: Carbamidomethyl (C)

N-Term.	Ion	a	a-17	a-18	b	b-17	b-18	b+18	c	i	x	y	z	C-Term.	Ion
1	C*	**133.043**	116.016	115.032	161.038	144.011	143.027	179.048	**178.064**	**133.043**	173.092	**147.113**	130.086	14	K
2	C*	293.074	**276.047**	**275.063**	**321.069**	**304.042**	**303.058**	339.079	338.095	**133.043**	302.135	**276.155**	259.129	13	E
3	Q	**421.132**	404.106	403.122	**449.127**	**432.101**	**431.117**	467.138	466.154	**101.071**	373.172	**347.193**	330.166	12	A
4	V	**520.201**	503.174	502.190	**548.196**	531.169	530.185	566.206	565.222	72.081	502.214	**476.235**	459.209	11	E
5	H	**657.260**	**640.233**	**639.249**	**685.254**	**668.228**	**667.244**	**703.265**	**702.281**	**110.071**	559.236	**533.257**	516.230	10	G
6	D	772.287	755.260	754.276	**800.281**	**783.255**	**782.271**	818.292	**817.308**	88.039	722.299	**696.320**	679.293	9	Y
7	N	**886.329**	869.303	868.319	**914.324**	**897.298**	**896.314**	932.335	931.351	87.055	882.330	**856.351**	839.324	8	C*
8	C*	**1046.360**	1029.334	1028.350	**1074.355**	**1057.328**	**1056.344**	1092.366	1091.382	**133.043**	996.373	**970.393**	**953.367**	7	N
9	Y	**1209.423**	1192.397	1191.413	**1237.418**	1220.392	1219.408	1255.429	1254.445	**136.076**	1111.400	**1085.420**	**1068.394**	6	D
10	G	1266.445	1249.418	1248.434	**1294.440**	1277.413	1276.429	1312.450	1311.466	30.034	1248.459	**1222.479**	1205.453	5	H
11	E	1395.487	1378.461	1377.477	**1423.482**	**1406.456**	**1405.472**	1441.493	1440.509	102.055	1347.527	**1321.548**	1304.521	4	V
12	A	1466.525	**1449.498**	1448.514	**1494.519**	1477.493	1476.509	1512.530	1511.546	44.049	1475.586	**1449.606**	1432.580	3	Q
13	E	1595.567	1578.541	1577.557	**1623.562**	1606.536	**1605.552**	**1641.573**	1640.589	102.055	1635.616	**1609.637**	1592.610	2	C*
14	K	1723.662	1706.636	1705.652	1751.657	**1734.630**	**1733.646**	**1769.668**	1768.684	**101.107**	1795.647	**1769.668**	1752.641	1	C*

Copyright © 1999-2008 Bruker Daltonik GmbH

PLA$_2$ parentmass 987.215

Spectrum Analysis Report

Sequence Name:		Parentmass:	987.512	Mass Error:	0.037	MH+ (mono):	987.475
MH+ (avg):	988.032	Threshold (a.i.):	0.000	Tolerance (Da):	0.700	Number of Peaks:	39
Above Threshold:	39	Assigned Peaks:	22	Not assigned Peaks:	17		

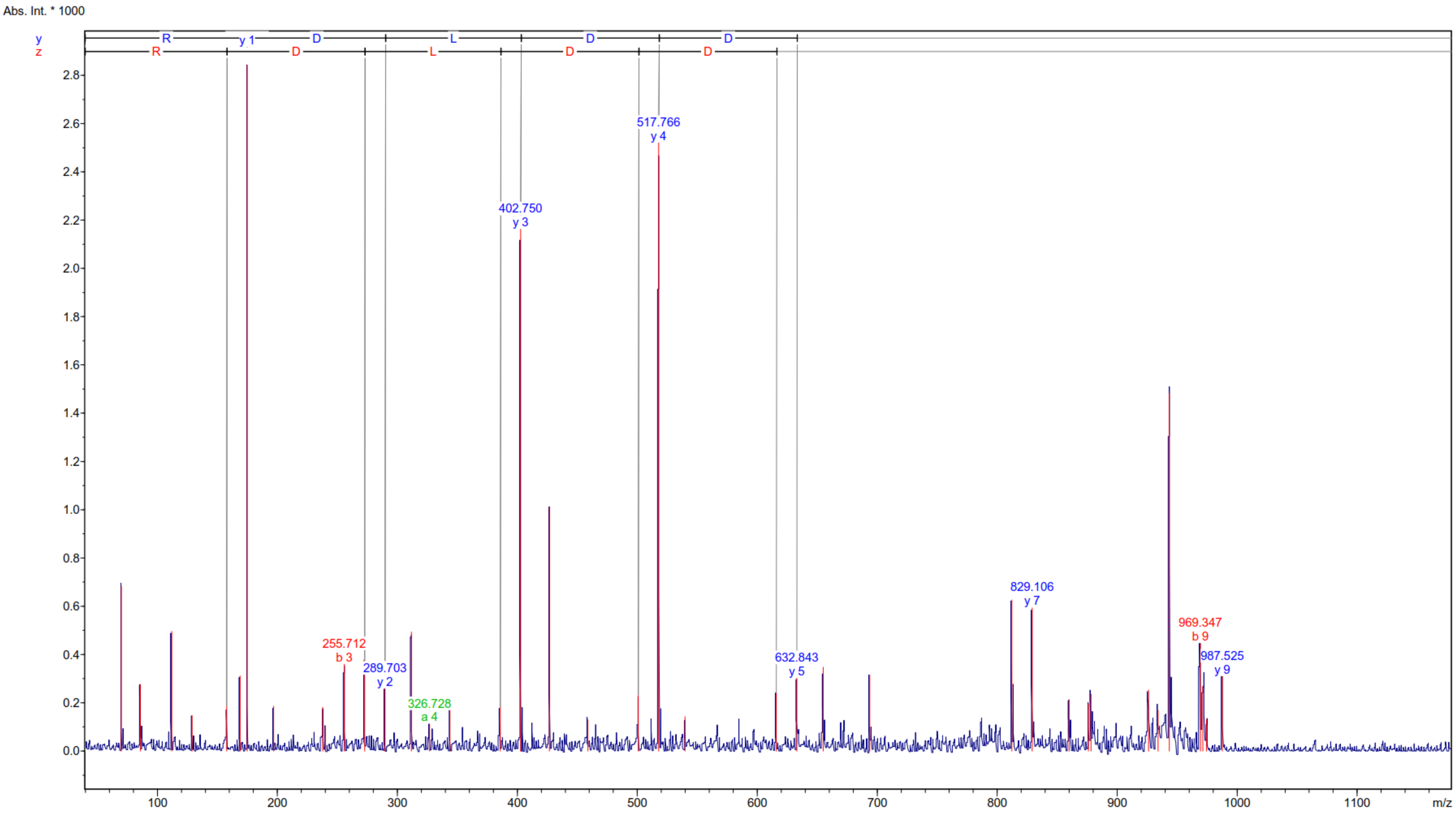

Copyright © 1999-2008 Bruker Daltonik GmbH

Spectrum Analysis Report

Display Parameter:

Parentmass:	987.512	Mass Error:	0.037	MH+ (mono):	987.475	MH+ (avg):	988.032
Threshold (a.i.):	0.000	Tolerance (Da):	0.700	Number of Peaks:	39	Above Threshold:	39
Assigned Peaks:	22	Not assigned Peaks:	17				

Peaklist:

Peak	Mass	Intensity	Peak	Mass	Intensity	Peak	Mass	Intensity	Peak	Mass	Intensity	Peak	Mass	Intensity
1	69.815	684.018	2	85.816	276.200	3	111.787	496.274	4	128.785	147.039	5	157.729	186.200
6	168.764	311.441	7	174.752	2917.397	8	196.754	185.603	9	237.711	181.185	10	239.784	102.448
11	255.712	360.695	12	272.689	376.817	13	289.703	259.730	14	311.710	493.123	15	326.728	112.906
16	343.674	177.824	17	385.730	181.966	18	402.750	2163.096	19	426.711	1012.215	20	458.746	130.883
21	500.747	231.899	22	517.766	2520.476	23	539.767	142.588	24	615.778	239.983	25	632.843	302.546
26	654.849	346.987	27	693.579	314.779	28	812.054	626.531	29	829.106	593.658	30	859.892	214.106
31	875.982	195.875	32	878.122	236.187	33	925.982	253.193	34	933.894	169.089	35	943.332	1482.883
36	969.347	447.133	37	971.336	267.551	38	974.390	131.049	39	987.525	310.009			

Calculated Masses:

GTPVDDLDR

N-Term.	Ion	a	a-17	a-18	b	b-17	b-18	b+18	c	i	x	y	z	C-Term.	Ion
1	G	30.034	13.007	12.023	58.029	41.002	40.018	76.039	75.055	30.034	201.098	**175.119**	**158.092**	9	R
2	T	131.082	114.055	113.071	159.076	142.050	141.066	177.087	176.103	74.060	316.125	**290.146**	**273.119**	8	D
3	P	228.134	211.108	210.124	**256.129**	**239.103**	**238.119**	274.140	**273.156**	**70.065**	429.209	**403.230**	**386.203**	7	L
4	V	**327.203**	310.176	309.192	355.198	338.171	337.187	373.208	372.224	72.081	544.236	**518.257**	**501.230**	6	D
5	D	442.230	425.203	424.219	470.225	453.198	452.214	488.235	487.251	88.039	659.263	**633.284**	**616.257**	5	D
6	D	557.257	**540.230**	**539.246**	585.251	568.225	567.241	603.262	602.278	88.039	758.332	732.352	715.326	4	V
7	L	670.341	653.314	652.330	698.336	681.309	680.325	716.346	715.362	**86.096**	855.384	**829.405**	**812.378**	3	P
8	D	785.368	768.341	767.357	813.362	796.336	795.352	831.373	830.389	88.039	956.432	930.453	913.426	2	T
9	R	941.469	924.442	923.458	**969.464**	952.437	951.453	**987.474**	986.490	**129.113**	1013.453	**987.474**	970.448	1	G

Copyright © 1999-2008 Bruker Daltonik GmbH

PLA$_2$ parentmass 1413.809

Spectrum Analysis Report

Sequence Name:		Parentmass:	1413.809	Mass Error:	0.085	MH+ (mono):	1413.724
MH+ (avg):	1414.694	Threshold (a.i.):	0.000	Tolerance (Da):	0.700	Number of Peaks:	75
Above Threshold:	75	Assigned Peaks:	35	Not assigned Peaks:	40		

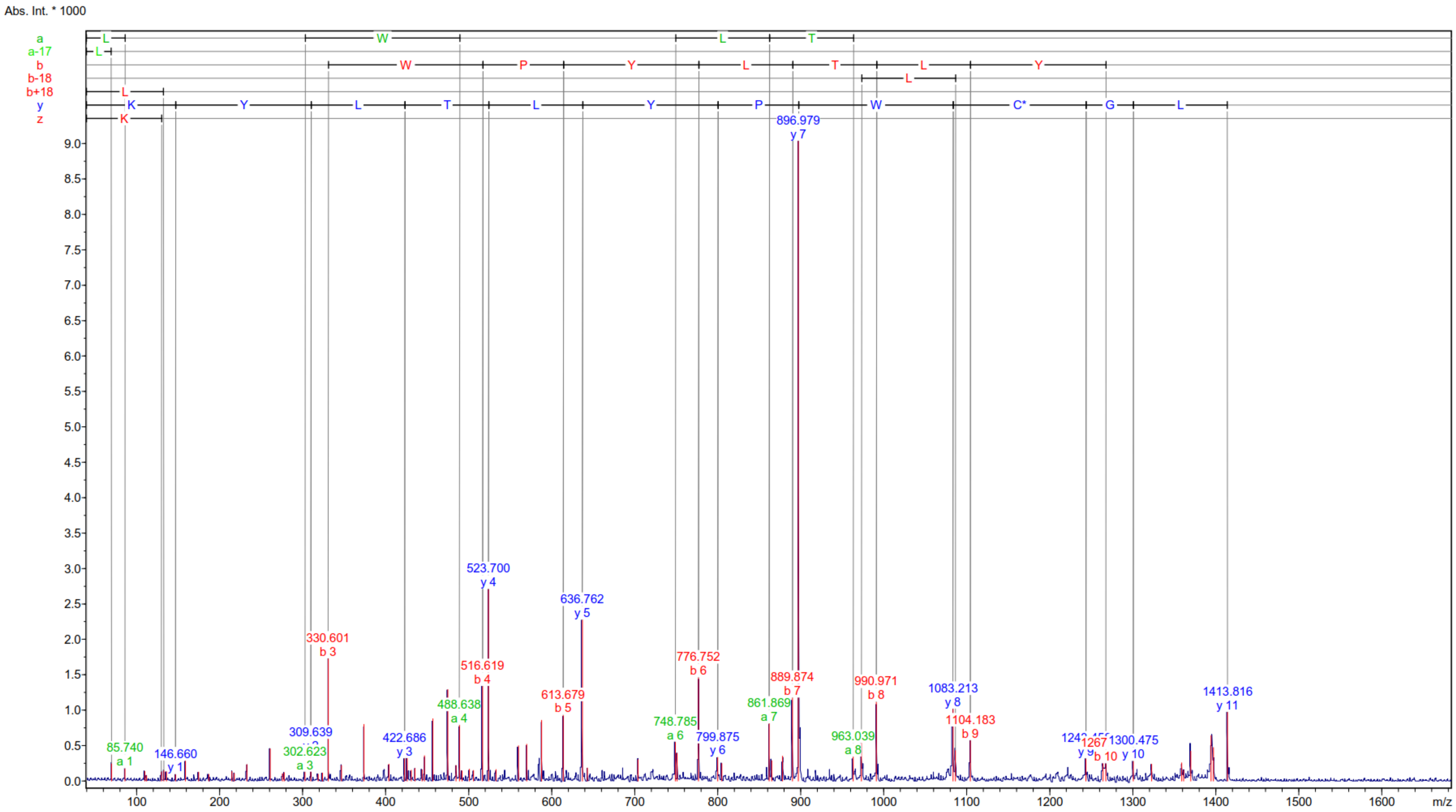

Copyright © 1999-2008 Bruker Daltonik GmbH

Spectrum Analysis Report

Display Parameter:

Parentmass:	1413.809	Mass Error:	0.085	MH+ (mono):	1413.724	MH+ (avg):	1414.694
Threshold (a.i.):	0.000	Tolerance (Da):	0.700	Number of Peaks:	75	Above Threshold:	75
Assigned Peaks:	35	Not assigned Peaks:	40				

Peaklist:

Peak	Mass	Intensity	Peak	Mass	Intensity	Peak	Mass	Intensity	Peak	Mass	Intensity	Peak	Mass	Intensity
1	69.758	247.753	2	85.740	177.976	3	109.648	137.782	4	111.690	75.706	5	129.641	128.878
6	132.619	148.228	7	135.674	132.535	8	146.660	96.296	9	158.636	281.873	10	174.657	125.238
11	186.696	95.610	12	214.636	136.785	13	217.547	109.615	14	232.622	233.519	15	260.603	457.549
16	275.650	104.526	17	277.638	120.298	18	302.623	130.012	19	309.639	401.859	20	318.582	95.227
21	323.606	113.829	22	330.601	1731.837	23	346.575	219.987	24	373.659	800.568	25	403.569	238.062
26	422.686	321.984	27	425.627	613.133	28	430.668	155.141	29	435.077	178.192	30	443.622	160.681
31	446.631	359.841	32	456.670	879.576	33	474.651	1291.454	34	484.637	222.401	35	488.638	790.025
36	491.777	145.509	37	500.612	169.114	38	505.657	149.622	39	516.619	1341.554	40	523.700	2711.543
41	532.625	162.327	42	559.715	498.136	43	569.726	519.855	44	587.719	858.044	45	613.679	930.597
46	636.762	2277.969	47	642.742	183.231	48	703.733	302.860	49	748.785	554.206	50	750.787	391.865
51	776.752	1461.591	52	799.875	336.387	53	804.795	247.180	54	861.869	812.879	55	864.843	287.027
56	877.791	352.371	57	889.874	1175.709	58	896.979	9038.926	59	963.039	342.590	60	972.964	701.423
61	990.971	1117.577	62	1083.213	1019.549	63	1086.167	434.246	64	1104.183	574.337	65	1243.458	318.516
66	1263.864	312.134	67	1267.455	248.838	68	1300.475	281.605	69	1322.529	238.344	70	1358.782	258.993
71	1361.059	155.361	72	1369.696	421.836	73	1394.030	643.694	74	1397.100	475.142	75	1413.816	973.763

Calculated Masses:

LGCWPYLTLYK 3: Carbamidomethyl (C)

N-Term.	Ion	a	a-17	a-18	b	b-17	b-18	b+18	c	i	x	y	z	C-Term.	Ion
1	L	**86.096**	**69.070**	68.086	114.091	97.065	96.081	**132.102**	131.118	**86.096**	173.092	**147.113**	**130.086**	11	K
2	G	143.118	126.091	125.107	171.113	154.086	153.102	189.123	188.139	30.034	336.155	**310.176**	293.150	10	Y
3	C*	**303.149**	286.122	285.138	**331.143**	314.117	313.133	349.154	348.170	**133.043**	449.239	**423.260**	406.234	9	L
4	W	**489.228**	472.201	471.217	**517.223**	**500.196**	499.212	535.233	534.249	**159.092**	550.287	**524.308**	507.281	8	T
5	P	586.281	**569.254**	568.270	**614.276**	597.249	596.265	632.286	631.302	**70.065**	663.371	**637.392**	620.365	7	L
6	Y	**749.344**	732.317	731.333	**777.339**	760.312	759.328	795.349	794.365	**136.076**	826.435	**800.455**	783.429	6	Y
7	L	**862.428**	845.401	844.417	**890.423**	873.396	872.412	908.433	907.449	**86.096**	923.487	**897.508**	880.481	5	P
8	T	**963.476**	946.449	945.465	**991.471**	974.444	**973.460**	1009.481	1008.497	74.060	1109.567	**1083.587**	1066.561	4	W
9	L	1076.560	1059.533	1058.549	**1104.555**	1087.528	**1086.544**	1122.565	1121.581	**86.096**	1269.597	**1243.618**	1226.591	3	C*
10	Y	1239.623	1222.597	1221.613	**1267.618**	1250.591	1249.607	1285.629	1284.645	**136.076**	1326.619	**1300.639**	1283.613	2	G
11	K	1367.718	1350.691	1349.707	1395.713	1378.686	1377.702	**1413.724**	1412.740	101.107	1439.703	**1413.724**	**1396.697**	1	L

Copyright © 1999-2008 Bruker Daltonik GmbH

PLA$_2$ parentmass 1374.965

Spectrum Analysis Report

Sequence Name:
MH+ (avg): 1375.507
Above Threshold: 77
Parentmass: 1374.965
Threshold (a.i.): 0.000
Assigned Peaks: 45
Mass Error: 0.296
Tolerance (Da): 0.700
Not assigned Peaks: 32
MH+ (mono): 1374.669
Number of Peaks: 77

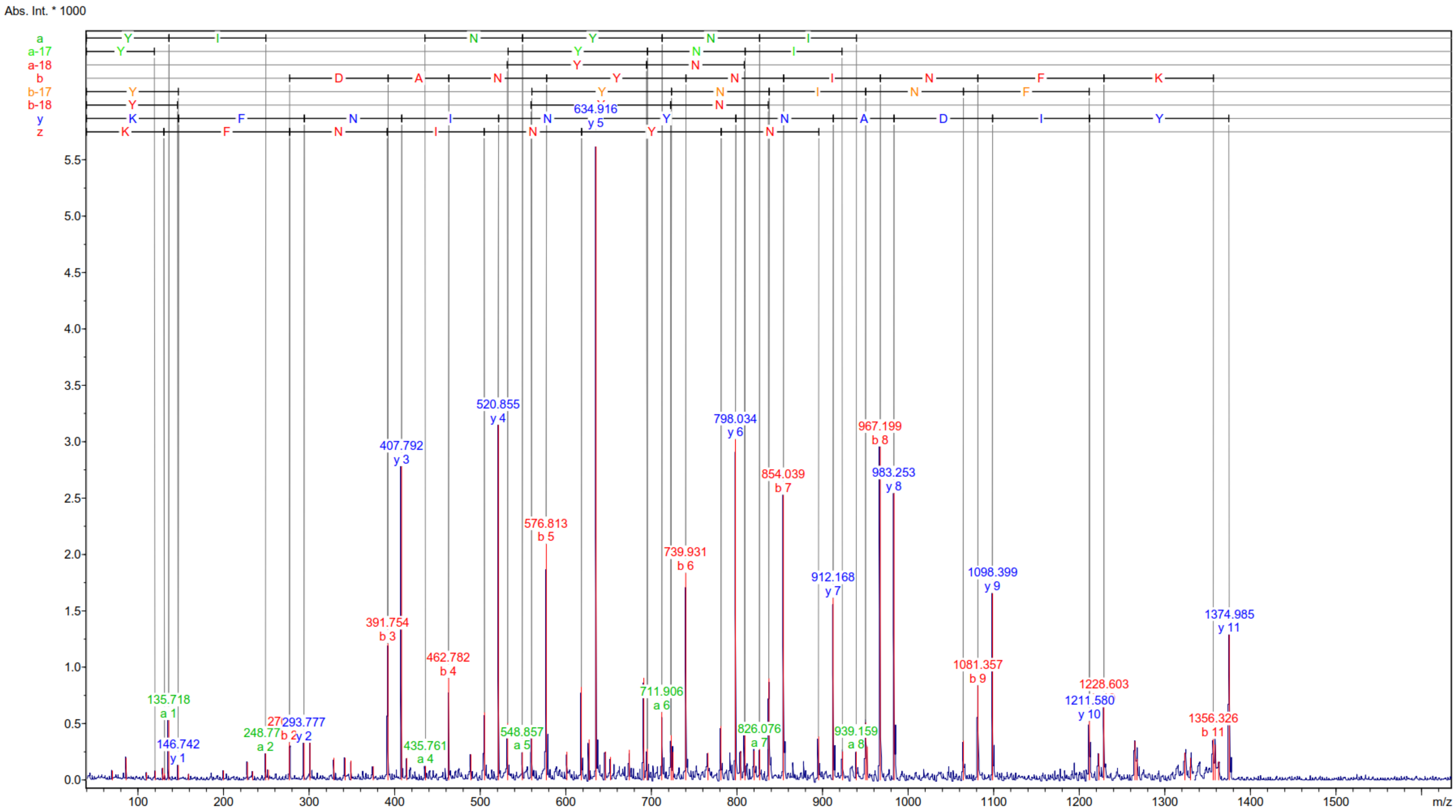

Copyright © 1999-2008 Bruker Daltonik GmbH

Spectrum Analysis Report

Display Parameter:

Parentmass:	1374.965	Mass Error:	0.296	MH+ (mono):	1374.669	MH+ (avg):	1375.507
Threshold (a.i.):	0.000	Tolerance (Da):	0.700	Number of Peaks:	77	Above Threshold:	77
Assigned Peaks:	45	Not assigned Peaks:	32				

Peaklist:

Peak	Mass	Intensity	Peak	Mass	Intensity	Peak	Mass	Intensity	Peak	Mass	Intensity	Peak	Mass	Intensity
1	69.760	80.831	2	85.809	193.858	3	109.730	62.307	4	119.743	80.293	5	128.768	106.130
6	130.728	60.820	7	135.718	529.588	8	146.742	133.809	9	158.706	51.443	10	199.735	80.922
11	227.722	156.291	12	233.684	73.614	13	248.773	234.686	14	252.074	88.513	15	276.744	337.594
16	293.777	329.748	17	300.700	423.174	18	328.739	195.362	19	341.760	194.718	20	348.750	170.379
21	374.747	119.851	22	391.754	1212.735	23	407.792	2782.157	24	413.787	192.163	25	435.761	124.818
26	462.782	904.240	27	488.812	224.368	28	504.812	601.138	29	520.855	3150.010	30	531.820	504.844
31	548.857	246.530	32	559.784	492.621	33	576.813	2093.551	34	600.843	247.337	35	617.880	827.060
36	626.837	357.422	37	634.916	5770.545	38	645.872	250.870	39	651.854	201.738	40	673.847	265.318
41	690.893	903.811	42	694.900	278.561	43	711.906	602.297	44	722.896	399.377	45	724.824	246.655
46	739.931	1836.828	47	765.924	237.760	48	781.013	479.544	49	798.034	3024.154	50	804.045	254.839
51	808.997	443.761	52	819.955	257.305	53	826.076	273.027	54	837.014	902.050	55	854.039	2528.446
56	895.106	384.090	57	912.168	1615.090	58	923.114	254.000	59	939.159	251.922	60	950.155	509.826
61	951.878	294.156	62	967.199	2955.598	63	983.253	2544.674	64	1064.333	350.866	65	1081.357	840.654
66	1098.399	1657.337	67	1211.580	522.936	68	1222.649	232.188	69	1228.603	667.272	70	1264.906	343.240
71	1267.119	184.674	72	1323.231	240.571	73	1329.836	195.455	74	1356.326	363.102	75	1358.453	311.348
76	1362.579	164.423	77	1374.985	1288.762									

Calculated Masses:
YIDANYNINFK

N-Term.	Ion	a	a-17	a-18	b	b-17	b-18	b+18	c	i	x	y	z	C-Term.	Ion
1	Y	**136.076**	**119.049**	118.065	164.071	**147.044**	**146.060**	182.081	181.097	**136.076**	173.092	**147.113**	**130.086**	11	K
2	I	**249.160**	232.133	231.149	**277.155**	260.128	259.144	295.165	**294.181**	**86.096**	320.160	**294.181**	**277.155**	10	F
3	D	364.187	347.160	346.176	**392.182**	**375.155**	**374.171**	410.192	409.208	88.039	434.203	**408.224**	**391.198**	9	N
4	A	**435.224**	418.197	417.213	**463.219**	446.192	445.208	481.229	480.245	44.049	547.287	**521.308**	**504.282**	8	I
5	N	**549.267**	**532.240**	**531.256**	**577.262**	**560.235**	**559.251**	595.272	594.288	87.055	661.330	**635.351**	**618.325**	7	N
6	Y	**712.330**	**695.304**	**694.320**	**740.325**	**723.298**	**722.314**	758.336	757.352	**136.076**	824.394	**798.414**	**781.388**	6	Y
7	N	**826.373**	**809.346**	**808.362**	**854.368**	**837.341**	**836.357**	872.378	871.394	87.055	938.437	**912.457**	**895.431**	5	N
8	I	**939.457**	**922.431**	921.446	**967.452**	**950.425**	949.441	985.463	984.479	**86.096**	1009.474	**983.495**	966.468	4	A
9	N	1053.500	1036.473	1035.489	**1081.495**	**1064.468**	1063.484	1099.505	**1098.521**	87.055	1124.501	**1098.521**	**1081.495**	3	D
10	F	1200.568	1183.542	1182.558	**1228.563**	**1211.537**	1210.553	1246.574	1245.590	**120.081**	1237.585	**1211.606**	1194.579	2	I
11	K	1328.663	1311.637	1310.653	**1356.658**	1339.632	1338.648	**1374.669**	1373.685	101.107	1400.648	**1374.669**	1357.642	1	Y

Copyright © 1999-2008 Bruker Daltonik GmbH

PLA$_2$ parentmass 1512.841

Spectrum Analysis Report

Sequence Name:		Parentmass:	1512.841	Mass Error:	0.274	MH+ (mono):	1512.567
MH+ (avg):	1513.661	Threshold (a.i.):	0.000	Tolerance (Da):	0.700	Number of Peaks:	72
Above Threshold:	72	Assigned Peaks:	38	Not assigned Peaks:	34		

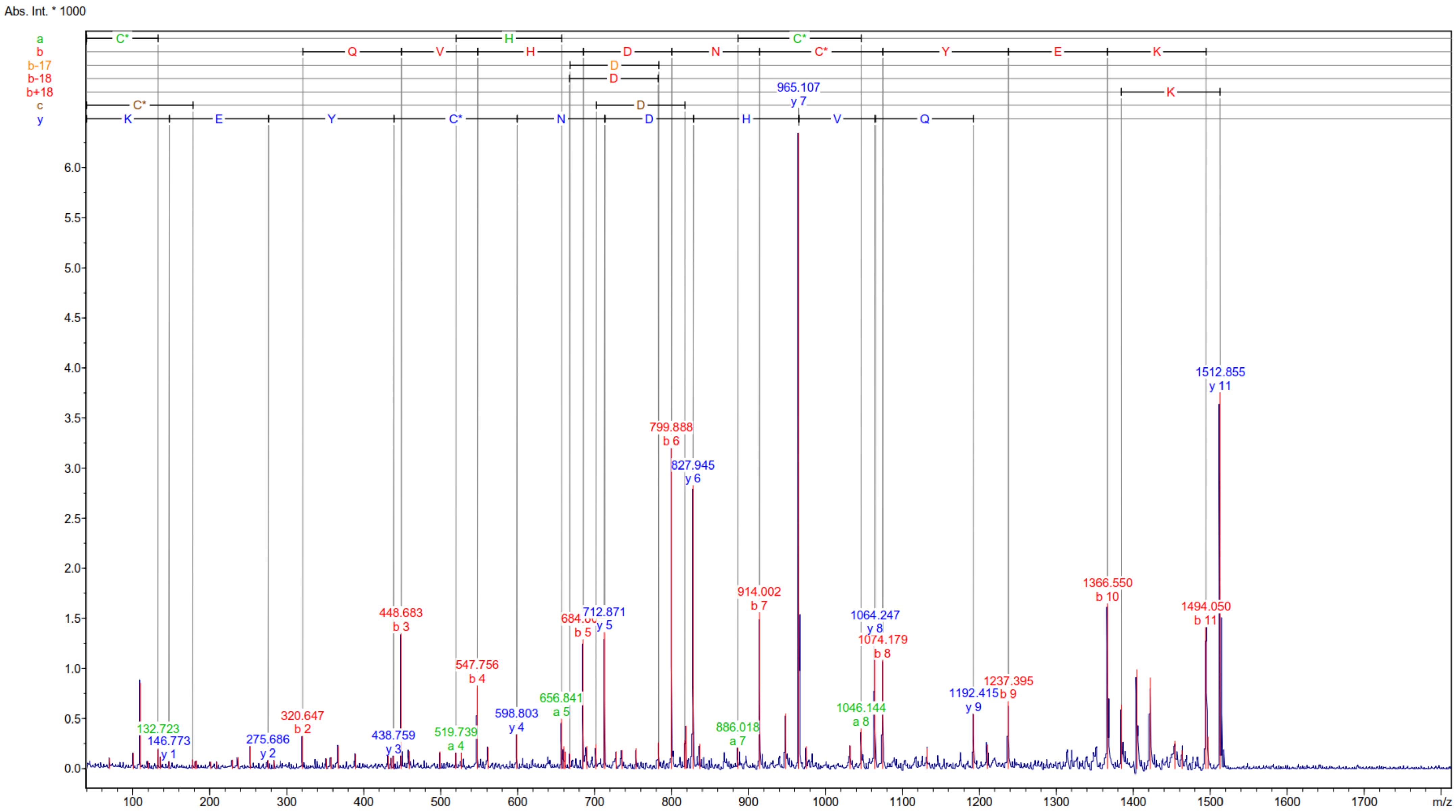

Copyright © 1999-2008 Bruker Daltonik GmbH

Spectrum Analysis Report

Display Parameter:

Parentmass:	1512.841	Mass Error:	0.274	MH+ (mono):	1512.567	MH+ (avg):	1513.661
Threshold (a.i.):	0.000	Tolerance (Da):	0.700	Number of Peaks:	72	Above Threshold:	72
Assigned Peaks:	38	Not assigned Peaks:	34				

Peaklist:

Peak	Mass	Intensity	Peak	Mass	Intensity	Peak	Mass	Intensity	Peak	Mass	Intensity	Peak	Mass	Intensity
1	69.765	105.414	2	100.759	155.402	3	109.751	855.150	4	119.853	74.067	5	132.723	197.074
6	135.716	115.059	7	146.773	74.279	8	177.679	92.621	9	180.675	71.284	10	182.719	79.494
11	201.069	67.196	12	208.712	65.436	13	229.659	86.641	14	236.693	114.134	15	252.672	261.935
16	275.686	87.872	17	283.719	82.500	18	320.647	326.244	19	351.746	99.820	20	357.720	113.661
21	366.716	224.168	22	389.667	148.440	23	431.669	135.152	24	435.706	105.928	25	438.759	132.046
26	448.683	1352.136	27	458.919	174.487	28	498.707	170.346	29	519.739	162.266	30	526.698	259.624
31	547.756	832.232	32	561.507	212.701	33	598.803	345.370	34	656.841	497.099	35	659.994	221.538
36	661.803	167.029	37	667.813	155.253	38	684.806	1290.829	39	689.751	222.696	40	701.822	242.105
41	712.871	1362.469	42	727.885	170.085	43	735.786	185.863	44	753.909	198.710	45	782.867	266.938
46	799.888	3200.925	47	816.924	282.773	48	818.891	428.641	49	827.945	2827.844	50	836.938	244.590
51	886.018	207.920	52	914.002	1559.957	53	948.071	549.597	54	965.107	6594.201	55	975.171	221.043
56	1032.041	227.772	57	1046.144	402.888	58	1064.247	1329.093	59	1074.179	1086.730	60	1131.820	199.880
61	1192.415	547.122	62	1210.411	237.523	63	1237.395	673.626	64	1366.550	1650.021	65	1384.583	640.307
66	1404.605	988.705	67	1421.726	909.445	68	1453.690	274.215	69	1463.326	185.344	70	1494.050	1413.756
71	1497.101	318.595	72	1512.855	3755.724									

Calculated Masses:

CCQVHDNCYEK 1: Carbamidomethyl (C) 2: Carbamidomethyl (C) 8: Carbamidomethyl (C)

N-Term.	Ion	a	a-17	a-18	b	b-17	b-18	b+18	c	i	x	y	z	C-Term.	Ion
1	C*	**133.043**	116.016	115.032	161.038	144.011	143.027	179.048	**178.064**	**133.043**	173.092	**147.113**	130.086	11	K
2	C*	293.074	**276.047**	**275.063**	**321.069**	304.042	303.058	339.079	338.095	**133.043**	302.135	**276.155**	259.129	10	E
3	Q	421.132	404.106	403.122	**449.127**	**432.101**	**431.117**	467.138	466.154	**101.071**	465.198	**439.219**	422.192	9	Y
4	V	**520.201**	503.174	502.190	**548.196**	531.169	530.185	566.206	565.222	72.081	625.229	**599.249**	582.223	8	C*
5	H	**657.260**	640.233	639.249	**685.254**	**668.228**	**667.244**	703.265	**702.281**	**110.071**	739.272	**713.292**	696.266	7	N
6	D	772.287	755.260	**754.276**	**800.281**	**783.255**	**782.271**	**818.292**	**817.308**	88.039	854.299	**828.319**	811.293	6	D
7	N	**886.329**	869.303	868.319	**914.324**	897.298	896.314	932.335	931.351	87.055	991.357	**965.378**	**948.352**	5	H
8	C*	**1046.360**	1029.334	1028.350	**1074.355**	1057.328	1056.344	1092.366	1091.382	**133.043**	1090.426	**1064.447**	1047.420	4	V
9	Y	1209.423	**1192.397**	1191.413	**1237.418**	1220.392	1219.408	1255.429	1254.445	**136.076**	1218.484	**1192.505**	1175.479	3	Q
10	E	1338.466	1321.439	1320.455	**1366.461**	1349.434	1348.450	**1384.471**	1383.487	102.055	1378.515	1352.536	1335.509	2	C*
11	K	1466.561	1449.534	1448.550	**1494.556**	1477.529	1476.545	**1512.566**	1511.582	**101.107**	1538.546	**1512.566**	1495.540	1	C*

Copyright © 1999-2008 Bruker Daltonik GmbH

PLA$_2$ parentmass 2157.377

Spectrum Analysis Report

Sequence Name:		Parentmass:	2157.377	Mass Error:	0.467	MH+ (mono):	2156.910
MH+ (avg):	2158.451	Threshold (a.i.):	0.000	Tolerance (Da):	1.000	Number of Peaks:	119
Above Threshold:	119	Assigned Peaks:	70	Not assigned Peaks:	49		

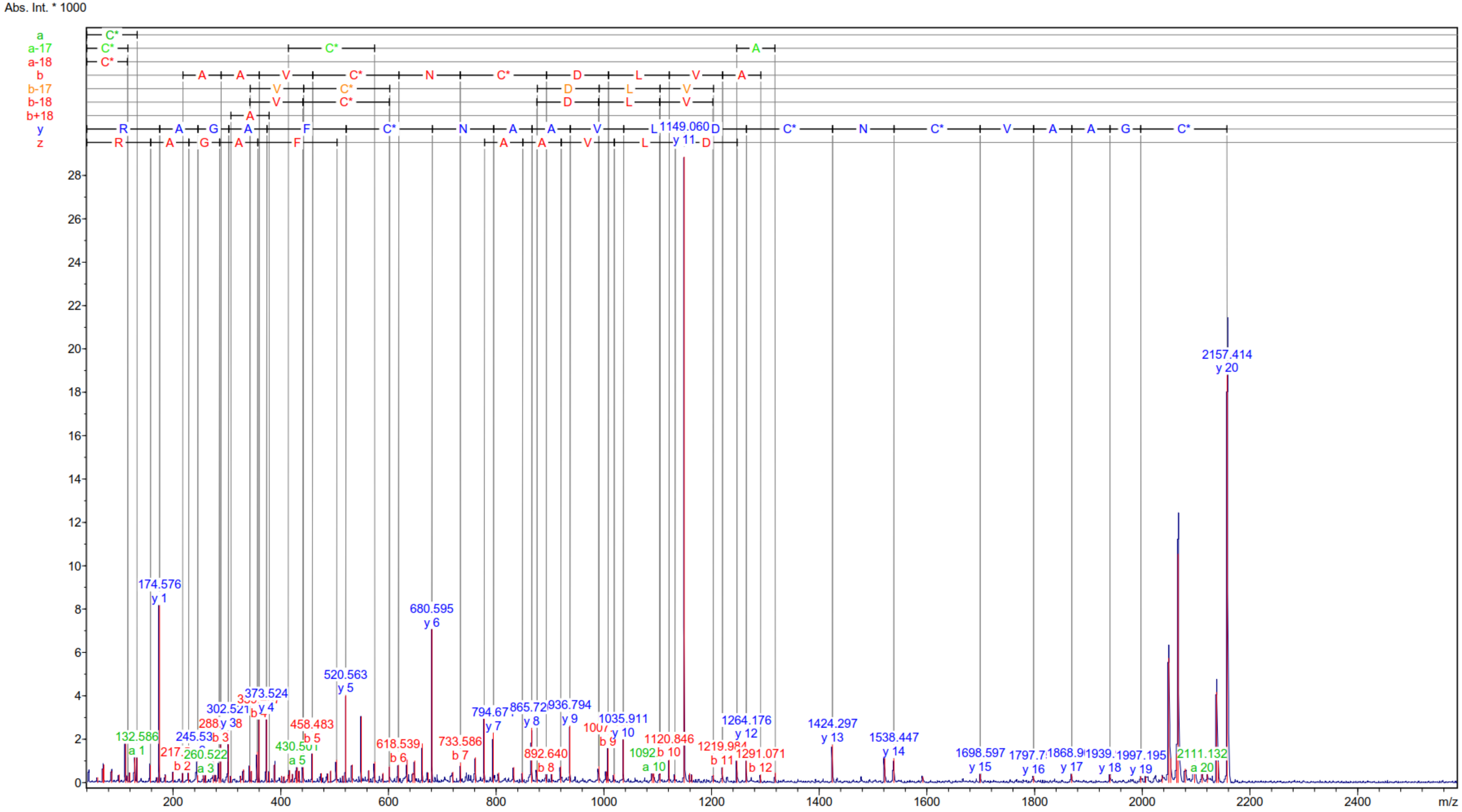

Copyright © 1999-2008 Bruker Daltonik GmbH

Spectrum Analysis Report

Display Parameter:

Parentmass:	2157.377	Mass Error:	0.467	MH+ (mono):	2156.910	MH+ (avg):	2158.451
Threshold (a.i.):	0.000	Tolerance (Da):	1.000	Number of Peaks:	119	Above Threshold:	119
Assigned Peaks:	70	Not assigned Peaks:	49				

Peaklist:

Peak	Mass	Intensity	Peak	Mass	Intensity	Peak	Mass	Intensity	Peak	Mass	Intensity	Peak	Mass	Intensity
1	59.757	239.650	2	69.697	633.067	3	71.706	827.641	4	85.682	532.256	5	99.645	360.432
6	111.636	2273.177	7	115.666	306.060	8	119.620	456.591	9	128.617	1134.624	10	132.586	1156.037
11	157.598	823.558	12	170.590	231.024	13	174.576	8181.252	14	185.563	335.828	15	199.582	469.421
16	217.501	438.826	17	228.534	561.622	18	245.531	1170.841	19	256.508	380.802	20	260.522	326.951
21	274.452	1544.024	22	279.463	334.092	23	285.520	886.466	24	288.468	1761.058	25	302.521	2448.717
26	307.474	298.811	27	325.479	305.653	28	330.481	601.369	29	342.453	789.415	30	345.455	525.222
31	356.501	1424.989	32	359.467	2900.007	33	373.524	3152.554	34	378.456	500.804	35	388.457	825.870
36	401.496	299.918	37	406.458	281.085	38	413.492	362.346	39	416.468	554.288	40	421.446	382.504
41	428.505	549.565	42	430.501	698.637	43	434.395	533.416	44	441.472	1190.614	45	458.483	1729.922
46	475.486	423.810	47	487.520	400.846	48	492.476	546.039	49	503.522	1042.363	50	515.504	311.386
51	520.563	4030.591	52	532.433	810.838	53	549.440	3041.273	54	563.507	554.775	55	574.570	952.986
56	589.589	436.613	57	601.546	748.359	58	618.539	810.968	59	634.543	1058.436	60	645.520	440.518
61	648.503	1002.255	62	662.533	1801.241	63	673.534	454.785	64	680.595	7078.807	65	719.562	485.800
66	733.586	930.266	67	761.610	1158.852	68	777.643	3121.730	69	794.671	2307.713	70	804.654	451.707
71	832.677	703.486	72	848.648	476.122	73	865.720	2526.557	74	875.699	607.531	75	892.640	377.998
76	903.692	408.604	77	919.733	763.340	78	936.794	2625.978	79	990.703	757.323	80	1004.860	501.384
81	1007.749	1580.478	82	1018.808	393.415	83	1035.911	1989.835	84	1089.943	897.591	85	1092.877	415.047
86	1103.864	484.908	87	1120.846	1074.166	88	1131.897	354.731	89	1149.060	29330.044	90	1159.057	420.935
91	1163.075	340.930	92	1202.980	370.257	93	1219.984	703.553	94	1247.133	1489.734	95	1264.176	1933.132
96	1291.071	372.413	97	1318.219	462.333	98	1424.297	1751.637	99	1521.383	1140.292	100	1538.447	1125.620
101	1592.465	265.461	102	1698.597	405.434	103	1797.758	300.945	104	1868.904	404.862	105	1939.925	383.089
106	1997.195	295.380	107	2006.153	267.091	108	2037.018	307.094	109	2049.171	5744.442	110	2052.672	1111.264
111	2063.958	1328.731	112	2066.390	10547.850	113	2079.519	632.654	114	2096.814	1210.203	115	2111.132	381.282
116	2120.571	461.005	117	2137.758	4165.351	118	2140.854	949.528	119	2157.414	18809.611			

Calculated Masses:

CGAAVCNCDLVAANCFAGAR 1: Carbamidomethyl (C) 6: Carbamidomethyl (C) 8: Carbamidomethyl (C) 15: Carbamidomethyl (C)

N-Term.	Ion	a	a-17	a-18	b	b-17	b-18	b+18	c	i	x	y	z	C-Term.	Ion
1	C*	**133.043**	**116.016**	**115.032**	161.038	144.011	143.027	179.048	178.064	**133.043**	201.098	**175.119**	**158.092**	20	R
2	G	190.064	173.038	172.054	**218.059**	201.033	**200.049**	236.070	235.086	30.034	272.135	**246.156**	**229.130**	19	A
3	A	**261.102**	244.075	243.091	**289.097**	272.070	271.086	**307.107**	306.123	44.049	329.157	**303.178**	**286.151**	18	G
4	A	332.139	315.112	314.128	**360.134**	**343.107**	**342.123**	**378.144**	377.160	44.049	400.194	**374.215**	**357.188**	17	A
5	V	**431.207**	**414.181**	**413.197**	**459.202**	**442.175**	**441.191**	477.213	**476.229**	**72.081**	547.262	**521.283**	**504.257**	16	F
6	C*	591.238	**574.211**	573.227	**619.233**	**602.206**	**601.222**	637.243	636.259	**133.043**	707.293	**681.314**	664.287	15	C*
7	N	705.281	688.254	687.270	**733.276**	716.249	715.265	751.286	750.302	87.055	821.336	**795.357**	**778.330**	14	N
8	C*	**865.311**	**848.285**	847.301	**893.306**	**876.280**	**875.296**	911.317	910.333	**133.043**	**892.373**	**866.394**	**849.367**	13	A
9	D	980.338	963.312	962.328	**1008.333**	**991.307**	**990.323**	1026.344	1025.360	88.039	963.410	**937.431**	**920.404**	12	A
10	L	**1093.422**	1076.396	1075.412	**1121.417**	**1104.391**	**1103.407**	1139.428	1138.444	**86.096**	1062.479	**1036.499**	**1019.473**	11	V
11	V	1192.491	1175.464	1174.480	**1220.486**	**1203.459**	**1202.475**	1238.496	1237.512	**72.081**	1175.563	**1149.583**	**1132.557**	10	L
12	A	**1263.528**	**1246.501**	1245.517	**1291.523**	1274.496	1273.512	1309.533	1308.549	44.049	**1290.590**	**1264.610**	**1247.584**	9	D
13	A	1334.565	**1317.538**	1316.554	1362.560	1345.533	1344.549	1380.570	1379.586	44.049	1450.620	**1424.641**	1407.614	8	C*
14	N	1448.608	1431.581	1430.597	1476.603	1459.576	1458.592	1494.613	1493.629	87.055	1564.663	**1538.684**	**1521.657**	7	N
15	C*	1608.639	**1591.612**	1590.628	1636.633	1619.607	1618.623	1654.644	1653.660	**133.043**	1724.694	**1698.715**	1681.688	6	C*
16	F	1755.707	1738.680	1737.696	1783.702	1766.675	1765.691	1801.712	1800.728	**120.081**	1823.762	**1797.783**	1780.756	5	V
17	A	1826.744	1809.718	1808.734	1854.739	1837.712	1836.728	1872.750	1871.766	44.049	1894.799	**1868.820**	1851.793	4	A
18	G	1883.766	1866.739	1865.755	1911.760	1894.734	1893.750	1929.771	1928.787	30.034	1965.836	**1939.857**	1922.831	3	A
19	A	1954.803	1937.776	1936.792	1982.798	1965.771	1964.787	2000.808	1999.824	44.049	2022.858	**1996.879**	1979.852	2	G
20	R	**2110.904**	2093.877	2092.893	2138.899	2121.872	**2120.888**	**2156.909**	2155.925	**129.113**	2182.889	**2156.909**	**2139.883**	1	C*

Copyright © 1999-2008 Bruker Daltonik GmbH

PLA$_2$ parentmass 1282.633

Spectrum Analysis Report

Sequence Name:		Parentmass:	1282.633	Mass Error:	-1281.448	MH+ (mono):	2564.081
MH+ (avg):	2565.854	Threshold (a.i.):	0.000	Tolerance (Da):	0.700	Number of Peaks:	45
Above Threshold:	45	Assigned Peaks:	20	Not assigned Peaks:	25		

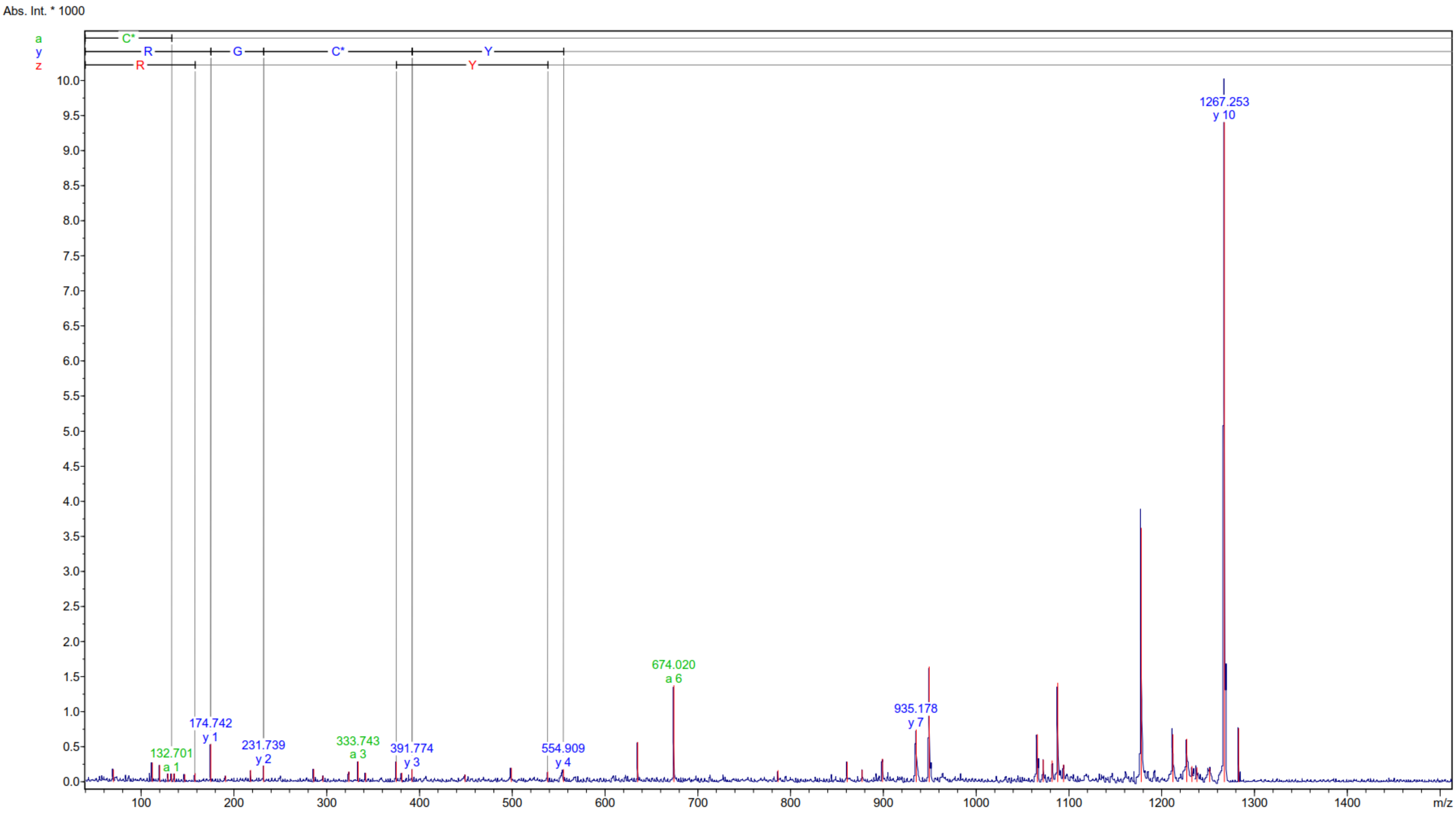

Copyright © 1999-2008 Bruker Daltonik GmbH

Display Parameter:

Parentmass:	1282.633	Mass Error:	-1281.448	MH+ (mono):	2564.081	MH+ (avg):	2565.854
Threshold (a.i.):	0.000	Tolerance (Da):	0.700	Number of Peaks:	45	Above Threshold:	45
Assigned Peaks:	20	Not assigned Peaks:	25				

Peaklist:

Peak	Mass	Intensity	Peak	Mass	Intensity	Peak	Mass	Intensity	Peak	Mass	Intensity	Peak	Mass	Intensity
1	69.771	173.126	2	111.754	271.256	3	119.741	235.020	4	128.743	191.840	5	132.701	118.291
6	135.746	352.677	7	146.718	103.336	8	157.735	122.066	9	174.742	540.559	10	190.734	83.144
11	217.683	166.196	12	231.739	232.087	13	285.760	178.769	14	295.748	83.143	15	323.726	142.544
16	333.743	292.533	17	341.752	120.367	18	374.756	287.914	19	380.717	124.366	20	391.774	181.951
21	448.796	100.827	22	498.793	195.291	23	537.876	144.595	24	554.909	178.969	25	635.028	563.021
26	674.020	1375.995	27	786.066	161.694	28	860.869	281.757	29	876.834	169.053	30	898.817	326.916
31	935.178	746.419	32	949.238	1638.368	33	1065.864	673.017	34	1072.097	312.206	35	1081.656	301.835
36	1087.830	1407.647	37	1094.117	241.323	38	1177.717	3617.273	39	1211.737	673.666	40	1226.843	608.556
41	1232.361	220.213	42	1237.635	202.337	43	1251.450	194.043	44	1267.253	9404.990	45	1282.653	761.214

Calculated Masses:

CTVPSRSWWHFANYGCYCGR 1: Carbamidomethyl (C) 16: Carbamidomethyl (C) 18: Carbamidomethyl (C)

N-Term.	Ion	a	a-17	a-18	b	b-17	b-18	b+18	c	i	x	y	z	C-Term.	Ion
1	C*	**133.043**	116.016	115.032	161.038	144.011	143.027	179.048	178.064	**133.043**	201.098	**175.119**	**158.092**	20	R
2	T	234.091	**217.064**	216.080	262.086	245.059	244.075	280.096	279.112	74.060	258.120	**232.140**	215.114	19	G
3	V	**333.159**	316.133	315.149	361.154	344.127	343.143	379.165	378.181	72.081	418.150	**392.171**	**375.145**	18	C*
4	P	430.212	413.185	412.201	458.207	441.180	440.196	476.217	475.233	**70.065**	581.214	**555.234**	**538.208**	17	Y
5	S	517.244	500.217	**499.233**	545.239	528.212	527.228	563.249	562.265	60.044	741.244	715.265	698.238	16	C*
6	R	**673.345**	656.318	655.334	701.340	684.313	683.329	719.350	718.366	**129.113**	798.266	772.287	755.260	15	G
7	S	760.377	743.350	742.366	788.372	771.345	770.361	806.383	805.398	60.044	961.329	**935.350**	918.323	14	Y
8	W	946.456	929.430	928.446	974.451	957.425	956.441	992.462	991.478	159.092	1075.372	1049.393	1032.366	13	N
9	W	1132.536	1115.509	1114.525	1160.531	1143.504	1142.520	1178.541	**1177.557**	159.092	1146.409	1120.430	1103.403	12	A
10	H	1269.595	1252.568	**1251.584**	1297.589	1280.563	1279.579	1315.600	1314.616	110.071	1293.478	**1267.498**	1250.472	11	F
11	F	1416.663	1399.636	1398.652	1444.658	1427.631	1426.647	1462.668	1461.684	**120.081**	1430.536	1404.557	1387.531	10	H
12	A	1487.700	1470.674	1469.690	1515.695	1498.668	1497.684	1533.706	1532.722	44.049	1616.616	1590.637	1573.610	9	W
13	N	1601.743	1584.716	1583.732	1629.738	1612.711	1611.727	1647.749	1646.764	87.055	1802.695	1776.716	1759.689	8	W
14	Y	1764.806	1747.780	1746.796	1792.801	1775.775	1774.791	1810.812	1809.828	**136.076**	1889.727	1863.748	1846.721	7	S
15	G	1821.828	1804.801	1803.817	1849.823	1832.796	1831.812	1867.833	1866.849	30.034	2045.828	2019.849	2002.822	6	R
16	C*	1981.858	1964.832	1963.848	2009.853	1992.827	1991.843	2027.864	2026.880	**133.043**	2132.860	2106.881	2089.854	5	S
17	Y	2144.922	2127.895	2126.911	2172.917	2155.890	2154.906	2190.927	2189.943	**136.076**	2229.913	2203.934	2186.907	4	P
18	C*	2304.952	2287.926	2286.942	2332.947	2315.921	2314.937	2350.958	2349.974	**133.043**	2328.981	2303.002	2285.976	3	V
19	G	2361.974	2344.947	2343.963	2389.969	2372.942	2371.958	2407.979	2406.995	30.034	2430.029	2404.050	2387.023	2	T
20	R	2518.075	2501.048	2500.064	2546.070	2529.043	2528.059	2564.080	2563.096	**129.113**	2590.060	2564.080	2547.054	1	C*

Copyright © 1999-2008 Bruker Daltonik GmbH

VNGF parentmass 1127.664

Spectrum Analysis Report

Sequence Name:		Parentmass:	1127.664	Mass Error:	0.174	MH+ (mono):	1127.490
MH+ (avg):	1128.200	Threshold (a.i.):	0.000	Tolerance (Da):	0.700	Number of Peaks:	61
Above Threshold:	61	Assigned Peaks:	35	Not assigned Peaks:	26		

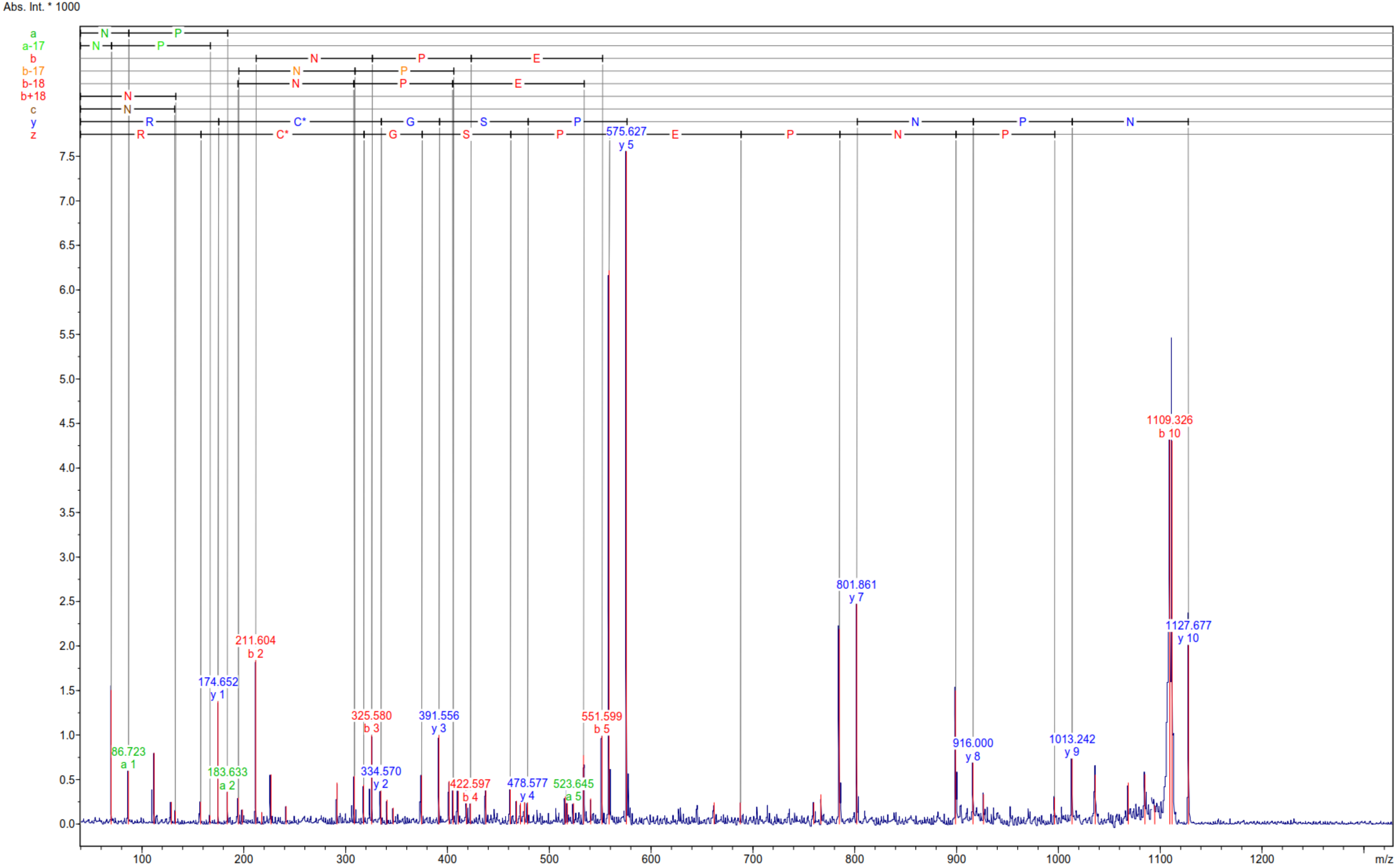

Copyright © 1999-2008 Bruker Daltonik GmbH

Spectrum Analysis Report

Display Parameter:

Parentmass:	1127.664	Mass Error:	0.174	MH+ (mono):	1127.490	MH+ (avg):	1128.200
Threshold (a.i.):	0.000	Tolerance (Da):	0.700	Number of Peaks:	61	Above Threshold:	61
Assigned Peaks:	35	Not assigned Peaks:	26				

Peaklist:

Peak	Mass	Intensity	Peak	Mass	Intensity	Peak	Mass	Intensity	Peak	Mass	Intensity	Peak	Mass	Intensity
1	69.755	1507.692	2	86.723	597.252	3	111.705	796.479	4	128.702	244.809	5	132.630	149.101
6	157.638	250.701	7	166.628	98.065	8	174.652	1381.300	9	183.633	364.591	10	194.589	298.395
11	198.629	162.054	12	211.604	1843.031	13	217.578	129.911	14	226.603	555.959	15	241.588	198.407
16	291.545	462.614	17	308.572	546.032	18	317.565	443.564	19	325.580	1002.996	20	334.570	375.074
21	340.571	271.084	22	346.584	181.385	23	374.563	554.517	24	391.556	1003.097	25	401.546	480.078
26	405.574	425.831	27	410.591	370.445	28	418.573	480.966	29	422.597	232.512	30	437.563	498.367
31	461.564	525.677	32	467.616	257.080	33	471.589	234.283	34	475.597	273.751	35	478.577	243.804
36	515.608	304.774	37	517.590	453.903	38	523.645	234.509	39	533.612	771.743	40	540.591	285.278
41	551.599	993.295	42	558.605	6222.914	43	575.627	7559.209	44	661.685	240.402	45	687.632	242.391
46	759.794	236.459	47	766.783	330.824	48	784.788	2192.744	49	801.861	2473.317	50	898.959	1503.160
51	916.000	691.797	52	926.399	333.543	53	996.143	302.459	54	1013.242	734.762	55	1035.933	552.829
56	1068.419	463.602	57	1084.917	557.547	58	1094.525	208.957	59	1109.326	4317.054	60	1111.385	4302.690
61	1127.677	2012.359												

Calculated Masses:
NPNPEPSGCR 9: Carbamidomethyl (C)

N-Term.	Ion	a	a-17	a-18	b	b-17	b-18	b+18	c	i	x	y	z	C-Term.	Ion
1	N	**87.055**	**70.029**	69.045	115.050	98.024	97.040	**133.061**	**132.077**	**87.055**	201.098	**175.119**	**158.092**	10	R
2	P	**184.108**	**167.082**	**166.097**	**212.103**	**195.076**	**194.092**	230.114	229.130	**70.065**	361.129	**335.150**	**318.123**	9	C*
3	N	298.151	281.124	280.140	**326.146**	**309.119**	**308.135**	344.156	343.172	**87.055**	**418.150**	**392.171**	**375.145**	8	G
4	P	395.204	378.177	377.193	**423.199**	**406.172**	**405.188**	441.209	440.225	**70.065**	505.182	**479.203**	**462.177**	7	S
5	E	**524.246**	507.220	506.236	**552.241**	535.215	**534.231**	570.252	569.268	102.055	602.235	**576.256**	**559.229**	6	P
6	P	621.299	604.273	603.289	649.294	632.267	631.283	667.305	666.321	**70.065**	731.278	705.298	**688.272**	5	E
7	S	708.331	691.305	690.321	736.326	719.299	718.315	754.337	753.353	60.044	828.330	**802.351**	**785.325**	4	P
8	G	765.353	748.326	747.342	793.348	776.321	775.337	811.358	810.374	30.034	942.373	**916.394**	**899.368**	3	N
9	C*	925.383	908.357	907.373	953.378	936.352	935.368	971.389	970.405	**133.043**	1039.426	**1013.447**	**996.420**	2	P
10	R	1081.484	1064.458	1063.474	**1109.479**	1092.453	1091.469	**1127.490**	1126.506	**129.113**	1153.469	**1127.490**	1110.463	1	N

Copyright © 1999-2008 Bruker Daltonik GmbH

VNGF parentmass 1648.000

Spectrum Analysis Report

Sequence Name:		Parentmass:	1648.000	Mass Error:	0.199	MH+ (mono):	1647.801
MH+ (avg):	1648.818	Threshold (a.i.):	0.000	Tolerance (Da):	0.700	Number of Peaks:	57
Above Threshold:	57	Assigned Peaks:	26	Not assigned Peaks:	31		

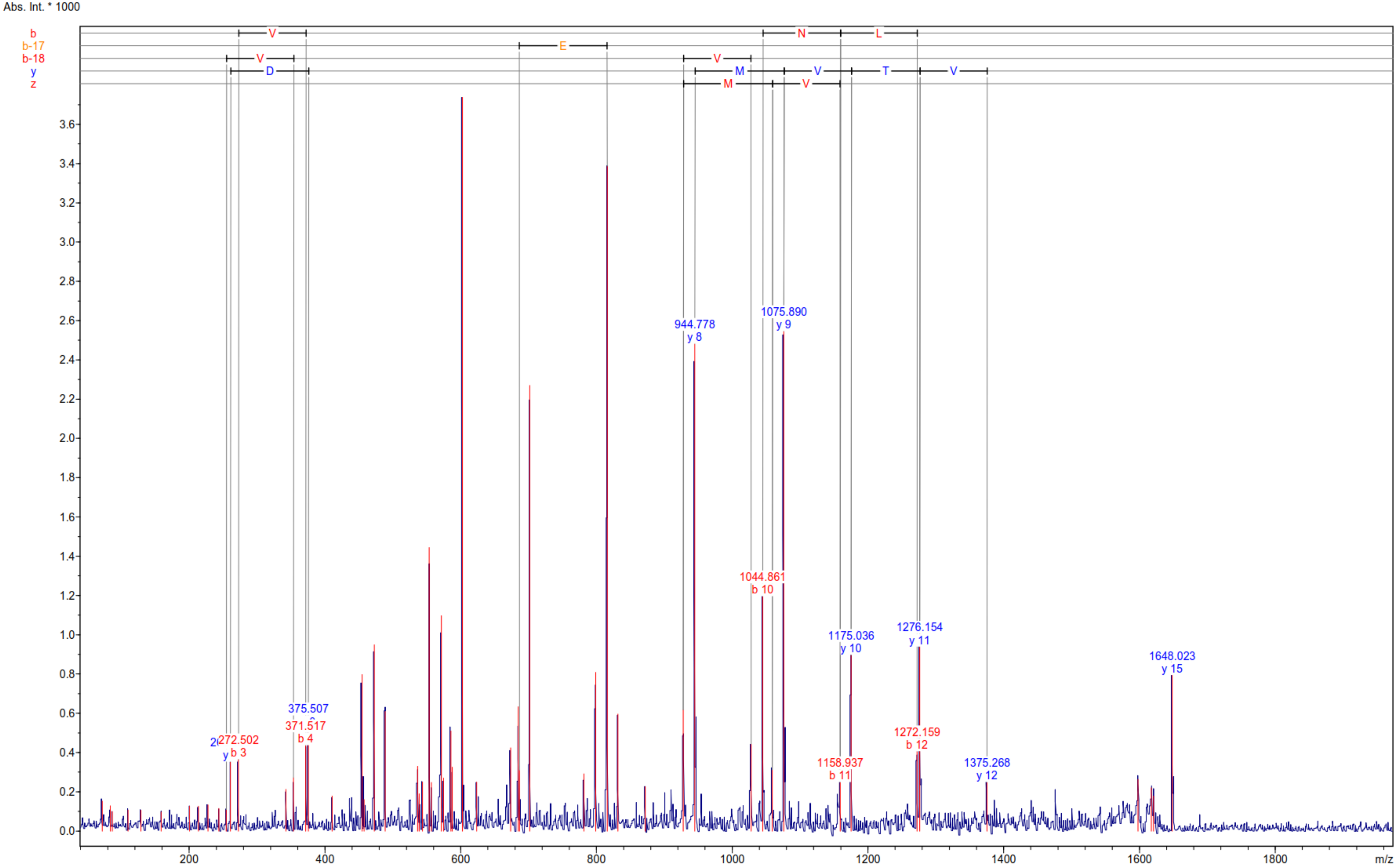

Copyright © 1999-2008 Bruker Daltonik GmbH

Spectrum Analysis Report

Display Parameter:

Parentmass:	1648.000	Mass Error:	0.199	MH+ (mono):	1647.801	MH+ (avg):	1648.818
Threshold (a.i.):	0.000	Tolerance (Da):	0.700	Number of Peaks:	57	Above Threshold:	57
Assigned Peaks:	26	Not assigned Peaks:	31				

Peaklist:

Peak	Mass	Intensity	Peak	Mass	Intensity	Peak	Mass	Intensity	Peak	Mass	Intensity	Peak	Mass	Intensity
1	71.701	153.521	2	83.646	129.326	3	86.640	99.898	4	109.604	108.468	5	128.618	106.598
6	158.570	98.348	7	200.557	127.623	8	213.551	127.567	9	227.532	133.943	10	243.515	116.409
11	254.523	109.147	12	260.539	353.619	13	272.502	365.600	14	342.503	213.325	15	353.520	272.174
16	371.517	437.234	17	375.507	522.359	18	410.479	178.861	19	454.521	797.927	20	457.489	161.246
21	472.515	949.732	22	488.550	609.998	23	536.519	330.934	24	538.626	190.396	25	543.587	250.492
26	553.560	1444.672	27	556.598	247.851	28	571.563	1097.314	29	574.523	271.175	30	585.514	510.408
31	587.522	325.809	32	602.563	3785.897	33	623.546	250.666	34	673.581	425.267	35	684.583	633.649
36	686.634	310.486	37	701.627	2271.209	38	781.613	291.902	39	798.639	809.697	40	815.683	3387.074
41	831.666	596.108	42	871.633	226.805	43	927.754	617.875	44	944.778	2481.937	45	1027.794	443.154
46	1044.861	1196.190	47	1058.839	311.853	48	1075.890	2546.124	49	1158.937	248.916	50	1175.036	896.967
51	1272.159	405.875	52	1276.154	938.779	53	1375.268	248.876	54	1597.602	264.212	55	1617.431	220.772
56	1620.616	179.110	57	1648.023	794.132									

Calculated Masses:
GNTVTVMENVNLDNK

N-Term.	Ion	a	a-17	a-18	b	b-17	b-18	b+18	c	i	x	y	z	C-Term.	Ion
1	G	30.034	13.007	12.023	58.029	41.002	40.018	76.039	75.055	30.034	173.092	147.113	130.086	15	K
2	N	144.077	127.050	126.066	172.072	155.045	154.061	190.082	189.098	**87.055**	287.135	**261.156**	**244.129**	14	N
3	T	245.124	**228.098**	**227.114**	**273.119**	256.093	**255.109**	291.130	290.146	74.060	402.162	**376.183**	359.156	13	D
4	V	344.193	327.166	326.182	**372.188**	355.161	**354.177**	390.198	389.214	**72.081**	515.246	489.267	**472.240**	12	L
5	T	445.241	428.214	427.230	473.235	456.209	455.225	491.246	490.262	74.060	629.289	603.310	586.283	11	N
6	V	544.309	527.282	526.298	572.304	555.277	554.293	590.314	589.330	**72.081**	728.357	702.378	685.352	10	V
7	M	675.349	658.323	657.339	703.344	**686.318**	685.334	721.355	720.371	104.053	842.400	816.421	799.394	9	N
8	E	804.392	787.365	786.381	832.387	**815.360**	814.376	850.397	849.413	102.055	971.443	**945.464**	**928.437**	8	E
9	N	918.435	901.408	900.424	946.430	929.403	**928.419**	964.440	963.456	**87.055**	1102.483	**1076.504**	**1059.478**	7	M
10	V	1017.503	1000.477	999.493	**1045.498**	**1028.472**	**1027.488**	1063.509	1062.525	**72.081**	1201.552	**1175.572**	**1158.546**	6	V
11	N	1131.546	1114.520	1113.536	**1159.541**	1142.515	1141.531	1177.552	1176.568	**87.055**	1302.599	**1276.620**	1259.594	5	T
12	L	1244.630	1227.604	1226.620	**1272.625**	1255.599	1254.615	1290.636	1289.652	**86.096**	1401.668	**1375.689**	1358.662	4	V
13	D	1359.657	1342.631	1341.647	1387.652	1370.626	1369.642	1405.663	1404.679	88.039	1502.716	1476.736	1459.710	3	T
14	N	1473.700	1456.674	1455.690	1501.695	1484.669	1483.685	1519.706	1518.722	**87.055**	**1616.758**	1590.779	1573.753	2	N
15	K	1601.795	1584.769	1583.785	1629.790	1612.764	1611.780	**1647.801**	1646.817	101.107	1673.780	**1647.801**	1630.774	1	G

Copyright © 1999-2008 Bruker Daltonik GmbH

VNGF parentmass 1415.821

Spectrum Analysis Report

Sequence Name:		Parentmass:	1415.821	Mass Error:	0.205	MH+ (mono):	1415.616
MH+ (avg):	1416.568	Threshold (a.i.):	0.000	Tolerance (Da):	0.700	Number of Peaks:	53
Above Threshold:	53	Assigned Peaks:	28	Not assigned Peaks:	25		

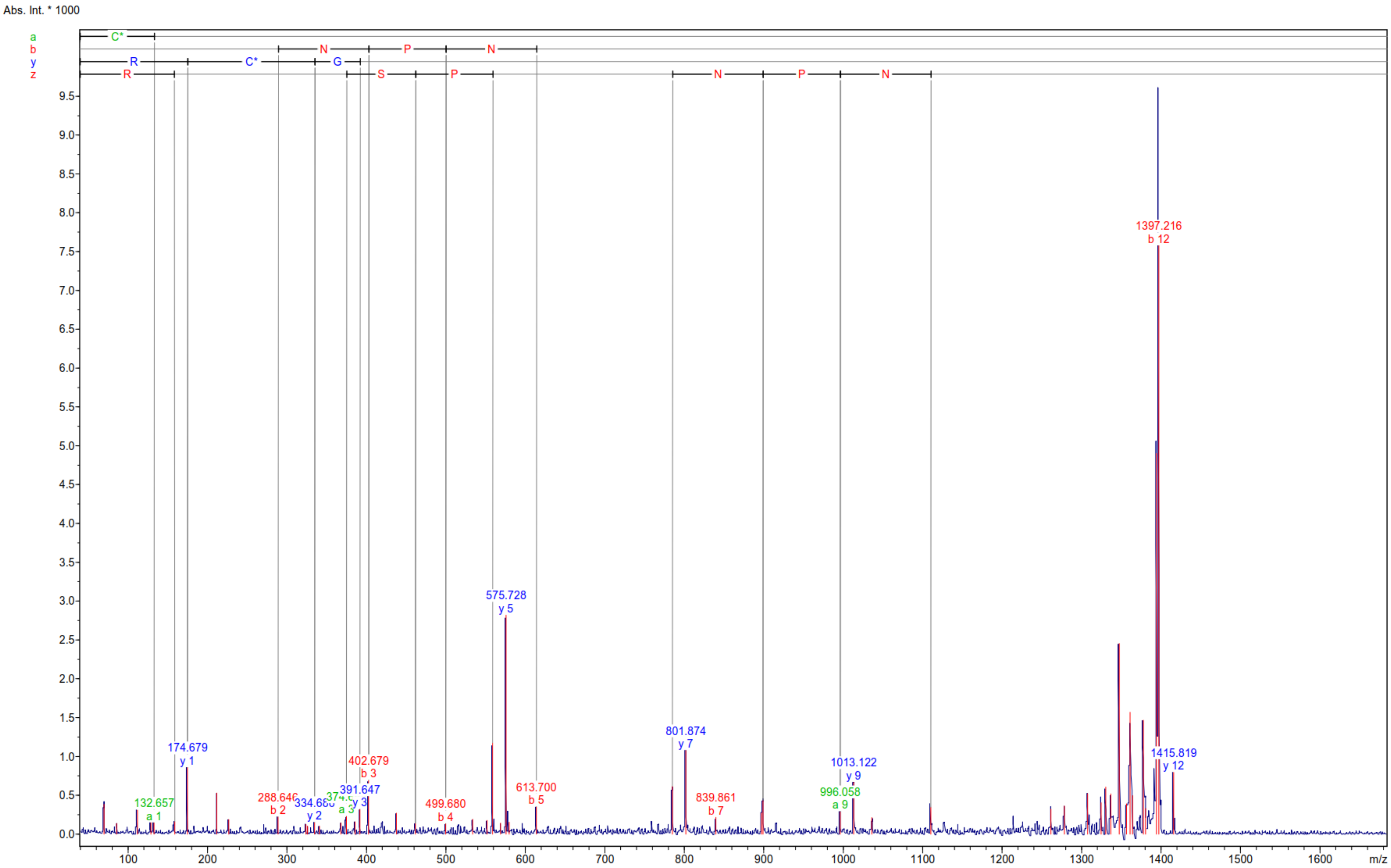

Copyright © 1999-2008 Bruker Daltonik GmbH

Spectrum Analysis Report

Display Parameter:

Parentmass:	1415.821	Mass Error:	0.205	MH+ (mono):	1415.616	MH+ (avg):	1416.568
Threshold (a.i.):	0.000	Tolerance (Da):	0.700	Number of Peaks:	53	Above Threshold:	53
Assigned Peaks:	28	Not assigned Peaks:	25				

Peaklist:

Peak	Mass	Intensity	Peak	Mass	Intensity	Peak	Mass	Intensity	Peak	Mass	Intensity	Peak	Mass	Intensity
1	69.783	385.724	2	85.715	137.374	3	111.689	312.410	4	128.700	231.975	5	132.657	149.088
6	158.658	163.609	7	174.679	861.909	8	211.653	529.954	9	226.648	190.542	10	288.646	224.137
11	323.699	126.889	12	325.659	103.924	13	334.686	155.279	14	340.644	102.816	15	367.696	147.624
16	374.658	231.271	17	385.647	162.008	18	391.647	322.251	19	402.679	694.671	20	437.700	271.705
21	461.614	138.860	22	499.680	136.857	23	533.714	191.822	24	551.751	177.763	25	558.704	1174.923
26	568.679	142.549	27	575.728	2822.048	28	579.624	154.576	29	613.700	355.785	30	784.843	615.231
31	801.874	1079.341	32	839.861	219.460	33	897.021	276.115	34	898.957	435.004	35	996.058	292.727
36	1013.122	669.989	37	1036.080	213.804	38	1110.147	344.835	39	1261.448	321.852	40	1278.288	362.147
41	1307.531	521.591	42	1324.579	406.852	43	1330.355	607.376	44	1336.659	520.079	45	1347.473	2455.322
46	1356.511	392.379	47	1361.107	1570.874	48	1364.177	496.463	49	1377.587	1468.881	50	1380.441	330.896
51	1394.166	4901.564	52	1397.216	7579.544	53	1415.819	798.953						

Calculated Masses:
CKNPNPEPSGCR 1: Carbamidomethyl (C) 11: Carbamidomethyl (C)

N-Term.	Ion	a	a-17	a-18	b	b-17	b-18	b+18	c	i	x	y	z	C-Term.	Ion
1	C*	**133.043**	116.016	115.032	161.038	144.011	143.027	179.048	178.064	**133.043**	201.098	**175.119**	**158.092**	12	R
2	K	261.138	244.111	243.127	**289.133**	272.106	271.122	307.143	306.159	101.107	361.129	**335.150**	318.123	11	C*
3	N	**375.181**	358.154	357.170	**403.176**	**386.149**	**385.165**	421.186	420.202	87.055	418.150	**392.171**	**375.145**	10	G
4	P	472.234	455.207	454.223	**500.229**	483.202	482.218	518.239	517.255	**70.065**	505.182	479.203	**462.177**	9	S
5	N	586.277	**569.250**	**568.266**	**614.272**	597.245	596.261	632.282	631.298	87.055	602.235	**576.256**	**559.229**	8	P
6	P	683.329	666.303	665.319	711.324	694.298	693.314	729.335	728.351	**70.065**	731.278	705.298	688.272	7	E
7	E	812.372	795.345	794.361	**840.367**	823.340	822.356	858.377	857.393	102.055	828.330	**802.351**	**785.325**	6	P
8	P	909.425	892.398	891.414	937.420	920.393	919.409	955.430	954.446	**70.065**	942.373	916.394	**899.368**	5	N
9	S	**996.457**	979.430	978.446	1024.452	1007.425	1006.441	1042.462	1041.478	60.044	1039.426	**1013.447**	**996.420**	4	P
10	G	1053.478	**1036.452**	**1035.468**	1081.473	1064.447	1063.463	1099.484	1098.500	30.034	1153.469	1127.490	**1110.463**	3	N
11	C*	1213.509	1196.482	1195.498	1241.504	1224.477	1223.493	1259.514	1258.530	**133.043**	1281.564	1255.585	1238.558	2	K
12	R	1369.610	1352.583	1351.599	**1397.605**	**1380.578**	1379.594	**1415.615**	1414.631	**129.113**	1441.595	**1415.615**	1398.589	1	C*

Copyright © 1999-2008 Bruker Daltonik GmbH

VNGF parentmass 962.627

Spectrum Analysis Report

Sequence Name:		Parentmass:	962.627	Mass Error:	0.164	MH+ (mono):	962.462
MH+ (avg):	963.066	Threshold (a.i.):	0.000	Tolerance (Da):	0.700	Number of Peaks:	40
Above Threshold:	40	Assigned Peaks:	27	Not assigned Peaks:	13		

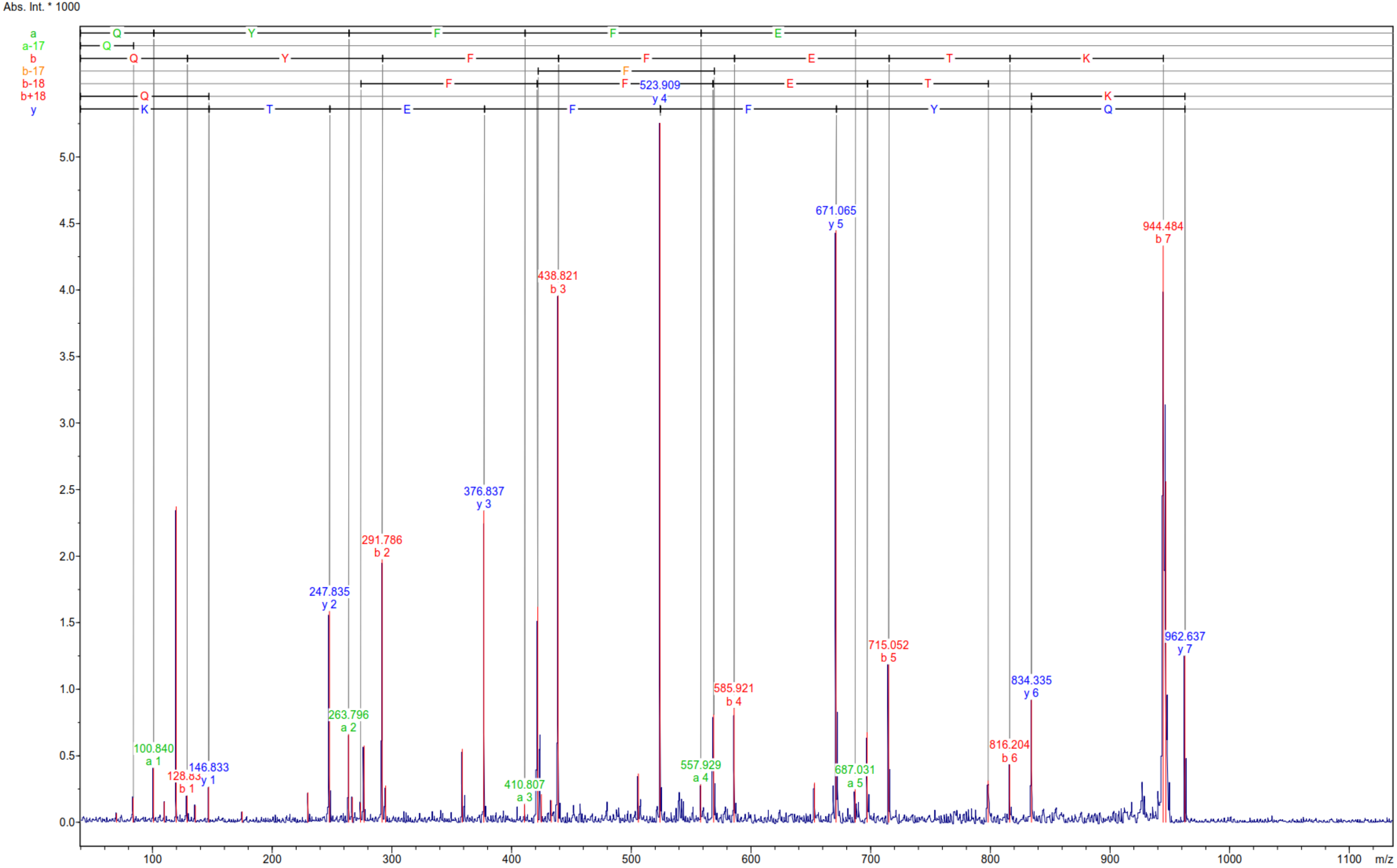

Copyright © 1999-2008 Bruker Daltonik GmbH

Spectrum Analysis Report

Display Parameter:

Parentmass:	962.627	Mass Error:	0.164	MH+ (mono):	962.462	MH+ (avg):	963.066
Threshold (a.i.):	0.000	Tolerance (Da):	0.700	Number of Peaks:	40	Above Threshold:	40
Assigned Peaks:	27	Not assigned Peaks:	13				

Peaklist:

Peak	Mass	Intensity	Peak	Mass	Intensity	Peak	Mass	Intensity	Peak	Mass	Intensity	Peak	Mass	Intensity
1	69.849	65.667	2	83.856	184.813	3	100.840	409.010	4	109.836	152.857	5	119.836	2371.761
6	128.838	200.649	7	135.816	125.041	8	146.833	266.892	9	174.840	79.989	10	229.821	223.193
11	247.835	1585.986	12	263.796	662.591	13	266.897	187.946	14	273.775	149.455	15	276.792	575.231
16	291.786	1974.939	17	294.800	274.451	18	358.819	549.471	19	376.837	2342.052	20	410.807	138.672
21	421.807	1621.130	22	424.817	209.274	23	433.140	164.150	24	438.821	3959.105	25	505.878	365.295
26	523.909	5391.345	27	557.929	286.228	28	568.903	811.209	29	585.921	858.940	30	653.024	296.832
31	671.065	4449.365	32	687.031	247.740	33	697.037	678.241	34	715.052	1186.543	35	798.169	313.725
36	816.204	435.130	37	834.335	919.998	38	944.484	4332.823	39	946.572	2560.058	40	962.637	1251.340

Calculated Masses:

QYFFETK

N-Term.	Ion	a	a-17	a-18	b	b-17	b-18	b+18	c	i	x	y	z	C-Term.	Ion
1	Q	**101.071**	**84.044**	83.060	**129.066**	112.039	111.055	**147.076**	146.092	**101.071**	173.092	**147.113**	130.086	7	K
2	Y	**264.134**	247.108	246.124	**292.129**	275.103	**274.119**	310.140	309.156	**136.076**	**274.140**	**248.160**	231.134	6	T
3	F	**411.203**	394.176	393.192	**439.198**	**422.171**	**421.187**	457.208	456.224	**120.081**	403.182	**377.203**	360.177	5	E
4	F	**558.271**	541.245	540.261	**586.266**	**569.239**	**568.255**	604.277	603.293	**120.081**	550.251	**524.271**	507.245	4	F
5	E	**687.314**	670.287	669.303	**715.309**	698.282	**697.298**	733.319	732.335	102.055	**697.319**	**671.340**	654.313	3	F
6	T	788.361	771.335	770.351	**816.356**	799.330	**798.346**	**834.367**	833.383	74.060	860.382	**834.403**	817.377	2	Y
7	K	916.456	899.430	898.446	**944.451**	927.425	926.441	**962.462**	961.478	**101.107**	988.441	**962.462**	945.435	1	Q

Copyright © 1999-2008 Bruker Daltonik GmbH

VNGF parentmass 1363.885

Spectrum Analysis Report

Sequence Name:		Parentmass:	1363.885	Mass Error:	0.242	MH+ (mono):	1363.643
MH+ (avg):	1364.511	Threshold (a.i.):	0.000	Tolerance (Da):	0.700	Number of Peaks:	99
Above Threshold:	99	Assigned Peaks:	44	Not assigned Peaks:	55		

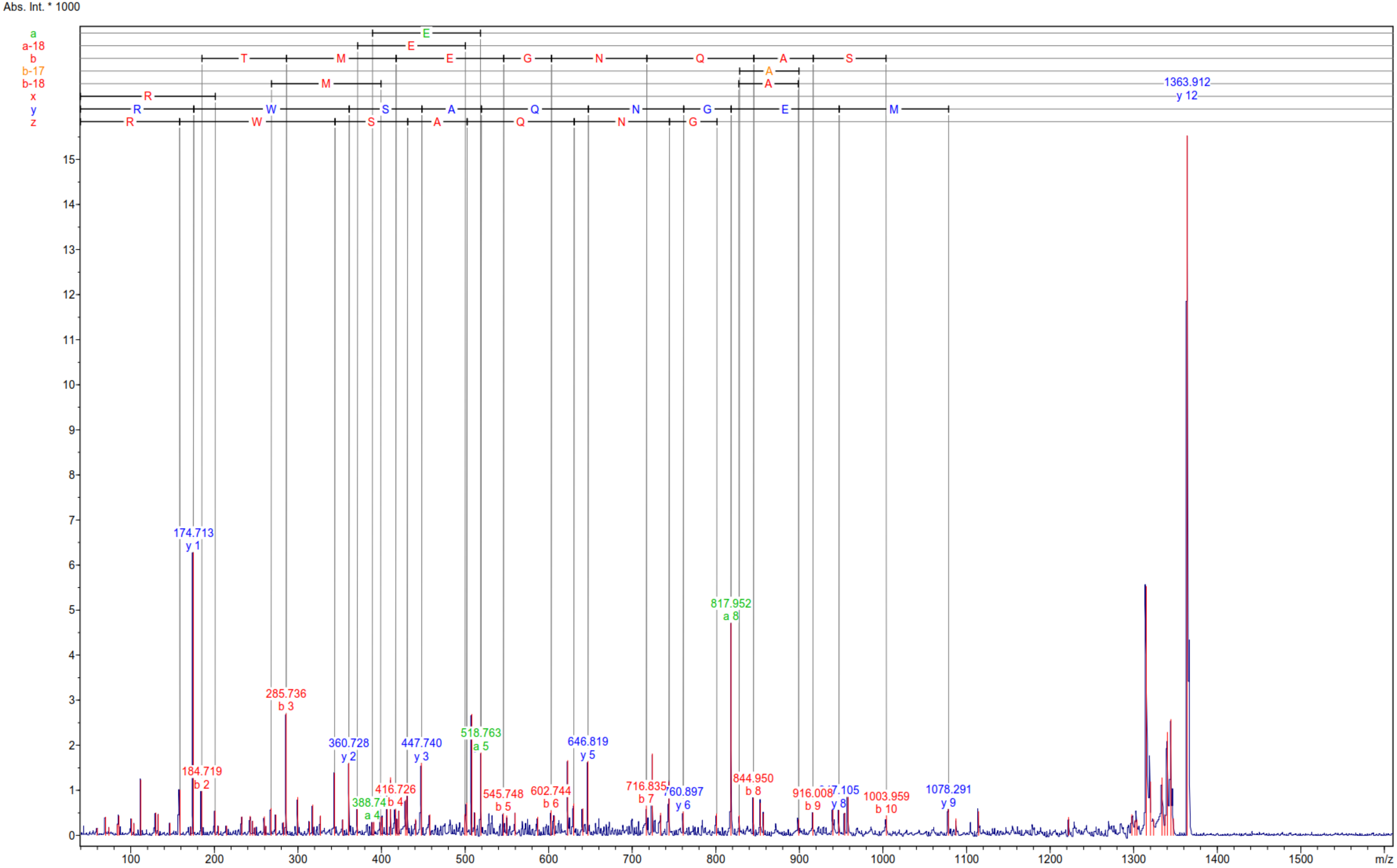

Copyright © 1999-2008 Bruker Daltonik GmbH

Spectrum Analysis Report

Display Parameter:

Parentmass:	1363.885	Mass Error:	0.242	MH+ (mono):	1363.643	MH+ (avg):	1364.511
Threshold (a.i.):	0.000	Tolerance (Da):	0.700	Number of Peaks:	99	Above Threshold:	99
Assigned Peaks:	44	Not assigned Peaks:	55				

Peaklist:

Peak	Mass	Intensity	Peak	Mass	Intensity	Peak	Mass	Intensity	Peak	Mass	Intensity	Peak	Mass	Intensity
1	59.755	154.898	2	69.749	408.953	3	73.749	177.308	4	83.725	237.192	5	85.777	432.796
6	100.722	367.575	7	103.722	260.742	8	111.727	1231.692	9	129.705	501.376	10	132.686	464.074
11	146.719	269.422	12	158.699	1020.170	13	171.679	185.367	14	174.713	6287.204	15	184.719	984.628
16	200.676	544.351	17	204.685	205.052	18	214.687	211.494	19	232.699	413.387	20	242.688	424.485
21	245.699	317.940	22	248.690	172.774	23	259.753	415.796	24	261.668	203.298	25	267.713	610.157
26	273.683	456.056	27	282.669	290.072	28	285.736	2715.039	29	299.688	846.245	30	313.701	301.440
31	317.687	677.067	32	326.686	434.654	33	343.708	1391.046	34	353.675	346.124	35	360.728	1607.308
36	370.720	756.351	37	388.741	292.983	38	390.651	333.995	39	398.743	415.889	40	400.739	399.485
41	406.782	1007.369	42	410.672	1284.913	43	416.726	588.453	44	418.695	355.081	45	420.709	540.792
46	428.697	785.966	47	430.715	1140.125	48	440.704	356.741	49	447.740	1607.475	50	457.709	462.334
51	499.729	476.076	52	501.736	727.610	53	507.802	2686.618	54	511.683	498.577	55	518.763	1839.571
56	545.748	488.816	57	549.706	434.554	58	559.735	497.346	59	586.796	407.344	60	602.744	558.673
61	606.688	427.853	62	622.782	1660.915	63	629.776	710.941	64	640.733	594.906	65	646.819	1646.801
66	716.835	659.923	67	723.872	1811.374	68	733.822	495.415	69	743.857	1196.180	70	760.897	529.308
71	800.874	484.194	72	817.952	4718.366	73	827.924	450.692	74	844.950	839.740	75	852.987	717.003
76	856.849	485.840	77	899.022	356.707	78	916.008	512.600	79	940.006	640.633	80	947.105	564.602
81	954.200	493.025	82	957.996	941.137	83	1003.959	442.508	84	1078.291	584.060	85	1087.192	370.295
86	1114.424	531.190	87	1221.875	403.858	88	1297.707	462.525	89	1301.385	330.754	90	1303.653	341.883
91	1314.918	5526.804	92	1319.943	1188.828	93	1323.720	356.031	94	1333.752	1277.820	95	1336.915	461.855
96	1340.095	2290.538	97	1344.379	2575.757	98	1347.339	374.142	99	1363.912	16283.677			

Calculated Masses:

ALTMEGNQASWR

N-Term.	Ion	a	a-17	a-18	b	b-17	b-18	b+18	c	i	x	y	z	C-Term.	Ion
1	A	44.049	27.023	26.039	72.044	55.018	54.034	90.055	89.071	44.049	**201.098**	**175.119**	**158.092**	12	R
2	L	157.134	140.107	139.123	**185.128**	168.102	167.118	203.139	202.155	**86.096**	387.178	**361.198**	**344.172**	11	W
3	T	258.181	241.155	240.171	**286.176**	269.150	**268.166**	304.187	303.203	**74.060**	474.210	**448.230**	**431.204**	10	S
4	M	**389.222**	372.195	**371.211**	**417.217**	**400.190**	**399.206**	435.227	434.243	**104.053**	**545.247**	**519.267**	**502.241**	9	A
5	E	**518.264**	**501.238**	**500.254**	**546.259**	529.233	528.249	564.270	563.286	102.055	673.305	**647.326**	**630.299**	8	Q
6	G	575.286	558.259	557.275	**603.281**	**586.254**	585.270	621.291	620.307	30.034	787.348	**761.369**	**744.342**	7	N
7	N	689.329	672.302	671.318	**717.324**	700.297	699.313	735.334	**734.350**	87.055	**844.370**	**818.390**	**801.364**	6	G
8	Q	**817.387**	**800.361**	799.377	**845.382**	**828.356**	**827.372**	863.393	862.409	**101.071**	973.412	**947.433**	930.406	5	E
9	A	888.424	871.398	870.414	**916.419**	**899.393**	**898.409**	934.430	933.446	44.049	1104.453	**1078.473**	1061.447	4	M
10	S	975.456	**958.430**	**957.446**	**1003.451**	986.425	985.441	1021.462	1020.478	**60.044**	1205.500	1179.521	1162.495	3	T
11	W	1161.536	1144.509	1143.525	1189.531	1172.504	1171.520	1207.541	1206.557	**159.092**	1318.584	1292.605	1275.579	2	L
12	R	1317.637	1300.610	1299.626	1345.632	1328.605	1327.621	**1363.642**	1362.658	**129.113**	1389.622	**1363.642**	1346.616	1	A

Copyright © 1999-2008 Bruker Daltonik GmbH

VNGF parentmass 379.914

Spectrum Analysis Report

Sequence Name:		Parentmass:	1379.914	Mass Error:	0.277	MH+ (mono):	1379.638
MH+ (avg):	1380.510	Threshold (a.i.):	0.000	Tolerance (Da):	0.700	Number of Peaks:	90
Above Threshold:	90	Assigned Peaks:	34	Not assigned Peaks:	56		

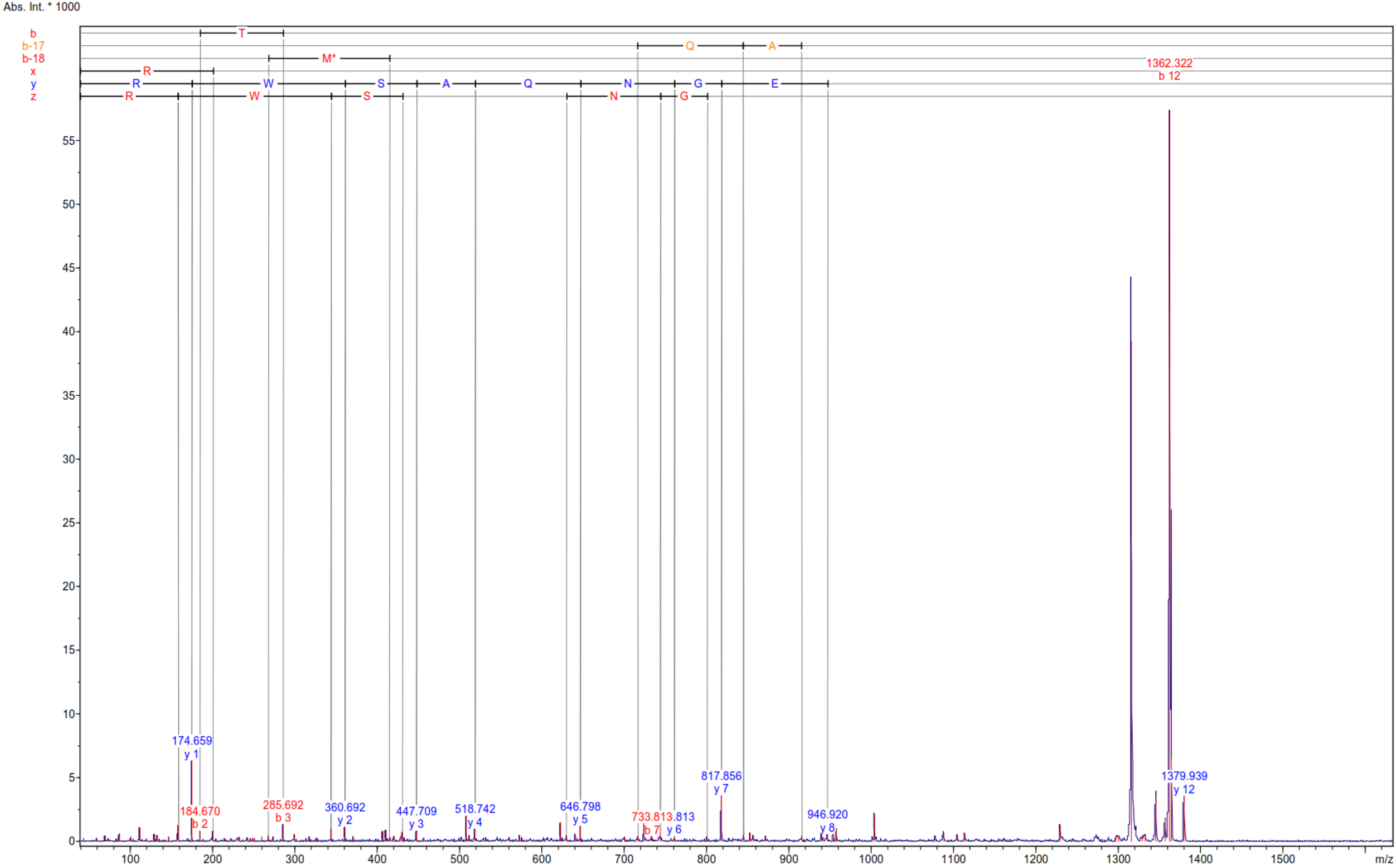

Copyright © 1999-2008 Bruker Daltonik GmbH

Spectrum Analysis Report

Display Parameter:

Parentmass:	1379.914	Mass Error:	0.277	MH+ (mono):	1379.638	MH+ (avg):	1380.510
Threshold (a.i.):	0.000	Tolerance (Da):	0.700	Number of Peaks:	90	Above Threshold:	90
Assigned Peaks:	34	Not assigned Peaks:	56				

Peaklist:

Peak	Mass	Intensity	Peak	Mass	Intensity	Peak	Mass	Intensity	Peak	Mass	Intensity	Peak	Mass	Intensity
1	59.753	234.888	2	69.707	431.089	3	73.728	223.213	4	83.675	197.758	5	86.707	587.942
6	100.666	337.872	7	103.655	161.204	8	111.674	1058.664	9	119.678	192.273	10	129.672	535.452
11	132.619	481.771	12	135.629	165.587	13	146.659	338.051	14	158.651	1243.681	15	169.631	208.327
16	174.659	6344.863	17	184.670	830.065	18	188.638	146.813	19	200.626	813.705	20	204.647	197.834
21	214.645	232.619	22	222.626	200.575	23	230.621	239.032	24	232.636	329.828	25	242.629	295.583
26	245.630	205.354	27	248.643	232.634	28	250.623	228.530	29	259.712	359.841	30	267.685	462.024
31	273.648	374.310	32	285.692	1347.184	33	299.650	573.080	34	313.658	246.028	35	317.646	372.277
36	326.661	269.251	37	343.672	1013.881	38	360.692	1164.816	39	370.686	399.272	40	406.761	808.216
41	410.623	898.653	42	414.678	259.124	43	420.659	412.558	44	428.691	434.554	45	430.684	758.374
46	447.709	841.407	47	455.671	260.083	48	507.803	2317.344	49	511.676	463.694	50	518.742	977.635
51	560.696	308.524	52	572.728	486.174	53	575.673	311.613	54	622.792	1493.390	55	629.778	499.935
56	640.747	574.744	57	646.798	1232.216	58	700.729	370.967	59	716.817	432.937	60	723.832	1776.491
61	733.813	400.730	62	743.820	954.852	63	760.813	402.738	64	800.783	406.190	65	817.856	3601.916
66	844.846	555.016	67	852.855	672.320	68	856.754	491.604	69	871.823	439.102	70	915.829	406.400
71	939.771	740.499	72	946.920	572.721	73	953.851	595.929	74	957.752	1006.548	75	1004.079	2082.750
76	1077.889	407.951	77	1088.037	643.929	78	1104.725	482.352	79	1113.673	618.638	80	1229.617	1311.033
81	1297.406	428.935	82	1300.475	466.365	83	1315.891	39261.850	84	1329.066	484.924	85	1332.509	491.629
86	1345.568	3475.931	87	1356.987	1660.047	88	1362.322	59536.639	89	1364.564	6457.740	90	1379.939	3590.383

Calculated Masses:
ALTMEGNQASWR 4: Oxidation (M)

N-Term.	Ion	a	a-17	a-18	b	b-17	b-18	b+18	c	i	x	y	z	C-Term.	Ion
1	A	44.049	27.023	26.039	72.044	55.018	54.034	90.055	89.071	44.049	**201.098**	**175.119**	**158.092**	12	R
2	L	157.134	140.107	139.123	**185.128**	168.102	167.118	203.139	202.155	**86.096**	387.178	**361.198**	**344.172**	11	W
3	T	258.181	241.155	240.171	**286.176**	269.150	**268.166**	304.187	303.203	**74.060**	474.210	**448.230**	**431.204**	10	S
4	M*	405.217	388.190	387.206	433.212	416.185	**415.201**	451.222	450.238	**120.048**	545.247	**519.267**	502.241	9	A
5	E	534.259	517.233	516.249	562.254	545.228	544.244	580.265	579.281	102.055	673.305	**647.326**	**630.299**	8	Q
6	G	591.281	574.254	**573.270**	619.276	602.249	601.265	637.286	636.302	30.034	787.348	**761.369**	**744.342**	7	N
7	N	705.324	688.297	687.313	**733.319**	**716.292**	715.308	751.329	750.345	**87.055**	**844.370**	**818.390**	**801.364**	6	G
8	Q	833.382	816.356	815.372	861.377	**844.351**	843.367	879.388	878.404	**101.071**	973.412	**947.433**	930.406	5	E
9	A	904.419	887.393	886.409	932.414	**915.388**	914.404	950.425	949.441	44.049	1120.448	1094.468	**1077.442**	4	M*
10	S	991.451	974.425	973.441	1019.446	1002.420	1001.436	1037.457	1036.473	**60.044**	1221.495	1195.516	1178.489	3	T
11	W	1177.531	1160.504	1159.520	1205.526	1188.499	1187.515	1223.536	1222.552	**159.092**	1334.579	1308.600	1291.574	2	L
12	R	1333.632	1316.605	**1315.621**	**1361.627**	1344.600	1343.616	**1379.637**	1378.653	**129.113**	1405.616	**1379.637**	**1362.611**	1	A

Copyright © 1999-2008 Bruker Daltonik GmbH

3FTx parentmass 1302.807

Spectrum Analysis Report

Sequence Name:		Parentmass:	1302.807	Mass Error:	0.140	MH+ (mono):	1302.667
MH+ (avg):	1303.555	Threshold (a.i.):	0.000	Tolerance (Da):	0.700	Number of Peaks:	54
Above Threshold:	54	Assigned Peaks:	34	Not assigned Peaks:	20		

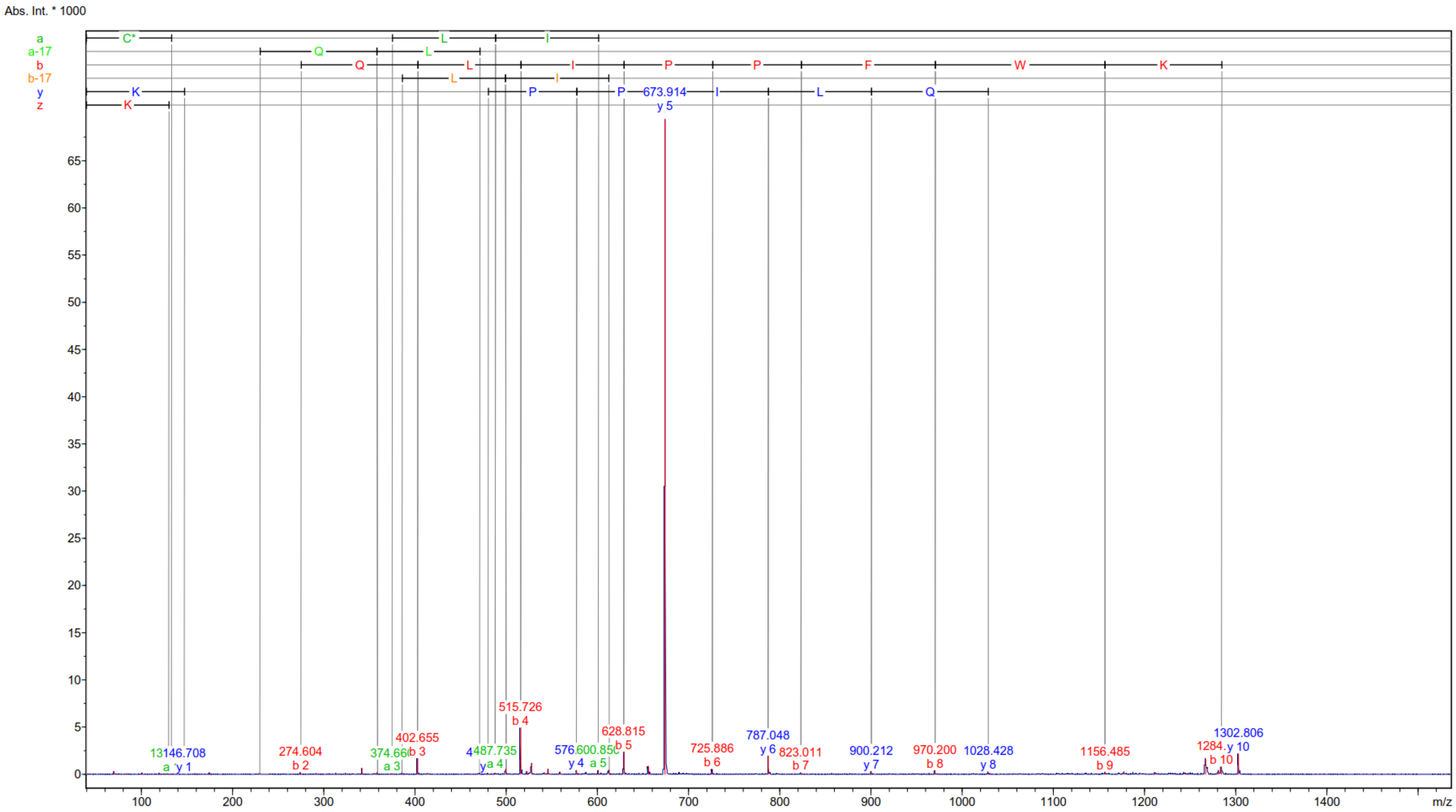

Copyright © 1999-2008 Bruker Daltonik GmbH

Spectrum Analysis Report

Display Parameter:

Parentmass:	1302.807	Mass Error:	0.140	MH+ (mono):	1302.667	MH+ (avg):	1303.555
Threshold (a.i.):	0.000	Tolerance (Da):	0.700	Number of Peaks:	54	Above Threshold:	54
Assigned Peaks:	34	Not assigned Peaks:	20				

Peaklist:

Peak	Mass	Intensity	Peak	Mass	Intensity	Peak	Mass	Intensity	Peak	Mass	Intensity	Peak	Mass	Intensity
1	69.755	291.205	2	83.707	76.950	3	100.734	142.313	4	119.685	111.635	5	125.673	52.285
6	129.672	128.983	7	132.671	64.678	8	146.708	57.730	9	158.697	78.669	10	174.686	161.095
11	229.620	83.155	12	274.604	203.376	13	291.684	77.309	14	306.687	73.347	15	313.735	106.003
16	323.671	104.895	17	341.684	640.027	18	355.706	93.162	19	358.712	175.740	20	374.660	96.246
21	385.664	178.751	22	402.655	1707.760	23	413.671	114.938	24	470.748	134.801	25	479.791	147.972
26	487.735	346.735	27	499.762	599.767	28	515.726	4932.536	29	522.815	316.984	30	527.742	1095.929
31	541.956	174.918	32	545.792	536.729	33	558.725	234.355	34	576.833	449.419	35	600.850	430.786
36	612.829	441.751	37	628.815	2393.001	38	655.890	857.043	39	673.914	70136.875	40	725.886	565.651
41	787.048	1974.516	42	823.011	193.415	43	900.212	327.368	44	970.200	420.351	45	1028.428	270.838
46	1156.485	262.403	47	1176.898	229.067	48	1211.272	205.371	49	1243.506	192.627	50	1266.704	1537.940
51	1269.777	200.859	52	1281.070	382.895	53	1284.186	796.225	54	1302.806	2180.585			

Calculated Masses:

CNQLIPPFWK 1: Carbamidomethyl (C)

N-Term.	Ion	a	a-17	a-18	b	b-17	b-18	b+18	c	i	x	y	z	C-Term.	Ion
1	C*	**133.043**	116.016	115.032	161.038	144.011	143.027	179.048	178.064	**133.043**	173.092	**147.113**	**130.086**	10	K
2	N	247.086	**230.059**	**229.075**	**275.081**	258.054	257.070	293.091	**292.107**	87.055	**359.171**	333.192	316.166	9	W
3	Q	**375.145**	**358.118**	357.134	**403.139**	**386.113**	**385.129**	421.150	420.166	**101.071**	506.240	**480.261**	463.234	8	F
4	L	**488.229**	**471.202**	**470.218**	**516.223**	**499.197**	498.213	534.234	533.250	86.096	603.293	**577.313**	560.287	7	P
5	I	**601.313**	584.286	583.302	**629.308**	**612.281**	611.297	647.318	646.334	86.096	700.345	**674.366**	657.340	6	P
6	P	698.365	681.339	680.355	**726.360**	709.334	708.350	744.371	743.387	**70.065**	813.429	**787.450**	770.424	5	I
7	P	795.418	778.392	777.408	**823.413**	806.387	805.403	841.424	840.440	**70.065**	926.513	**900.534**	883.508	4	L
8	F	942.487	925.460	924.476	**970.481**	953.455	952.471	988.492	987.508	**120.081**	1054.572	**1028.593**	1011.566	3	Q
9	W	1128.566	1111.539	1110.555	**1156.561**	1139.534	1138.550	1174.571	1173.587	**159.092**	1168.615	1142.636	1125.609	2	N
10	K	1256.661	1239.634	1238.650	**1284.656**	1267.629	**1266.645**	**1302.666**	1301.682	**101.107**	1328.646	**1302.666**	1285.640	1	C*

Copyright © 1999-2008 Bruker Daltonik GmbH

3FTx parentmass 1091.463

Spectrum Analysis Report

Sequence Name:		Parentmass:	1091.463	Mass Error:	0.067	MH+ (mono):	1091.396
MH+ (avg):	1092.253	Threshold (a.i.):	0.000	Tolerance (Da):	0.700	Number of Peaks:	46
Above Threshold:	46	Assigned Peaks:	18	Not assigned Peaks:	28		

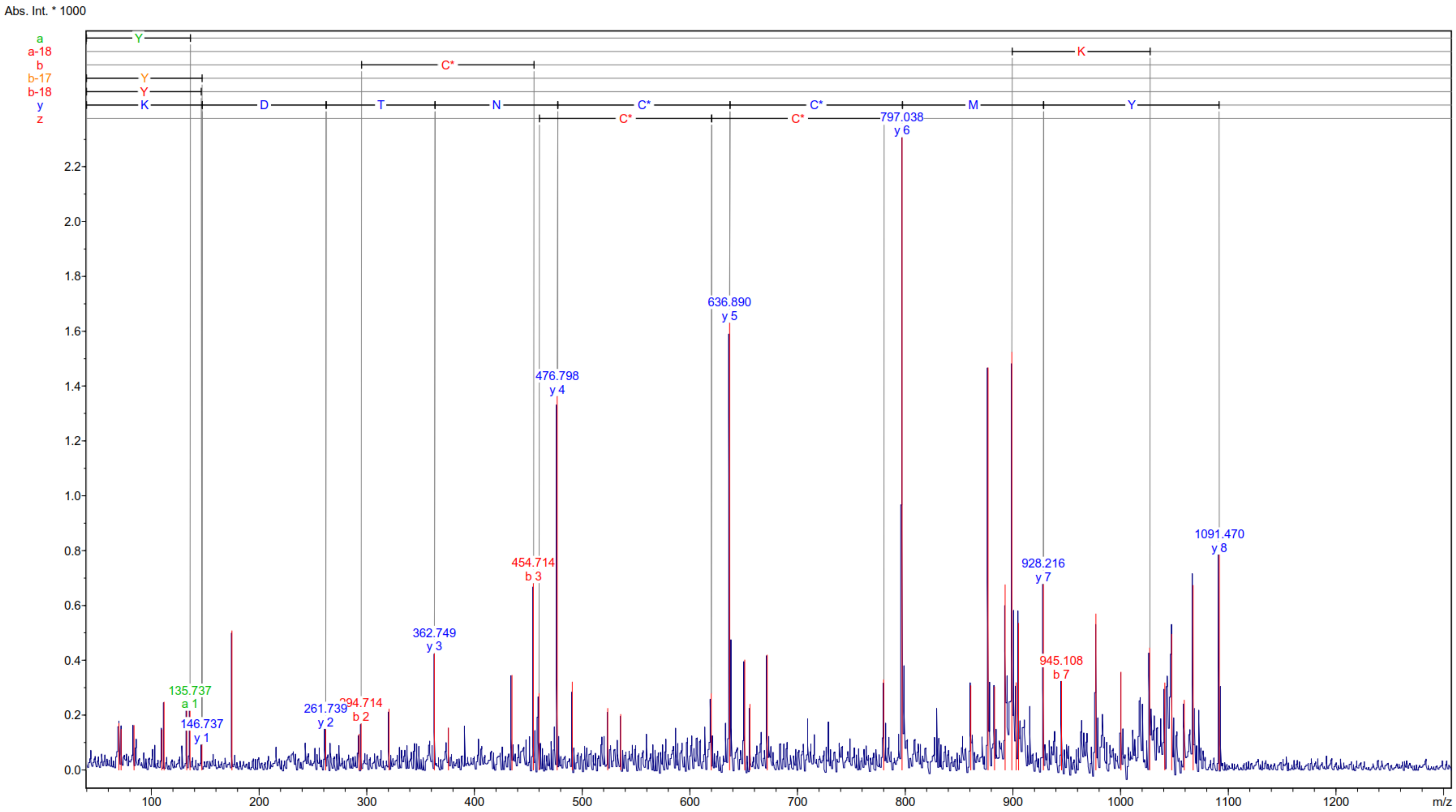

Copyright © 1999-2008 Bruker Daltonik GmbH

Spectrum Analysis Report

Display Parameter:

Parentmass:	1091.463	Mass Error:	0.067	MH+ (mono):	1091.396	MH+ (avg):	1092.253
Threshold (a.i.):	0.000	Tolerance (Da):	0.700	Number of Peaks:	46	Above Threshold:	46
Assigned Peaks:	18	Not assigned Peaks:	28				

Peaklist:

Peak	Mass	Intensity	Peak	Mass	Intensity	Peak	Mass	Intensity	Peak	Mass	Intensity	Peak	Mass	Intensity
1	69.799	169.470	2	71.789	148.589	3	83.795	162.922	4	109.715	147.007	5	111.747	248.214
6	132.713	266.764	7	135.737	215.294	8	146.737	93.497	9	174.741	507.808	10	261.739	148.937
11	292.688	130.228	12	294.714	169.864	13	320.655	222.830	14	362.749	426.180	15	375.737	153.377
16	434.671	345.832	17	454.714	681.384	18	459.798	279.757	19	476.798	1363.040	20	490.723	320.870
21	523.757	225.132	22	535.776	203.033	23	619.834	279.820	24	636.890	1630.727	25	650.813	401.717
26	655.783	240.353	27	671.721	419.344	28	779.986	330.119	29	797.038	2307.387	30	860.790	307.427
31	876.865	1467.290	32	882.891	304.013	33	892.763	675.551	34	898.851	1524.235	35	902.940	318.814
36	905.084	535.242	37	928.216	677.936	38	945.108	324.530	39	977.055	569.807	40	1000.339	357.250
41	1027.045	445.289	42	1040.965	317.907	43	1047.367	495.236	44	1059.021	254.918	45	1067.204	673.158
46	1091.470	784.948												

Calculated Masses:

YMCCNTDK 3: Carbamidomethyl (C) 4: Carbamidomethyl (C)

N-Term.	Ion	a	a-17	a-18	b	b-17	b-18	b+18	c	i	x	y	z	C-Term.	Ion
1	Y	**136.076**	119.049	118.065	164.071	**147.044**	**146.060**	182.081	181.097	**136.076**	173.092	**147.113**	130.086	8	K
2	M	267.116	250.090	249.106	**295.111**	278.085	277.101	313.122	312.138	104.053	288.119	**262.140**	245.113	7	D
3	C*	427.147	410.120	409.136	**455.142**	438.115	437.131	473.152	472.168	**133.043**	389.167	**363.187**	346.161	6	T
4	C*	587.177	570.151	569.167	615.172	598.146	597.162	633.183	632.199	**133.043**	503.210	**477.230**	**460.204**	5	N
5	N	701.220	684.194	683.210	729.215	712.189	711.205	747.226	746.242	87.055	663.240	**637.261**	**620.234**	4	C*
6	T	802.268	785.242	784.258	830.263	813.236	812.252	848.274	847.290	74.060	823.271	**797.292**	**780.265**	3	C*
7	D	917.295	900.268	**899.284**	**945.290**	**928.263**	927.279	963.300	962.316	88.039	954.311	**928.332**	911.306	2	M
8	K	1045.390	1028.363	**1027.379**	1073.385	1056.358	1055.374	**1091.395**	1090.411	101.107	1117.375	**1091.395**	1074.369	1	Y

Copyright © 1999-2008 Bruker Daltonik GmbH

3FTx parentmass 948.463

Spectrum Analysis Report

Sequence Name:		Parentmass:	948.463	Mass Error:	0.034	MH+ (mono):	948.428
MH+ (avg):	949.131	Threshold (a.i.):	0.000	Tolerance (Da):	0.700	Number of Peaks:	39
Above Threshold:	39	Assigned Peaks:	19	Not assigned Peaks:	20		

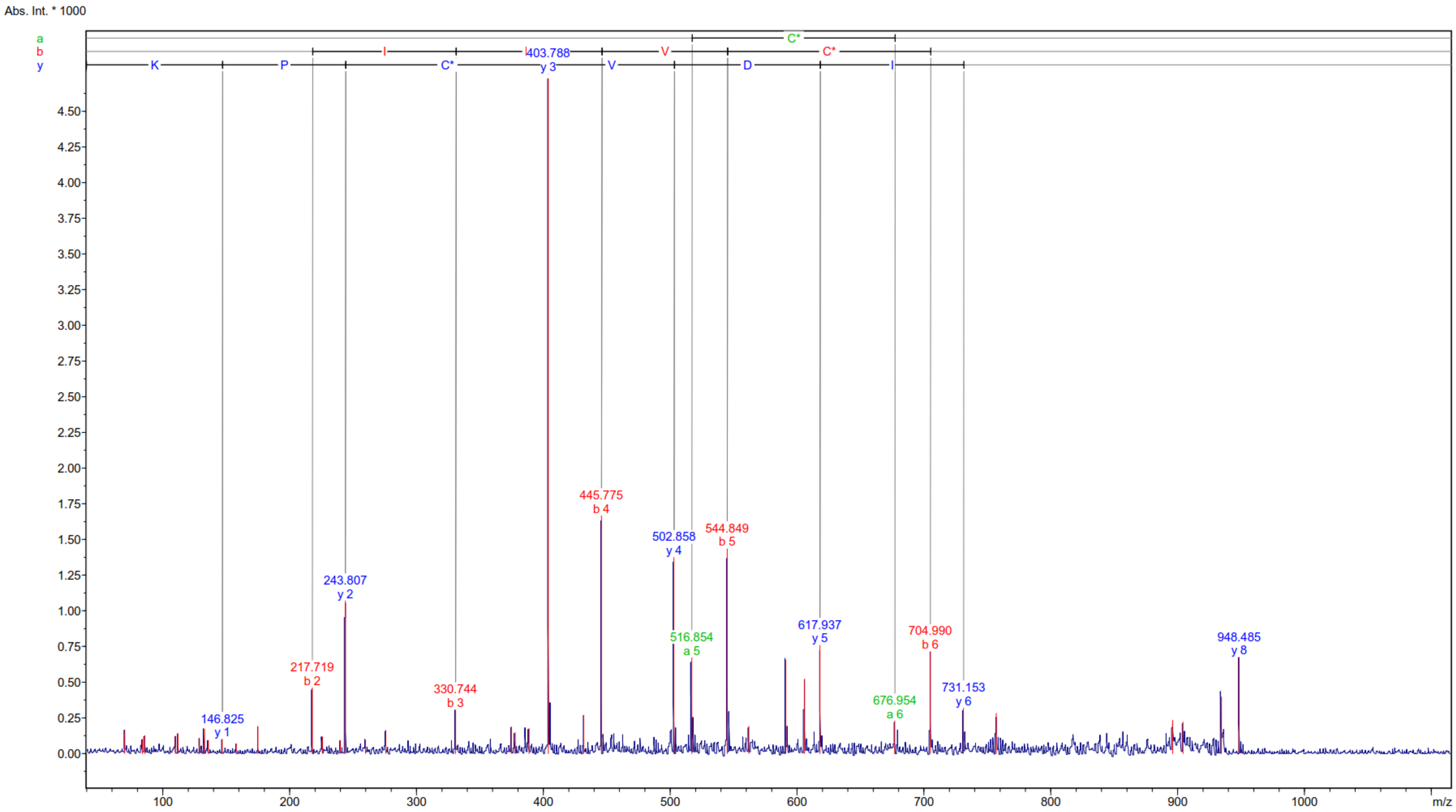

Copyright © 1999-2008 Bruker Daltonik GmbH

Spectrum Analysis Report

Display Parameter:

Parentmass:	948.463	Mass Error:	0.034	MH+ (mono):	948.428	MH+ (avg):	949.131
Threshold (a.i.):	0.000	Tolerance (Da):	0.700	Number of Peaks:	39	Above Threshold:	39
Assigned Peaks:	19	Not assigned Peaks:	20				

Peaklist:

Peak	Mass	Intensity	Peak	Mass	Intensity	Peak	Mass	Intensity	Peak	Mass	Intensity	Peak	Mass	Intensity
1	69.860	154.388	2	83.861	100.544	3	85.852	126.166	4	109.805	121.565	5	111.842	139.381
6	128.818	105.802	7	132.773	174.013	8	135.800	93.532	9	146.825	100.097	10	157.787	68.285
11	174.808	190.089	12	217.719	462.413	13	225.821	117.678	14	239.833	93.868	15	243.807	1071.082
16	259.756	94.876	17	275.771	164.991	18	330.744	306.964	19	374.748	188.738	20	377.692	148.129
21	388.742	176.835	22	403.788	4764.331	23	431.749	264.298	24	445.775	1668.025	25	502.858	1373.920
26	516.854	673.048	27	544.849	1436.327	28	561.847	192.147	29	591.018	656.156	30	605.864	522.383
31	617.937	758.875	32	676.954	228.938	33	704.990	715.761	34	731.153	319.281	35	757.003	282.325
36	896.050	234.152	37	904.186	222.056	38	934.447	396.939	39	948.485	673.669			

Calculated Masses:

GCIDVCPK 2: Carbamidomethyl (C) 6: Carbamidomethyl (C)

N-Term.	Ion	a	a-17	a-18	b	b-17	b-18	b+18	c	i	x	y	z	C-Term.	Ion
1	G	30.034	13.007	12.023	58.029	41.002	40.018	76.039	75.055	30.034	173.092	**147.113**	130.086	8	K
2	C*	190.064	173.038	172.054	**218.059**	201.033	200.049	236.070	235.086	**133.043**	270.145	**244.166**	227.139	7	P
3	I	303.149	286.122	285.138	**331.143**	314.117	313.133	349.154	348.170	**86.096**	430.175	**404.196**	387.170	6	C*
4	D	418.175	401.149	400.165	**446.170**	429.144	428.160	464.181	463.197	88.039	529.244	**503.265**	486.238	5	V
5	V	**517.244**	500.217	499.233	**545.239**	528.212	527.228	563.249	**562.265**	72.081	644.271	**618.292**	601.265	4	D
6	C*	**677.275**	660.248	659.264	**705.269**	688.243	687.259	723.280	722.296	**133.043**	**757.355**	**731.376**	714.349	3	I
7	P	774.327	**757.301**	**756.317**	802.322	785.296	784.312	820.333	819.349	**70.065**	917.386	891.406	874.380	2	C*
8	K	902.422	885.396	884.412	930.417	913.391	912.407	**948.428**	947.444	101.107	974.407	**948.428**	931.401	1	G

Copyright © 1999-2008 Bruker Daltonik GmbH

3FTx parentmass 1060.609

Spectrum Analysis Report

Sequence Name:		Parentmass:	1060.609	Mass Error:	0.189	MH+ (mono):	1060.419
MH+ (avg):	1061.175	Threshold (a.i.):	0.000	Tolerance (Da):	0.700	Number of Peaks:	55
Above Threshold:	55	Assigned Peaks:	24	Not assigned Peaks:	31		

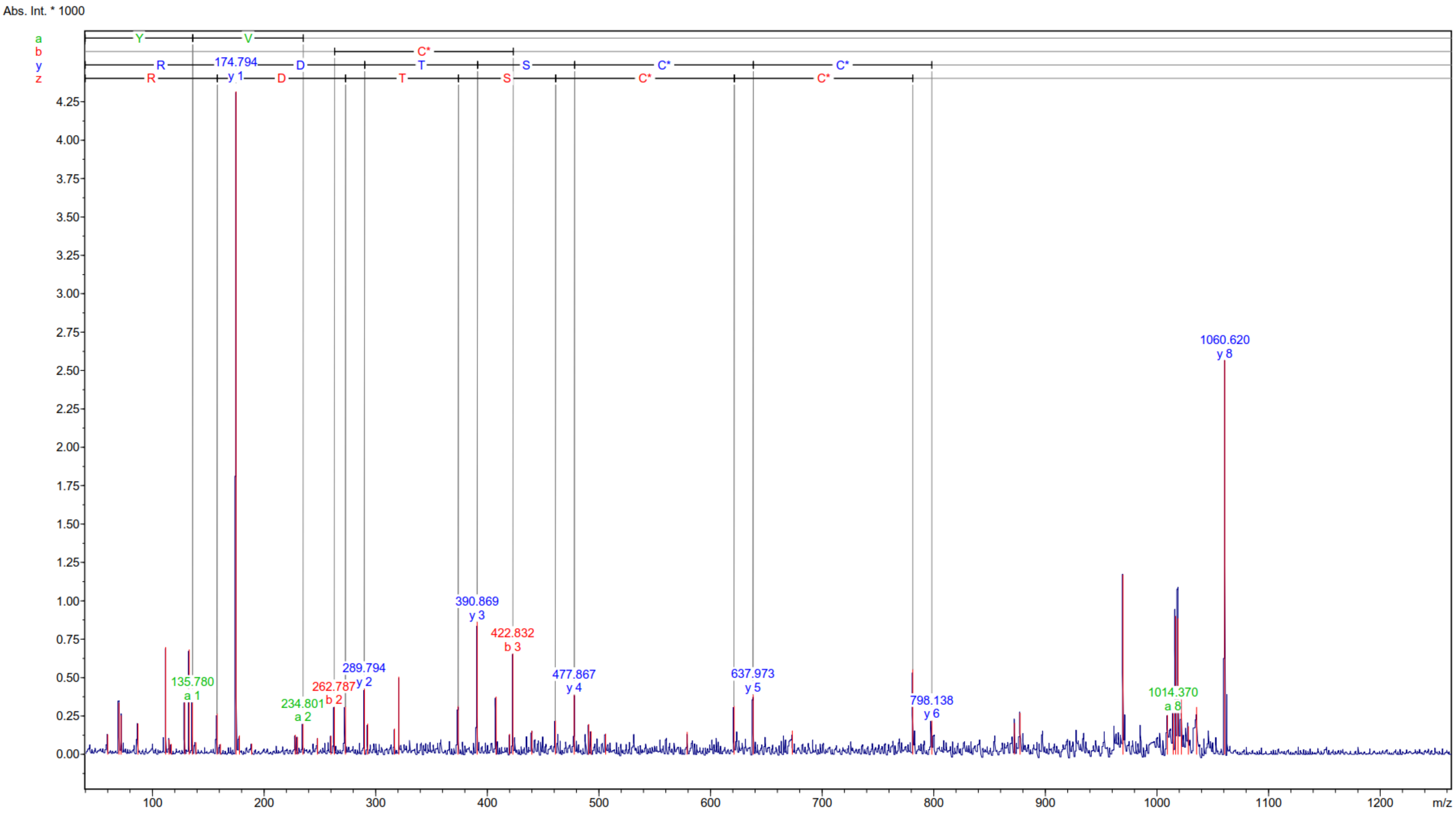

Copyright © 1999-2008 Bruker Daltonik GmbH

Spectrum Analysis Report

Display Parameter:

Parentmass:	1060.609	Mass Error:	0.189	MH+ (mono):	1060.419	MH+ (avg):	1061.175
Threshold (a.i.):	0.000	Tolerance (Da):	0.700	Number of Peaks:	55	Above Threshold:	55
Assigned Peaks:	24	Not assigned Peaks:	31				

Peaklist:

Peak	Mass	Intensity	Peak	Mass	Intensity	Peak	Mass	Intensity	Peak	Mass	Intensity	Peak	Mass	Intensity
1	59.825	128.889	2	69.831	334.017	3	71.842	251.479	4	86.841	199.911	5	111.814	697.197
6	114.791	96.946	7	116.834	64.977	8	128.809	336.610	9	132.753	680.984	10	135.780	337.961
11	138.760	76.719	12	157.777	263.592	13	160.736	65.463	14	174.794	4374.412	15	177.769	121.544
16	188.754	67.896	17	227.778	125.799	18	229.736	110.110	19	234.801	198.562	20	247.760	102.893
21	259.781	118.671	22	262.787	309.744	23	272.766	310.083	24	289.794	428.417	25	292.729	198.671
26	316.738	165.415	27	320.721	503.291	28	373.820	314.522	29	390.869	864.113	30	407.765	374.259
31	419.800	131.249	32	422.832	656.285	33	439.869	150.791	34	460.866	220.209	35	477.867	388.598
36	490.824	194.732	37	492.829	149.288	38	505.931	132.345	39	578.904	145.086	40	620.934	314.761
41	637.973	389.948	42	673.115	152.727	43	781.109	552.652	44	798.138	219.322	45	872.246	201.526
46	877.211	268.468	47	969.470	1168.553	48	1009.167	254.906	49	1014.370	268.041	50	1016.585	899.596
51	1018.739	883.555	52	1021.855	387.639	53	1028.007	181.682	54	1035.261	306.428	55	1060.620	2567.401

Calculated Masses:

YVCCSTDR 3: Carbamidomethyl (C) 4: Carbamidomethyl (C)

N-Term.	Ion	a	a-17	a-18	b	b-17	b-18	b+18	c	i	x	y	z	C-Term.	Ion
1	Y	**136.076**	119.049	118.065	164.071	147.044	146.060	182.081	181.097	**136.076**	201.098	**175.119**	**158.092**	8	R
2	V	**235.144**	218.118	217.134	**263.139**	246.112	245.128	281.150	280.166	**72.081**	**316.125**	**290.146**	**273.119**	7	D
3	C*	395.175	378.148	377.164	**423.170**	406.143	405.159	441.180	**440.196**	**133.043**	417.173	**391.194**	**374.167**	6	T
4	C*	555.205	538.179	537.195	583.200	566.174	565.190	601.211	600.227	**133.043**	504.205	**478.226**	**461.199**	5	S
5	S	642.237	625.211	624.227	670.232	653.206	652.222	688.243	687.259	**60.044**	664.236	**638.256**	**621.230**	4	C*
6	T	743.285	726.259	725.275	771.280	754.253	753.269	789.291	788.307	74.060	824.266	**798.287**	**781.260**	3	C*
7	D	858.312	841.286	840.301	886.307	869.280	868.296	904.318	903.334	88.039	923.335	897.355	880.329	2	V
8	R	**1014.413**	997.387	996.403	1042.408	1025.382	1024.398	**1060.419**	1059.435	**129.113**	1086.398	**1060.419**	1043.392	1	Y

Copyright © 1999-2008 Bruker Daltonik GmbH

3FTx parentmass 1118.459

Spectrum Analysis Report

Sequence Name:		Parentmass:	1118.459	Mass Error:	0.071	MH+ (mono):	1118.388
MH+ (avg):	1119.168	Threshold (a.i.):	0.000	Tolerance (Da):	0.700	Number of Peaks:	44
Above Threshold:	44	Assigned Peaks:	15	Not assigned Peaks:	29		

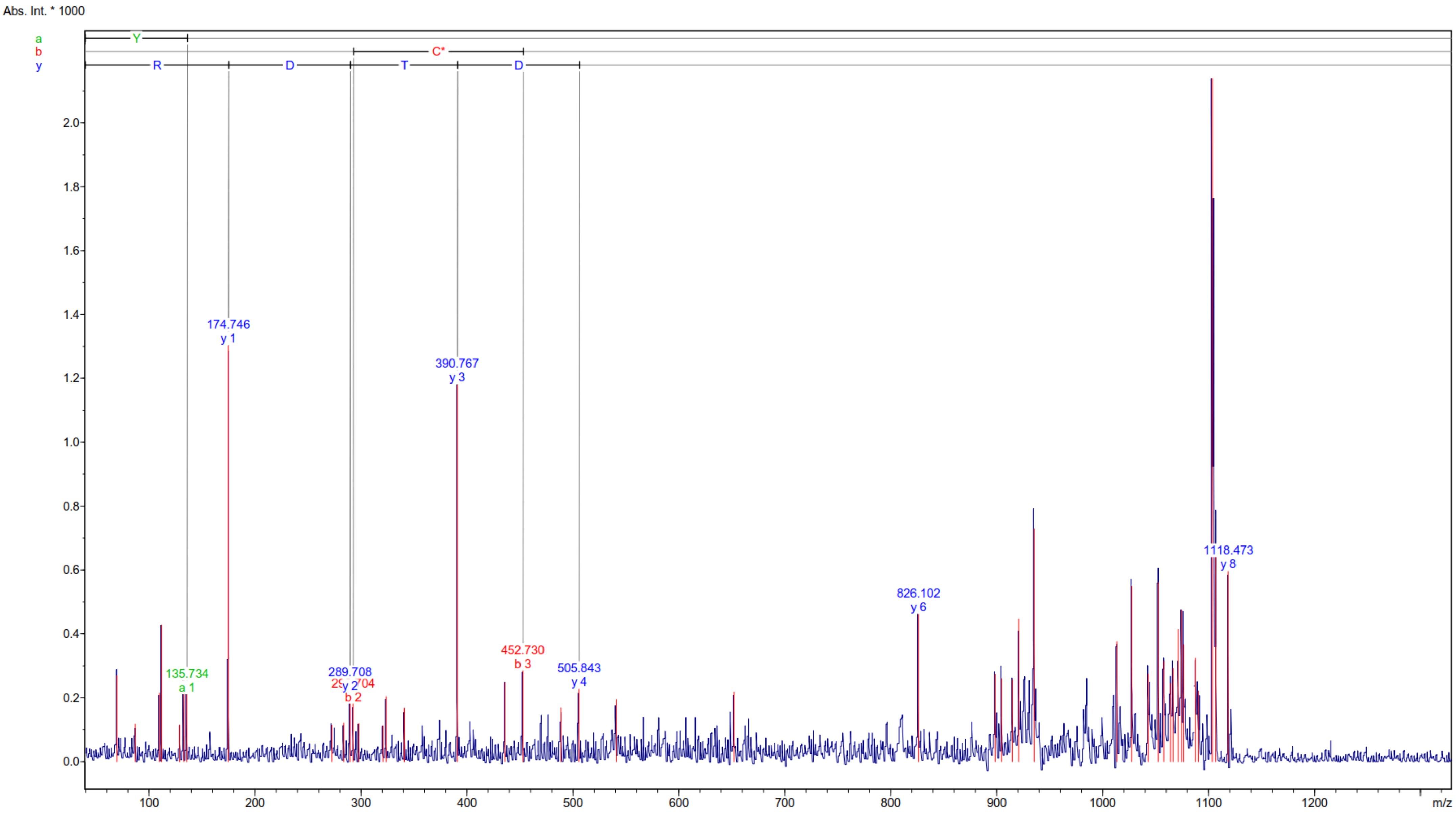

Copyright © 1999-2008 Bruker Daltonik GmbH

Spectrum Analysis Report

Display Parameter:

Parentmass:	1118.459	Mass Error:	0.071	MH+ (mono):	1118.388	MH+ (avg):	1119.168
Threshold (a.i.):	0.000	Tolerance (Da):	0.700	Number of Peaks:	44	Above Threshold:	44
Assigned Peaks:	15	Not assigned Peaks:	29				

Peaklist:

Peak	Mass	Intensity	Peak	Mass	Intensity	Peak	Mass	Intensity	Peak	Mass	Intensity	Peak	Mass	Intensity
1	69.803	269.169	2	86.826	117.571	3	109.742	214.827	4	111.781	427.759	5	128.792	114.594
6	132.705	266.152	7	135.734	210.610	8	174.746	1303.356	9	272.738	114.062	10	283.745	120.885
11	289.708	215.401	12	292.704	181.013	13	297.733	119.391	14	320.625	112.393	15	323.731	203.248
16	340.749	167.842	17	390.767	1181.407	18	435.694	247.740	19	452.730	285.506	20	488.787	168.645
21	505.843	228.099	22	540.847	194.811	23	651.941	218.496	24	826.102	461.865	25	898.645	274.336
26	904.755	259.915	27	914.703	256.181	28	920.790	447.162	29	935.339	729.268	30	1013.573	376.029
31	1027.367	549.117	32	1042.930	274.385	33	1052.458	561.553	34	1057.705	314.696	35	1063.894	245.854
36	1066.328	292.348	37	1071.267	414.438	38	1074.496	473.651	39	1076.654	366.779	40	1087.460	323.889
41	1090.190	221.745	42	1103.561	2413.247	43	1106.561	657.327	44	1118.473	596.768			

Calculated Masses:

YECCDTDR 3: Carbamidomethyl (C) 4: Carbamidomethyl (C)

N-Term.	Ion	a	a-17	a-18	b	b-17	b-18	b+18	c	i	x	y	z	C-Term.	Ion
1	Y	**136.076**	119.049	118.065	164.071	147.044	146.060	182.081	181.097	**136.076**	201.098	**175.119**	158.092	8	R
2	E	265.118	248.092	247.108	**293.113**	276.087	275.103	311.124	310.140	102.055	316.125	**290.146**	**273.119**	7	D
3	C*	425.149	408.122	407.138	**453.144**	**436.117**	**435.133**	471.154	470.170	**133.043**	417.173	**391.194**	374.167	6	T
4	C*	585.180	568.153	567.169	613.174	596.148	595.164	631.185	630.201	**133.043**	532.200	**506.221**	**489.194**	5	D
5	D	700.207	683.180	682.196	728.201	711.175	710.191	746.212	745.228	88.039	692.230	666.251	649.225	4	C*
6	T	801.254	784.228	783.244	829.249	812.223	811.239	847.260	846.276	74.060	852.261	**826.282**	809.255	3	C*
7	D	916.281	**899.255**	**898.271**	944.276	927.250	926.265	962.287	961.303	88.039	981.304	955.324	938.298	2	E
8	R	1072.382	1055.356	1054.372	1100.377	1083.351	1082.367	**1118.388**	1117.404	**129.113**	1144.367	**1118.388**	1101.361	1	Y

Copyright © 1999-2008 Bruker Daltonik GmbH

3FTx parentmass 1020.337

Spectrum Analysis Report

Sequence Name:		Parentmass:	1020.337	Mass Error:	-0.135	MH+ (mono):	1020.472
MH+ (avg):	1021.200	Threshold (a.i.):	0.000	Tolerance (Da):	0.700	Number of Peaks:	64
Above Threshold:	64	Assigned Peaks:	39	Not assigned Peaks:	25		

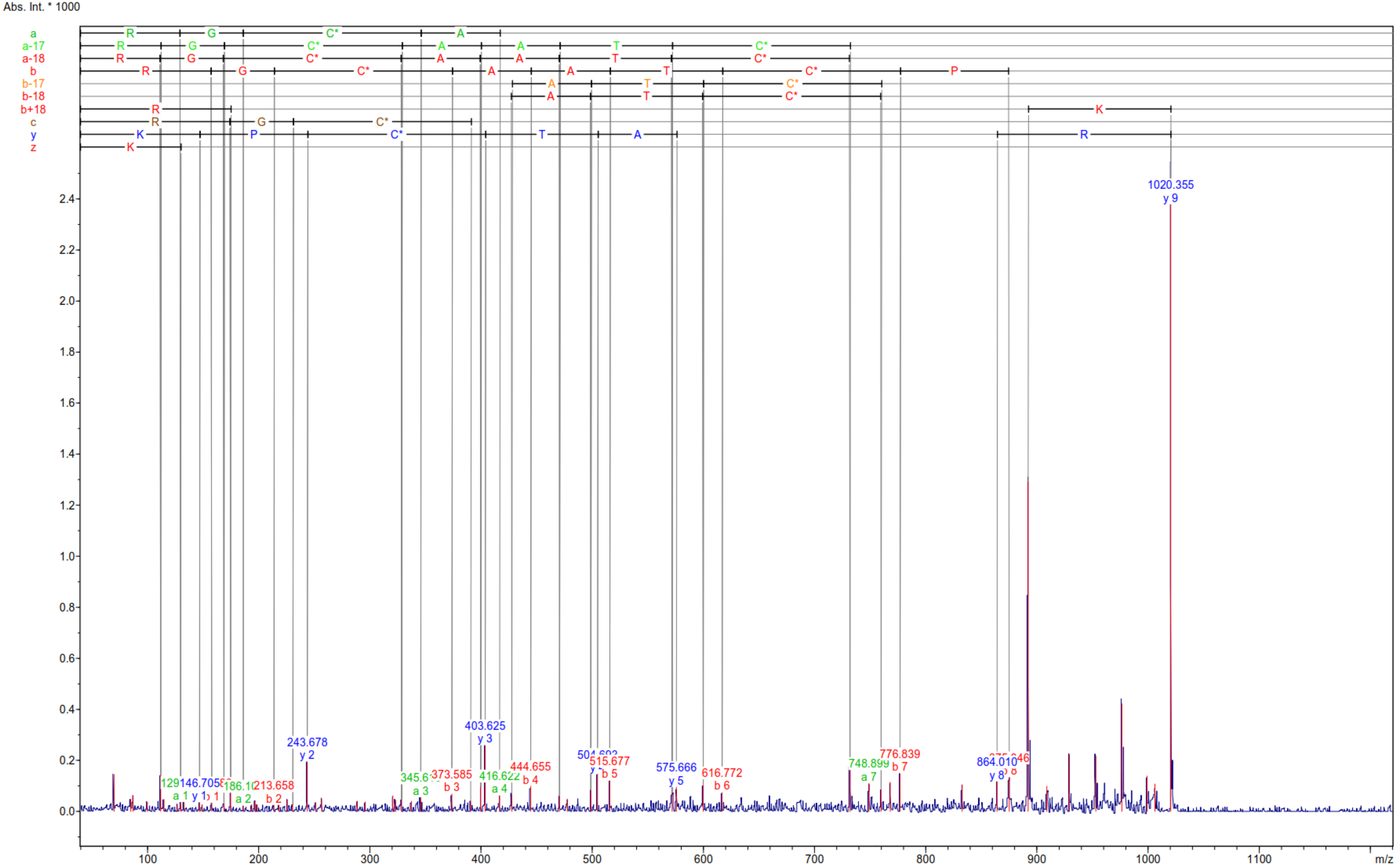

Copyright © 1999-2008 Bruker Daltonik GmbH

Spectrum Analysis Report

Display Parameter:

Parentmass:	1020.337	Mass Error:	-0.135	MH+ (mono):	1020.472	MH+ (avg):	1021.200
Threshold (a.i.):	0.000	Tolerance (Da):	0.700	Number of Peaks:	64	Above Threshold:	64
Assigned Peaks:	39	Not assigned Peaks:	25				

Peaklist:

Peak	Mass	Intensity	Peak	Mass	Intensity	Peak	Mass	Intensity	Peak	Mass	Intensity	Peak	Mass	Intensity
1	69.770	144.802	2	84.740	48.045	3	86.777	62.734	4	99.722	36.840	5	111.732	142.502
6	114.702	47.008	7	126.733	26.349	8	129.688	36.707	9	132.650	67.770	10	146.705	35.700
11	149.164	26.015	12	157.680	33.946	13	168.672	32.304	14	174.668	136.779	15	186.109	22.455
16	193.589	25.314	17	196.657	42.778	18	198.662	26.498	19	213.658	25.323	20	218.156	26.462
21	225.665	45.723	22	230.513	26.101	23	243.678	194.956	24	251.245	35.261	25	256.690	48.510
26	288.643	40.203	27	295.828	36.869	28	320.520	61.189	29	323.060	43.777	30	328.586	47.395
31	337.273	39.271	32	345.616	55.606	33	373.585	68.189	34	390.704	40.420	35	399.609	95.983
36	403.625	259.448	37	416.622	62.664	38	427.581	66.607	39	444.655	98.740	40	470.624	60.573
41	477.633	48.187	42	498.647	85.370	43	504.693	147.037	44	515.677	121.654	45	571.750	75.232
46	575.666	95.366	47	599.709	102.440	48	616.772	74.386	49	731.811	177.072	50	748.899	111.331
51	759.838	86.062	52	767.979	114.714	53	776.839	149.787	54	832.678	103.521	55	864.010	117.710
56	875.046	133.949	57	892.045	1291.449	58	908.913	98.592	59	929.219	222.523	60	952.981	217.247
61	976.288	422.563	62	999.086	138.402	63	1006.155	107.263	64	1020.355	2378.402			

Calculated Masses:
RGCAATCPK 3: Carbamidomethyl (C) 7: Carbamidomethyl (C)

N-Term.	Ion	a	a-17	a-18	b	b-17	b-18	b+18	c	i	x	y	z	C-Term.	Ion
1	R	**129.113**	**112.087**	**111.103**	**157.108**	140.082	139.098	**175.119**	**174.135**	**129.113**	173.092	**147.113**	**130.086**	9	K
2	G	**186.135**	**169.108**	**168.124**	**214.130**	**197.103**	**196.119**	232.140	**231.156**	30.034	270.145	**244.166**	227.139	8	P
3	C*	**346.166**	**329.139**	**328.155**	**374.160**	357.134	356.150	392.171	**391.187**	**133.043**	430.175	**404.196**	387.170	7	C*
4	A	**417.203**	**400.176**	**399.192**	**445.198**	**428.171**	**427.187**	463.208	462.224	44.049	531.223	**505.244**	488.217	6	T
5	A	488.240	**471.213**	**470.229**	**516.235**	**499.208**	**498.224**	534.245	533.261	44.049	602.260	**576.281**	559.254	5	A
6	T	589.287	**572.261**	**571.277**	**617.282**	**600.256**	**599.272**	635.293	634.309	74.060	673.297	647.318	630.292	4	A
7	C*	**749.318**	**732.292**	**731.308**	**777.313**	**760.287**	**759.302**	795.324	794.340	**133.043**	**833.328**	807.349	790.322	3	C*
8	P	846.371	829.344	828.360	**874.366**	857.339	856.355	**892.376**	**891.392**	**70.065**	890.349	**864.370**	847.344	2	G
9	K	974.466	957.439	956.455	1002.461	985.434	984.450	**1020.471**	1019.487	101.107	1046.451	**1020.471**	1003.445	1	R

Copyright © 1999-2008 Bruker Daltonik GmbH

3FTx parentmass 1319.692

Spectrum Analysis Report

Sequence Name:		Parentmass:	1319.692	Mass Error:	0.109	MH+ (mono):	1319.584
MH+ (avg):	1320.481	Threshold (a.i.):	0.000	Tolerance (Da):	0.700	Number of Peaks:	51
Above Threshold:	51	Assigned Peaks:	28	Not assigned Peaks:	23		

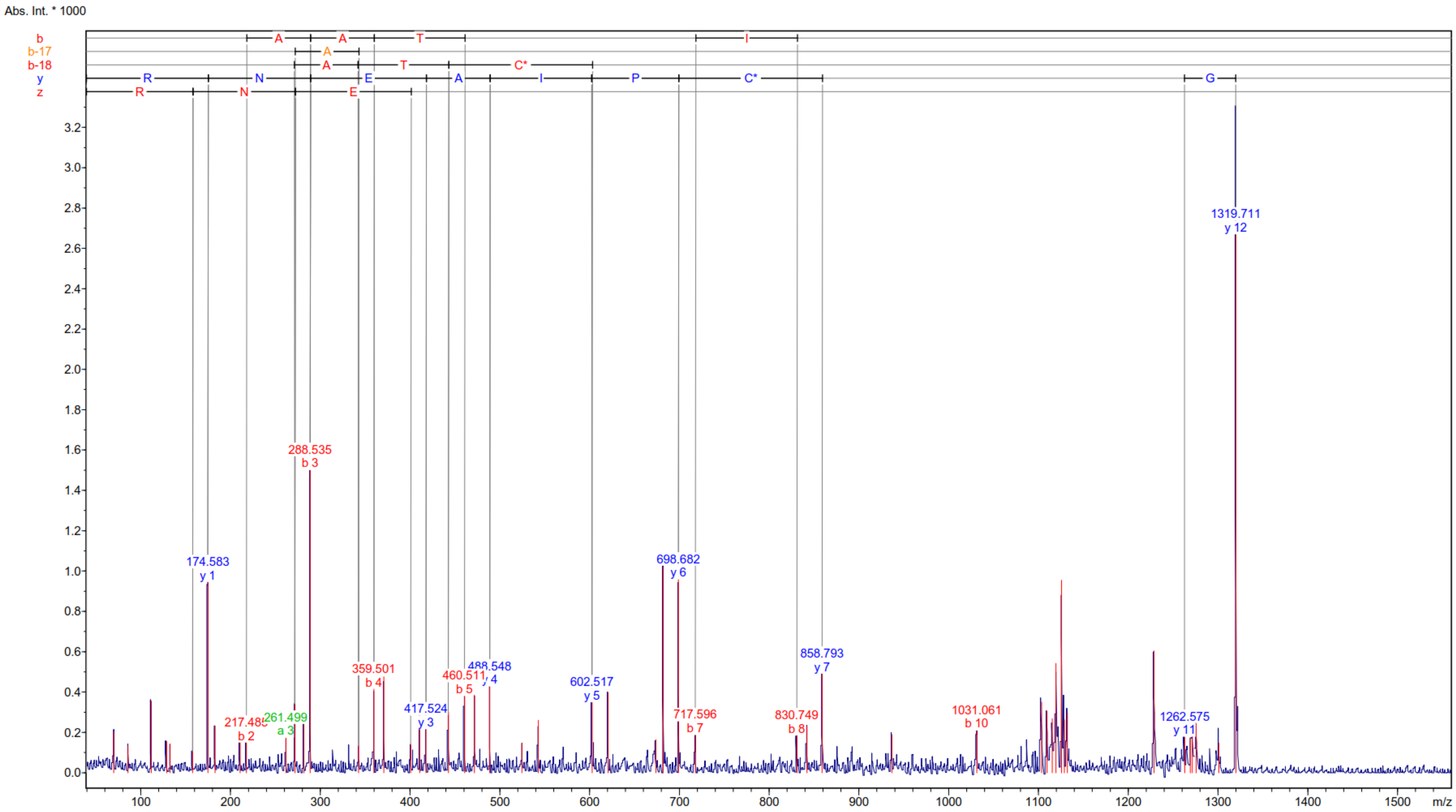

Copyright © 1999-2008 Bruker Daltonik GmbH

Spectrum Analysis Report

Display Parameter:

Parentmass:	1319.692	Mass Error:	0.109	MH+ (mono):	1319.584	MH+ (avg):	1320.481
Threshold (a.i.):	0.000	Tolerance (Da):	0.700	Number of Peaks:	51	Above Threshold:	51
Assigned Peaks:	28	Not assigned Peaks:	23				

Peaklist:

Peak	Mass	Intensity	Peak	Mass	Intensity	Peak	Mass	Intensity	Peak	Mass	Intensity	Peak	Mass	Intensity
1	69.685	202.634	2	85.677	134.798	3	111.637	356.834	4	128.682	156.328	5	132.539	144.573
6	157.588	105.448	7	174.583	945.366	8	182.604	233.172	9	210.575	192.651	10	217.485	148.804
11	261.499	173.469	12	271.542	341.525	13	281.561	281.664	14	288.535	1501.695	15	342.532	133.443
16	359.501	414.603	17	370.520	475.939	18	400.542	138.556	19	410.579	292.999	20	417.524	215.524
21	442.483	301.054	22	460.511	380.894	23	471.508	384.784	24	488.548	427.756	25	524.523	146.697
26	542.598	261.399	27	602.517	349.543	28	620.509	398.273	29	673.730	164.788	30	681.643	1020.264
31	698.682	958.186	32	717.596	186.939	33	830.749	185.720	34	841.804	233.459	35	858.793	490.704
36	936.407	188.807	37	1031.061	209.920	38	1103.306	350.657	39	1109.272	307.254	40	1115.233	268.674
41	1119.332	542.552	42	1125.567	955.701	43	1128.627	263.145	44	1131.870	297.233	45	1228.484	604.868
46	1262.575	178.225	47	1268.949	167.319	48	1271.191	210.797	49	1275.551	251.773	50	1300.658	147.245
51	1319.711	2669.704												

Calculated Masses:
GCAATCPIAENR 2: Carbamidomethyl (C) 6: Carbamidomethyl (C)

N-Term.	Ion	a	a-17	a-18	b	b-17	b-18	b+18	c	i	x	y	z	C-Term.	Ion
1	G	30.034	13.007	12.023	58.029	41.002	40.018	76.039	75.055	30.034	201.098	**175.119**	**158.092**	12	R
2	C*	190.064	173.038	172.054	**218.059**	201.033	200.049	236.070	235.086	**133.043**	315.141	**289.162**	**272.135**	11	N
3	A	**261.102**	244.075	243.091	**289.097**	**272.070**	**271.086**	307.107	306.123	44.049	444.184	**418.204**	**401.178**	10	E
4	A	332.139	315.112	314.128	**360.134**	**343.107**	**342.123**	378.144	377.160	44.049	515.221	**489.242**	472.215	9	A
5	T	433.186	416.160	415.176	**461.181**	444.155	**443.171**	479.192	478.208	74.060	628.305	**602.326**	585.299	8	I
6	C*	593.217	576.190	575.206	621.212	604.185	**603.201**	639.223	638.238	**133.043**	725.358	**699.378**	682.352	7	P
7	P	690.270	**673.243**	672.259	**718.265**	701.238	700.254	736.275	735.291	**70.065**	885.388	**859.409**	**842.383**	6	C*
8	I	803.354	786.327	785.343	**831.349**	814.322	813.338	849.359	848.375	**86.096**	986.436	960.457	943.430	5	T
9	A	874.391	857.364	856.380	902.386	885.359	884.375	920.396	919.412	44.049	1057.473	**1031.494**	1014.467	4	A
10	E	1003.434	986.407	985.423	**1031.428**	1014.402	1013.418	1049.439	1048.455	102.055	**1128.510**	1102.531	1085.504	3	A
11	N	1117.476	1100.450	1099.466	1145.471	**1128.445**	1127.461	1163.482	1162.498	87.055	1288.541	**1262.562**	1245.535	2	C*
12	R	1273.578	1256.551	1255.567	1301.573	1284.546	1283.562	**1319.583**	1318.599	**129.113**	1345.562	**1319.583**	1302.557	1	G

Copyright © 1999-2008 Bruker Daltonik GmbH
